# Interpretable Machine Learning Models and Symbolic Regressions Reveal Transfer of Per- and Polyfluoroalkyl Substances (PFASs) in Plants: A New Small-Data Machine Learning Method to Augment Data and Obtain Predictive Equations

**DOI:** 10.3390/toxics13070579

**Published:** 2025-07-10

**Authors:** Yuan Zhang, Yanting Li, Yang Li, Lin Zhao, Yongkui Yang

**Affiliations:** 1 School of Environmental Science and Engineering, Tianjin University, Tianjin 300350, China; yz685604@gmail.com (Y.Z.); li_yanting666@163.com (Y.L.); liy0128@163.com (Y.L.); zhaolin@tju.edu.cn (L.Z.); 2 Georgia Tech Shenzhen Institute, Tianjin University, Shenzhen 518071, China

**Keywords:** machine learning, data augmentation, symbolic regression, PFAS bioaccumulation, quantitative prediction

## Abstract

Machine learning (ML) techniques are becoming increasingly valuable for modeling the transport of pollutants in plant systems. However, two challenges (small sample sizes and a lack of quantitative calculation functions) remain when using ML to predict migration in hydroponic systems. For the bioaccumulation of per- and polyfluoroalkyl substances, we studied the key factors and quantitative calculation equations based on data augmentation, ML, and symbolic regression. First, feature expansion was performed on the input data after data preprocessing; the most important step was data augmentation. The original training set was expanded nine times by combining the synthetic minority oversampling technique and a variational autoencoder. Subsequently, the four ML models were applied to the test set to predict the selected output parameters. Categorical boosting (CatBoost) had the highest prediction accuracy (*R*^2^ = 0.83). The Shapley Additive Explanation values indicated that molecular weight and exposure time were the most important parameters. We applied three symbolic regression models to obtain accurate prediction equations based on the original and augmented data. Based on augmented data, the high-dimensional sparse interaction equation exhibited the highest accuracy (*R*^2^ = 0.776). Our results indicate that this method could provide crucial insights into absorption and accumulation in plant roots.

## 1. Introduction

Robust predictive modeling is imperative for advancing risk assessments, informing regulatory framework development, and formulating effective and sustainable mitigation strategies for emerging contaminants [1,2]. Comprehensive research on the uptake and internal transport of contaminants is crucial for accurately delineating the potential risks to both ecosystems and human health [3]. Root concentration factor (RCF) modeling is important for elucidating the complex dynamics of plant–contaminant interactions [4]. The RCF characterizes the accumulation of a contaminant in the roots of a plant in relation to its concentration in an exposure medium [5]. Among the emerging contaminants, perfluoroalkyl substances (PFASs) pose unique challenges. Compared to 4700 compounds with a wide range of molecular sizes, structures, and functional groups [6], PFASs exhibit extensive heterogeneity, complicating our understanding of their environmental behavior [7,8,9]. This diversity underscores the importance of elucidating the mechanisms by which PFASs are absorbed and accumulated in plant roots.

Since the 1970s, numerous modeling studies have attempted to correlate the physicochemical properties of contaminants with their uptake by plants. Early approaches relied primarily on the octanol/water partition coefficient as the single predictive parameter. However, this single-parameter strategy is inadequate for PFASs, resulting in low accuracy and limited applicability across different plant species and PFAS variants [10,11,12,13]. Subsequently, more advanced compartmental models have been developed to incorporate a wider range of physicochemical and environmental factors to capture the complexity of the contaminant uptake and translocation processes. Despite these advances, these models still face challenges in accurately predicting PFAS behavior, largely because of the unique properties of PFASs, such as their ionic form and variable environmental interactions [14,15].

Machine learning (ML) has experienced a substantial surge in popularity in environmental research due to its efficacy in addressing multivariate problems [16,17]. For example, an RCF database with 246 data points was built for 57 organic compounds in 11 crops, using 15 chemical, soil, and plant features to develop four ML models, i.e., a fully connected neural network (FCNN), gradient-boosted regression trees (GBRT), random forest (RF), and support vector regression (SVR) [18]. FCNN and GBRT performed best, with *R*^2^ values of 0.79 and 0.76 and mean absolute error values of 0.22 and 0.23, respectively.

The prediction of transport factors faces two major challenges. First, “small data,” which can be defined as insufficient sample sizes or sample-to-feature ratios below the recommended thresholds [19], directly constrain the predictive ability of ML models in characterizing the transport, dispersion, and distribution of chemicals in the environment. Second, although ML models can achieve highly certain predictive performance, they inherently face interpretability challenges. While post-interpretation methods, such as Shapley Additive Explanation (SHAP) values, have made progress in revealing feature importance and contributions, these techniques provide only approximate explanations of model behavior rather than precise mathematical expressions. From a strictly scientific paradigm, the lack of explicit mathematical expressions makes validating and generalizing the relationship between the model and phenomenon through a theoretical framework challenging. This limits our in-depth understanding of the underlying mechanisms to some extent.

We conducted data augmentation of PFAS transport in the roots of hydroponic plants by combining the synthetic minority oversampling technique (SMOTE) and a variational autoencoder (VAE) model to address the challenges of small data and the low transparency of PFAS translocation studies in hydroponics and solve these two bottlenecks. Three symbolic regression methods were adopted to determine the mathematical equations with the highest predictive accuracy. The specific objectives of this study were to (1) develop a specialized SDML workflow tailored to target values, especially in terms of data augmentation, (2) find the best ML model to predict PFAS translocation in hydroponics, (3) quantify the relative contributions of key drivers affecting the translocation of PFASs in hydroponics, and (4) establish mathematical equations to estimate the target values for different PFASs and plant species from selected quantifiable properties. Therefore, this study presents a new small-data ML method to augment data and obtain predictive equations. RF was used in this study. In addition to using three mainstream integrated models (categorical boosting (CatBoost), the light gradient boosting machine (LightGBM) and extreme gradient boosting (XGBoost)) were used to explore the key drivers for prediction using SHAP analysis. Finally, symbolic regression was implemented to enhance the availability of predictive models using mathematical equations.

## 2. Materials and Methods

### 2.1. Data Preprocessing, Derived Feature Construction, and Selection for log RCF

#### 2.1.1. Data Description and Preprocessing

A comprehensive experimental dataset including 616 data points, representing different plants with diverse RCFs, was compiled to predict the plant uptake of PFASs from hydroponics using experimental data from peer-reviewed publications using the Web of Science and Google Scholar from 2009 to 2024, as shown in Figure S1 and Table S1. The features included in this dataset were used as the base features. An iterative imputation algorithm based on an RF regressor [20] was used to fit and impute the data and address the issue of missing data for the target variables in the dataset. Subsequently, the quartiles and interquartile range of the data were calculated, and the range of abnormal values was determined based on the 1.5 times interquartile range (1.5 IQR) rule. A logarithmic transformation was implemented to enhance the symmetry of the data and their propensity to approach a normal distribution, where the trimmed data demonstrated a substantial positive bias (skewness > 1). The detailed construction process is presented in TEXTs S1 and S2, and the encoded variables are listed in Table S2. Finally, the two data sources were integrated to construct a more comprehensive molecular feature space. The chemical identifiers (CIDs) and published physicochemical properties of the compounds were obtained using the PubChem application programming interface (API) [21]. We used the open-source RDKit Python (https://www.rdkit.org/docs/index.html, accessed on 6 September 2024) API and the Ray framework [22] to enable the parallel computation and extraction of a variety of structural features. Finally, the entire dataset was divided into training (75%) and testing (25%) sets.

#### 2.1.2. Derived Feature Construction and Selection

Additional meaningful features must be generated to enhance the model’s performance. Moreover, the predictive capability of the original data is augmented by constructing the derived features through a series of nonlinear transformations and interactive constructions [23]. The detailed construction process is presented in TEXT S3.

Feature selection is a critical step in ML and data mining [24,25]. In this study, classical statistics (TEXT S4) were leveraged (including the F-statistic for linear relationships [26], mutual information for nonlinear dependencies [27], and distance correlation for generalized statistical dependencies [28]) to generate the initial indicators of feature relevance. These measures were then normalized, weighted, and combined to yield a comprehensive feature-importance score. This method further integrates stability analysis through bootstrap sampling to determine the consistency of feature importance [29] across different subsets and solves the problem of multicollinearity by penalizing redundant features by using a variance inflation factor (VIF) [30]. Furthermore, the introduction of the maximum information coefficient (MIC) facilitates the detection of complex nonlinear relationships [31], whereas the ReliefF algorithm enhances the discriminative power of features by considering local instance-based assessments [32]. The pseudocode for the design of the entire algorithm is presented in TEXT S5.

### 2.2. Data Augmentation for log RCF Using Stratified Variational Regression

We combined three data augmentation methods: hierarchical and box-splitting strategies, VAE generation, and SMOTE interpolation. We maintained the original distribution characteristics and effectively solved the sample imbalance problem in each interval by dynamically evaluating the sample distribution of each box and customizing the augmentation ratio. This approach is more robust and adaptive. First, it uses adaptive binning technology [33] to divide the target space into multiple statistical regions and automatically selects a binning strategy based on data skewness. We ensured that each range contained a similar number of samples for unevenly distributed data to maintain statistical balance. For more evenly distributed data, ranges of equal size were created to preserve the physical meaning of the RCF intervals, making the results more interpretable from an environmental perspective. The number of bins was precisely determined through a trade-off between the data scale and bin granularity.

(1) $$n_{bins}=max\left(6,min\left(12,n_{train}/12\right)\right)$$

Each box captured the local statistical characteristics of the target variable, including the number of samples, mean, standard deviation, and value range. We also evaluated the balance of the sample distribution within each range to determine how much additional data generation was needed.

The algorithm used a dual-pipeline generation strategy to create new samples. SMOTE was the first pipeline used [34]. The synthetic minority oversampling technique for regression (SMOTER) was used to identify similar samples in the feature space and perform intelligent interpolation among them, with particular consideration given to the continuous distribution characteristics of the log RCF. This process creates new chemical property combinations by blending the characteristics from similar existing compounds while adding small variations to increase diversity. This mimicked the natural variation in chemical properties within structural families, which can be formally represented as

(2) $$x_{new}=x_{i}+\mu \left(x_{nn}-x_{i}\right)+\delta ,\;\mu \sim U\left(0,1\right)$$

Among them, δ is a slight noise related to the features, which is added proportionally to maintain the scale relationship between the features. The second pipeline utilized a VAE [35] to learn the underlying patterns in the chemical data and generate new samples that follow these patterns but represent novel chemical combinations. This method captures complex nonlinear relationships between molecular properties that simple interpolation may miss. This generative approach ensures that new samples maintain realistic relationships between different molecular descriptors, while expanding the chemical space coverage.

The data generation process was iterative and self-monitored. After each round of sample generation, we evaluated whether the new data maintained realistic chemical relationships and provided a better representation across different RCF ranges. This ensured that our enhanced dataset had more samples and preserved the fundamental chemical and biological relationships that govern the plant uptake of PFASs. The pseudocode for the entire algorithm design is presented in TEXT S6.

### 2.3. Development of ML Model for log RCF

#### 2.3.1. ML Models

We implemented four types of algorithms that are considered the most effective for improving the prediction accuracy in this study [36]. The CatBoost regressor [37], a sophisticated gradient-boosting implementation, has demonstrated superior performance in predicting PFAS bioaccumulation in groundwater [34] through its innovative ordered boosting algorithm, which minimizes prediction shifts arising from target leakage. CatBoost can be computationally intensive for large datasets and may require extensive hyperparameter tuning to achieve optimal performance. The LightGBM [38] uses a histogram-based decision-tree growth strategy that discretizes continuous features into optimal bins, dramatically reducing memory requirements while preserving statistical fidelity. However, LightGBM is sensitive to small datasets and may suffer from overfitting when the number of samples is limited relative to the features. The XGBoost algorithm [39] addresses high-dimensional sparsity and multicollinearity in the molecular descriptor space through its distinctive regularization framework, which combines L1 and L2 penalties. Despite its strengths, XGBoost is prone to overfitting noisy data and requires careful parameter tuning to balance the bias and variance trade-offs. The RF model [40] builds a low-correlation decision tree integration by combining bootstrap aggregation with random feature subset selection at each node split, thereby providing robustness against overfitting and inherent variance reduction through ensemble averaging. However, RF may struggle with extrapolation beyond the training data range and may be biased toward features with more levels of categorical variables. The detailed differences are listed in Table S3.

#### 2.3.2. Hyperparameter Search Using Bayesian Search

The hyperparametric optimization methodology used in this study utilizes a multilevel model-tuning strategy implemented through different parameter space constructions and Bayesian searches [36,41]. This approach is based on the following three key aspects:

First, an appropriate search space was constructed based on the characteristics of the parameters. Linear or logarithmic scales were used for continuous parameters, depending on the magnitude range. For example, parameters that spanned multiple orders of magnitude, such as the learning rate, were represented using log-uniform sampling. In contrast, parameters with narrower ranges, such as subsamples, were sampled uniformly.

Second, the method accounts for logical dependencies between the parameters. In the event of logically incompatible parameter combinations, invalid combinations can be avoided by introducing conditional branches into the search space. Additionally, tree-structured Parzen estimator (TPE) methods [21,42] have been used to efficiently handle these conditional hyperparameter spaces, particularly for tree architecture optimization.

Finally, Bayesian optimization [43] was used to guide the search process. The Bayesian approach was distinguished from traditional grid or random search by constructing probabilistic models between parameters and model performance and by using historical search results to guide subsequent exploration. The pseudocode for the algorithm design is shown in TEXT S7.

### 2.4. Model Interpretability Using SHAP Analysis

The interpretation framework quantified the contribution of each feature to the prediction using the Shapley value [44,45] as a core indicator. A three-level calculation strategy was implemented for all tree models. First, we attempted to invoke the model’s native methods to obtain the marginal contribution value of the features in each sample directly. If unavailable, we applied TreeExplainer [46], which utilizes the split paths inside the tree structure and leaf weights to obtain an accurate Shapley value through combined computation. This greatly improved the efficiency compared to the violent computations. When the first two methods proved ineffective, the system transitioned to kernelExplainer, a kernel-function-based approximation computation method [47], which approximates the behavior of a complex model by constructing a local linear model and optimizes the distribution of background samples through k-means clustering.

### 2.5. Establishment of Empirical Simulation Equations for Predicting log RCF

We used three different symbolic regression approaches to develop interpretable prediction equations that could provide insights into the mechanisms underlying PFAS uptake by plants. These methods automatically discover mathematical relationships from data while maintaining physical and chemical interpretability, which is crucial for understanding how molecular properties influence bioaccumulation processes.

#### 2.5.1. Genetic Programming (GP) Symbolic Regression

This model applies GP principles [48] to represent mathematical expressions as tree structures and automatically constructs optimal equations through simulated natural selection. We built expression trees using a function set and optimized candidate solutions through evolutionary operations (crossover, mutation, and elevation). The implementation used the gplearn library with a population size of 2000 and 100 generations and parallel processing to efficiently explore the solution space.

#### 2.5.2. Multilayer Feature Transfer Equation Construction (MFTEC)

MFTEC prioritizes chemically meaningful features before constructing the derived interactions. First, the molecular descriptors that are most relevant to RCFs were identified. We then explored how these key properties interacted to influence plant uptake. The derived interactions in MFTEC have a clearer physical meaning because they are built from preselected chemically relevant features. This approach ensures that the derived features correspond to the actual physicochemical phenomena rather than to mathematical artifacts.

#### 2.5.3. High-Dimensional Sparse Interaction Equation (HSIE)

The HSIE comprehensively explores the molecular property interactions that may influence the RCF before selecting the most important ones. This method can capture complex multifactor effects that are common in environmental systems. By considering higher-order interactions, the HSIE can identify when combinations of molecular properties create uptake behaviors that differ from simple additive effects.

The three approaches differ in how they handle the derived interactions and their physical interpretability. GP can discover any mathematical relationship but may create derived terms that lack environmental relevance. MFTEC builds interactions from chemically important features, ensuring that derived terms like (hydrophobicity × surface area) represent meaningful physicochemical processes. The HSIE initially considers all possible interactions and then identifies which combinations truly matter for RCF prediction.

**Figure 1:**
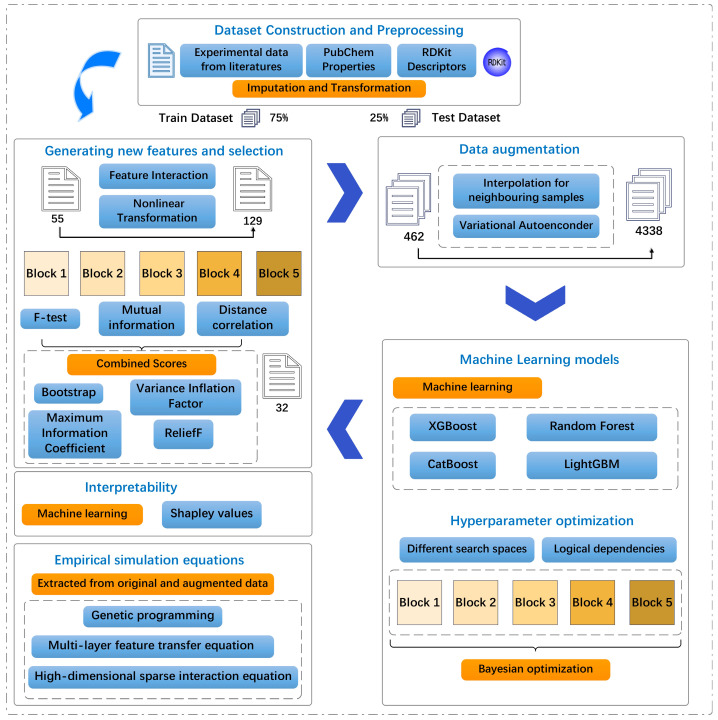
Schematic diagram of machine learning (ML) applied in this work.

The interactions identified by these methods provide insights into the mechanistic basis of plant PFAS uptake. Simple additive models assume that molecular properties independently influence RCF; however, real environmental systems often exhibit synergistic or antagonistic effects. These nonadditive effects appear as interaction terms in the equations. These symbolic regression approaches provide complementary strategies for understanding how the molecular structure governs PFAS root concentration factors. This mechanistic understanding is essential for environmental risk assessment, as it allows for the prediction of the uptake potential of new PFAS compounds based solely on their molecular structure without requiring extensive experimental testing. A flowchart of the study is shown in Figure 1.

## 3. Results and Discussion

### 3.1. Based and Derived Features of PFASs Constructed Based on Empirical Formula Performance and Selection

**Figure 2:**
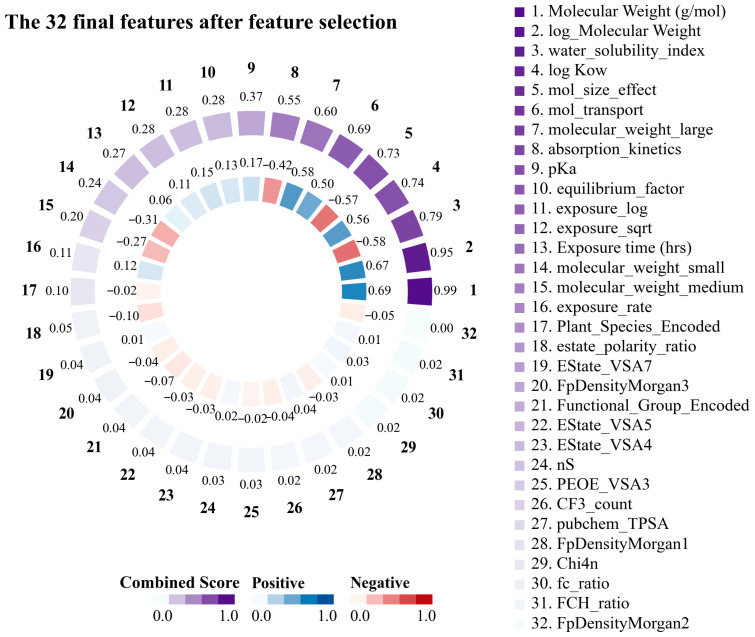
Importance score and correlation with log root concentration factor (RCF) for the top 20 features for root uptake and accumulation of per- and polyfluoroalkyl substances (PFASs) in plants.

Striking relationships were identified between the key structural and physicochemical features of the PFAS by examining the correlation matrix. The 32 features obtained through feature engineering are shown in Figure 2. This demonstrated that the molecular weight of PFASs emerged as the most important feature in predicting translocation behavior in plants, exhibiting a correlation of 0.69 with the log RCF and a composite score of 0.98. logKow exhibited a moderate positive correlation (0.57) with log RCF, with a characteristic significance of 0.02 and a composite score of 0.74, indicating that the hydrophobicity of PFASs significantly affected their transport behavior. Conversely, the water solubility index, which is based on the topological polar surface area (TPSA)/(Molecular Weight × (1 + 0.5 × log Kow)), is a composite feature. This index had a significant predictive value (feature significance of 0.26) and integrated multiple key parameters that affect the water solubility of PFASs. The data substantiated this feature’s robust negative correlation (−0.58) with the target variable, indicating that higher-molecular-weight PFASs with lower water solubility accumulate more easily in plant roots [49,50]. In addition, particular consideration should be given to the interaction of exposure time when designing kinetic features. Absorption kinetics was based on the following equation: (1 − exp (−0.005 × Exposure time)). This is further supported by the correlation of −0.42 with the target variable and a composite score of 0.56. This suggests that this feature captured the time-dependent and hydrophobic barriers to PFAS absorption. In summary, the advantages of molecular weight as a predictor, combined with the significant contributions of hydrophobicity and water solubility indices, indicate that PFAS transport in hydroponic plant systems follows a visible structure–activity relationship. By elucidating these structure-dependent transport mechanisms, we can better predict PFAS’s environmental mobility and bioaccumulation potential.

### 3.2. Statistical Analysis Between Original and Augmented Data for the Training Set

**Figure 3:**
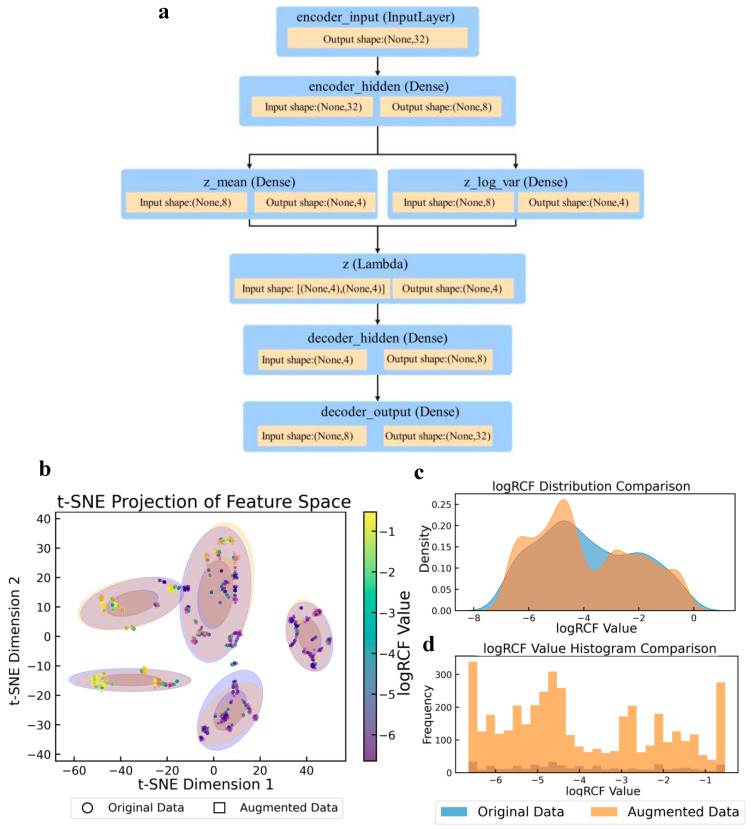
Variational autoencoder (VAE) model structure and comparison of log RCF for PFASs in plants before and after data augmentation. (**a**) VAE model structure. (**b**) t-Distributed stochastic neighbor embedding (t-SNE) projection of feature spaces. (**c**) Log RCF distribution. (**d**) Log RCF histogram comparison.

The augmentation method used in this study addresses the core challenges of regression modeling scenarios with limited sample sizes and complex data distributions. A statistical comparison of the target variables before and after augmentation is presented in Table S4. The structure of the VAE model used in the enhancement method is shown in Figure 3a. This model achieved effective dimensionality reduction and retained key information regarding the data features. It also enhanced the generation ability and data diversity of the model using a random sampling mechanism of mean and variance. The t-distributed stochastic neighbor embedding (t-SNE) [51] feature space projection diagram (Figure 3b) revealed the topological preservation ability and local density optimization effect of the data augmentation algorithm. The multiple discrete cluster structures formed by the original data (purple) were effectively retained by the enhanced data (orange). Concurrently, the enhanced samples effectively occupied the low-density regions within and surrounding the clusters, thereby markedly enhancing sample coverage without inducing unnatural clusters. The multi-peak nature of the log RCF distribution plot indicated that the target variable may have originated from multiple underlying mechanisms. Furthermore, the enhancement algorithm effectively preserved this intricate distribution structure through adaptive binning [52]. The density curve (Figure 3c) demonstrates that the augmentation process achieved moderate distribution smoothing. The original data (blue) appears to have a more concentrated distribution with a notable peak around −3, while the augmented data (orange) shows a wider, more spread-out distribution with multiple peaks around −5, −3, and closer to −1. This demonstrated that the augmentation process effectively expanded the distribution to cover the entire range of log RCF values, particularly by enhancing the representation of previously underrepresented regions. The histogram (Figure 3d) further illustrates this transformation with the frequency counts before and after augmentation. The orange bars (augmented data) show significantly higher counts across the entire range of log RCF values, with particularly strong representation at the extremes (−6 and −1) and the middle range (−4). This confirmed that the data augmentation strategy successfully increased the sample size while maintaining the general shape of the original distribution but enhanced coverage in previously sparse areas.

### 3.3. Different Model Predictions for log RCF of PFASs in Plants

**Figure 4:**
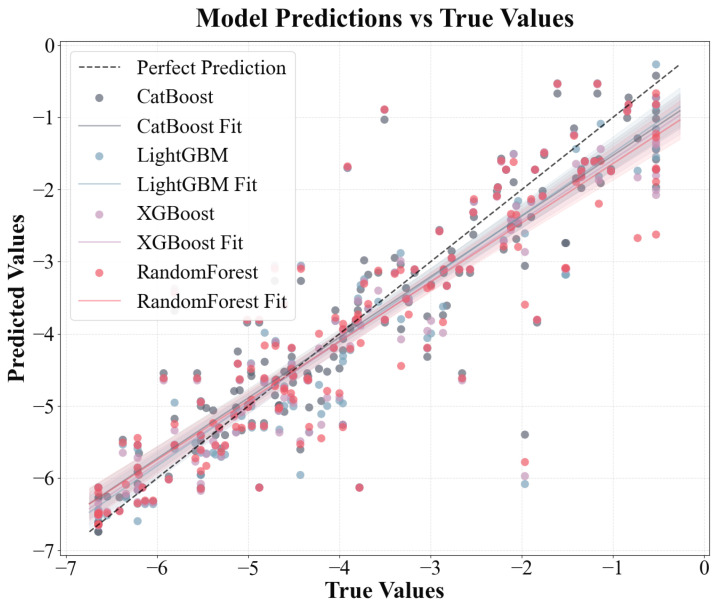
Predicted data vs. original experimental data for four ML models for log RCF.

The performances of multiple ML models developed through multisource data fusion were evaluated by combining experimental data from the literature, physicochemical properties calculated using the RDkit, and chemical descriptors retrieved from PubChem. The data analysis results (Figure 4 and Table 1) present a performance comparison of the four gradient boosting and ensemble learning models (CatBoost, LightGBM, XGBoost, and RF) for the original and enhanced datasets. Specifically, the RMSE of the CatBoost model decreased from 0.7401 to 0.6906 (an improvement of 6.7%) in the five-fold cross-validation and from 0.8015 to 0.7401 (an improvement of 7.7%) in the test set. Its performance indicators increased from 0.8224 to 0.8564 (cross-validation) and from 0.8012 to 0.8300 (test set), after which the RMSE of the LightGBM model decreased by approximately 7.9% (cross-validation) and 9.9% (test set) and its performance improved by approximately 5.2% (cross-validation) and 5.1% (test set). XGBoost performed better after the data enhancement. The RMSE decreased by 10.8% in the cross-validation and 7.8% in the test set, with performance improvements of approximately 6.9% (in the cross-validation) and 4.1% (in the cross-validation and test sets). Although the RF also improved, the improvement was relatively small. The RMSE of the test set decreased by only 1.9% and the RMSE of the cross-validation decreased by 8.8%. Overall, all models achieved a double improvement after using the enhanced data: reduced prediction errors and enhanced model prediction accuracy, with the CatBoost, LightGBM, and XGBoost models exhibiting superior performance in comparison to the RF model on both the original and augmented sets; the CatBoost model provided the optimal fitting results with *R*^2^ = 0.8300, followed by the LightGBM model with *R*^2^ = 0.8249. This discrepancy can be attributed to the fundamental differences in the model mechanisms. CatBoost, LightGBM, and XGBoost use gradient boosting methods to optimize each iteration’s objective function by gradually correcting the previous prediction residuals [53]. This allowed them to capture the complex interactions and nonlinear relationships between features more precisely. This characteristic affords gradient boosting methods a notable advantage when confronted with subtle data patterns, culminating in substantial enhancements in the evaluation metrics. Conversely, RF predominantly relies on the independent construction of multiple decision trees and subsequent result averaging to reduce model variance and lacks a continuous correction mechanism for incorrect predictions [28]. A detailed comparison of the predictive results of the four models is shown in Figure S2.

### 3.4. Identification of Different Important Features for Different Predictive Models of log RCF

**Figure 5:**
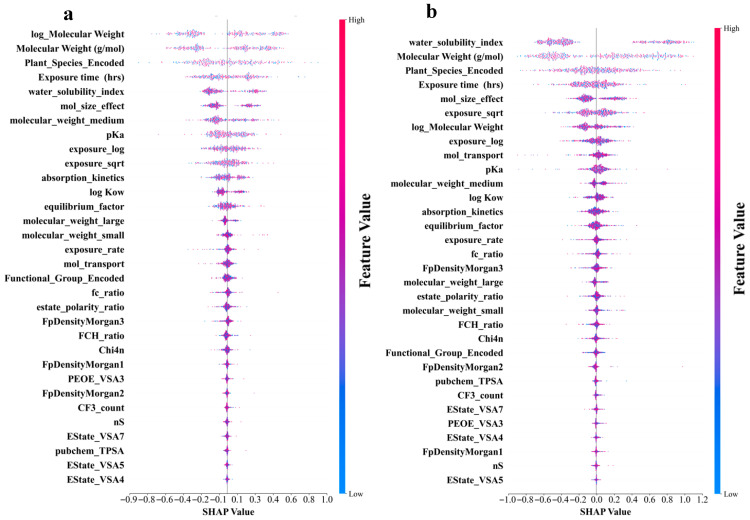

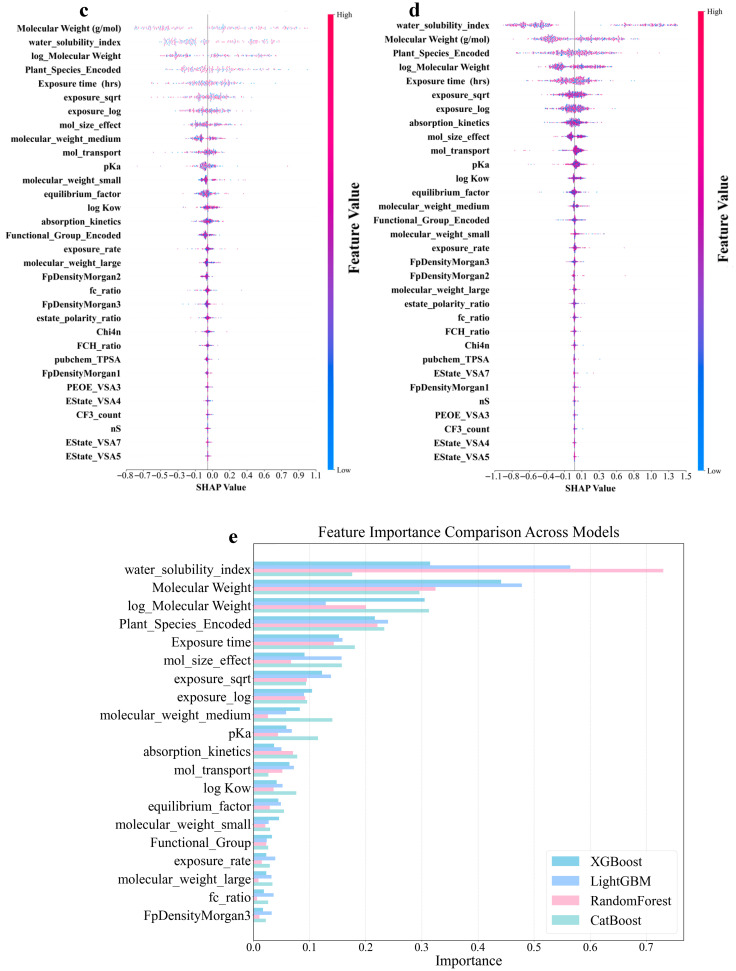
Shapley Additive Explanation (SHAP) values and feature-importance comparison of original and derived features in four ML models for log RCF of PFASs in hydroponics. (**a**) Categorical boosting (CatBoost). (**b**) Light gradient-boosting machine (LightGBM). (**c**) Gradient boosting (XGBoost). (**d**) Random forest. (**e**) Top 20 features comparison for predicting log RCF using four ML models.

Figure 5a–d shows that the molecular structure and physicochemical properties were the dominant predictors across all models. Specifically, plant species and molecular weight-related features demonstrated consistently high absolute SHAP values, indicating their strong influence on the predictions. Furthermore, the exposure time and pKa ranked relatively high as secondarily important factors in all the models. In contrast, the degree of contribution of the electrotopological state (estate)-related characteristics was the lowest in all models. In addition, the different models leverage features in distinct ways. CatBoost relies on unique ordered target statistics and a symmetrical tree structure. In terms of molecular weight characteristics, the value range was moderate (−0.7 to 0.8), and the point distribution was dense and orderly. The feature SHAP distribution range of LightGBM was the widest, especially showing a significant color gradient change in the water solubility index (−1.1 to 1.2), reflecting its tendency to maximize the information gain of a single feature based on the leaf-first strategy. XGBoost exhibited a characteristic distribution between the two. The random model with the lowest prediction accuracy exhibited the most extreme feature differentiation. The SHAP values of the plant species features were abnormally dispersed (−1.0 to 1.3), and the low-ranked features had almost no SHAP contribution. This “black-or-white” evaluation model stems from the simple averaging mechanism of its basic decision tree and the random feature subsampling strategy. The lack of fine adjustment ability of the gradient optimization process for the relationships between subtle features resulted in fewer gradient transitions and a greater aggregation of extreme values, as shown in Figure 5d. An analysis of the feature-importance results for the four models is shown in Figure 5e. All four ML models in this study were assigned extremely high importance scores for molecular weight-related features, which were consistent with the results of the SHAP analysis. In addition, the reliance of the RF on the water solubility index remained the most extreme (0.73), much higher than its assessment of molecular weight (0.32). This pattern, which is overly dominated by a single feature, may cause it to ignore the multifactor synergy effect, thereby becoming a possible reason for its low prediction accuracy. These findings revealed unique feature interpretation perspectives using different algorithmic architectures. This provided a multidimensional empirical basis for understanding the complex relationships between molecular properties and biological effects in prediction models.

### 3.5. Developing Mathematical Models to Estimate log RCF Values

We adopted three symbolic regression methods and explored the changes in the prediction accuracy of the data before and after enhancement to quantitatively analyze the factors influencing log RCF. The GP model (*R*^2^ = 0.466) for the original data generated highly nonlinear mathematical expressions characterized by complex hierarchical structures and nested functional transformations (Equation (3)). Furthermore, this model (*R*^2^ = 0.556) was used to construct a sophisticated mathematical expression (Equation (4)). This equation incorporates double-nested absolute-value operations around the sine functions. The expression features higher-order exponents of log Kow and pKa, such as (log Kow)^6^ and (pKa)^8^, which mathematically amplify minor variations in these parameters, potentially capturing biological systems in which slight molecular modifications trigger disproportionate bioaccumulation responses. However, the equation’s complexity (combining trigonometric, power, square root, and absolute-value operations) creates a “black-box” effect where individual parameter contributions cannot be isolated, limiting mechanistic interpretability. These factors are intertwined through logarithmic operations, power relationships, and multilayer sine functions, thereby reflecting the complexity of the interaction between multiple factors in the uptake of PFASs by plant roots. In summary, incorporating nonlinear and periodic modulation terms in the model underscored the sensitivity of PFAS transport efficiency in plant roots to even minor parameter alterations, potentially corresponding to critical behaviors, such as membrane permeability, ion dissociation, and the balance of intracellular and extracellular distribution within physicochemical processes [50,51,54].

(3) $$logRCF=log\left(0.05\right)-\sqrt{{\left(logK_{OW}\right)}^{9}+{\left(pKa\right)}^{8}}+MW$$

(4) $$logRCF=MW+sin\left(sin\left(MW+\left(-0.519+{\left(logK_{ow}\right)}^{6}\right)\right)\right)+log\left(0.05\right)\;-\sqrt{sin\left(MW\right)-{\left(pKa\right)}^{8}}$$

In Equations (3) and (4), ‘MW’ stands for ‘Molecular Weight (g/mol)’.

**Figure 6:**
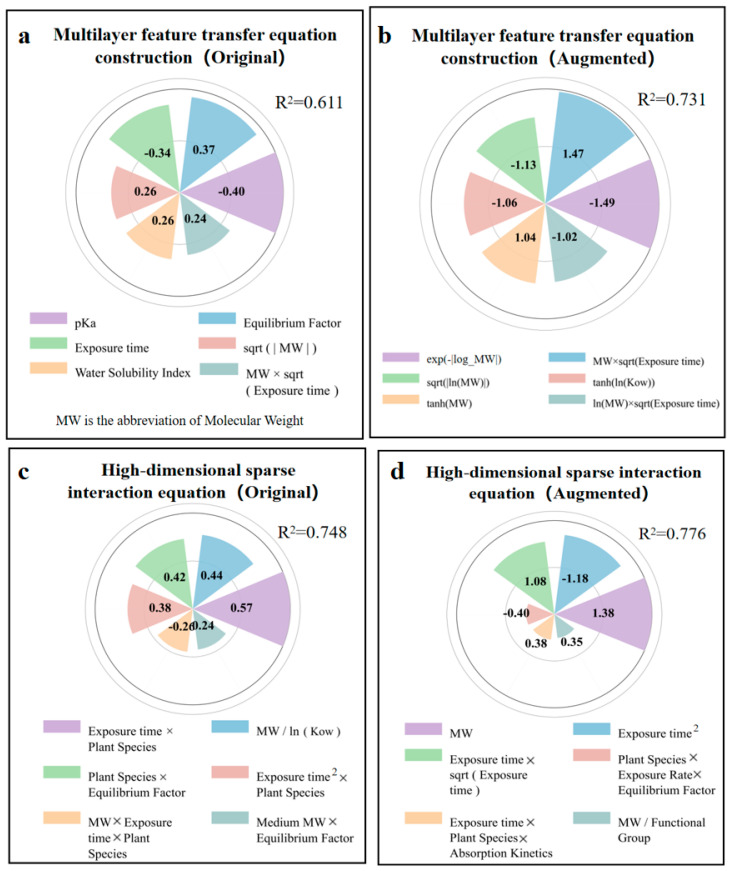
Coefficients from multilayer feature transfer equation construction (MFTEC) and high-dimensional sparse interaction equation (HSIE) in the original and augmented dataset for predicting log RCF. (**a**) Top 6 coefficients for MFTEC in the original data. (**b**) Top 6 coefficients for MFTEC in the augmented data. (**c**) Top 6 coefficients for HSIE in the original data. (**d**) Top 6 coefficients for HSIE in the augmented data.

The MFTEC (*R*^2^ = 0.611) model (Figure 6a) balanced the mathematical complexity with interpretability, producing multivariate polynomial equations with selectively applied transformations (Formula (S1)). The model formed a mathematical structure that combined linear, interactive, and simple nonlinear components. The strongest positive coefficients appeared in exposure metrics (Equilibrium factor: 0.37) and sqrt(|MW|) (0.26), while the most significant adverse effects emerged from pKa (−0.4) and exposure time (−0.34). The augmented data model (*R*^2^ = 0.731) (Figure 6b) was expanded to 46 terms with a sophisticated mathematical structure including quadratic terms, logarithmic transformations, and exponential decay functions (Formula (S2)). The model captures the opposing directional effects of raw and transformed parameters. The inclusion of paired interaction terms with their squared counterparts (MW × sqrt(Exposure time): 1.47 versus ln(MW) × sqrt(Exposure time): −1.02) created mathematical inflection points where the adverse nonlinear effect balanced the positive linear effect.

The HSIE model based on the original data (*R*^2^ = 0.748) (Figure 6c) revealed that the interaction between the exposure time and plant species squared (0.57) had the strongest positive correlation, indicating that plant characteristics exhibited nonlinear cumulative effects during the exposure periods. The molecular weight-to-log Kow ratio (0.44) was the second most influential factor, demonstrating that bioaccumulation followed scale-invariant relationships based on relative molecular properties rather than absolute values. The molecular-weight interaction between exposure time and plant species (−0.26) exhibited a negative correlation, indicating that larger molecules showed reduced bioaccumulation efficiency in certain plant-exposure combinations. The HSIE model, based on enhanced data (*R*^2^ = 0.776) (Figure 6d), showed improved predictive performance through refined parameterization. The molecular-weight term (1.38) became the dominant positive factor, indicating that absolute molecular size plays a crucial role in bioaccumulation. The opposing trends between the negative exposure time-squared term (−1.18) and positive exposure time-square root interaction (1.08) revealed a biphasic bioaccumulation pattern in which the initial rapid uptake transitions to diminishing root concentrations due to translocation to aerial plant parts during prolonged exposure. The interaction between exposure time, plant species, and absorption kinetics (0.38) demonstrated that specific plant species developed enhanced absorption efficiency over extended exposure periods, likely reflecting adaptive physiological responses such as increased membrane permeability or upregulated transporter activity.

Overall, GP had the poorest performance owing to its tree-structure expression generation method based on evolutionary algorithms, which led to overly complex formulas and formed a black-box effect. MFTEC strikes a balance between complexity and interpretability. It first selects important features and then creates derivative features. Moreover, it adopts a stacked integration architecture combined with multiple regressions to form a polynomial equation structure, enabling it to deliver better performance at medium complexity. However, its limitation lies in potentially missing some complex nonlinear relationships that do not fit well within its predetermined transformation functions, particularly when dealing with parameters that exhibit threshold effects or non-monotonic responses. The HSIE adopts the opposite strategy. It first creates derivative features and then selects. This ensures that all possible mathematical relationships are captured, enabling the HSIE to achieve optimal predictive performance by efficiently processing high-dimensional data that contain third-order polynomial features and transformation functions. Its main limitations are the computational intensity required for feature generation and the risk of overfitting when applied to smaller datasets, which can capture noise rather than true biological relationships when there are insufficient data points. The differences between these three methods indicated that balancing the sequence of feature generation and selection, handling nonlinear relationships, and maintaining the interpretability of the equation are the key factors affecting the performance of the final model when constructing a prediction model.

These results provide quantitative support for elucidating the enrichment mechanisms of PFASs in hydroponically grown plants. They emphasized the need to consider the complex interactive effects among the influencing factors when developing environmental risk assessments and pollution remediation strategies.

## 4. Conclusions

This study established a set of methods based on data-enhanced ML and symbolic regression to evaluate the key factors and quantitative relationships between the bioaccumulation of PFASs in plants. The predictive equations obtained from the three symbolic regression methods provide a unique opportunity to estimate potential PFAS accumulation in plant roots. In conclusion, the findings of this study demonstrate the potential of data augmentation, ML, and symbolic regression as valuable tools for predicting the uptake and accumulation of PFASs in plants. First, we used an existing PFAS dataset related to log RCF for hydroponics. The dataset was augmented by incorporating various methodologies combining SMOTE and VAE under strict quality control to generate a new dataset with 4338 points of experimental data. Additionally, we used four ML techniques on the original and augmented datasets: CatBoost, LightGBM, XGBoost, and RF. Plant species and molecular weight-related features demonstrated consistently high absolute SHAP values, indicating their strong influence on the predictions. Furthermore, the exposure time and pKa ranked relatively high as secondarily important factors in all the models. Finally, through symbolic regression, it was found that the HSIE (*R*^2^ = 0.776) had the best prediction performance. For each model, the prediction accuracy based on the amplified data was higher than that based on the original data. ML models with hyperparameters may be overly complex and difficult to apply in practice. However, mathematical expressions obtained through symbolic regression can compensate for this shortcoming. This is important for sustainable applications in precision agriculture and environmental monitoring. Practical applications of our modeling framework include PFAS risk assessment through rapid RCF prediction, strategic plant selection to minimize root-to-shoot translocation, and agricultural decision support for crop management in contaminated areas. Future research should focus on integrating multi-omics data (transcriptomics, proteomics, and metabolomics) to validate the molecular mechanisms underlying our predictive models and to enhance biological interpretability. In addition, expanding the framework to include plant metabolic transformation pathways and developing real-time monitoring systems for PFAS bioaccumulation in agricultural settings would further advance the practical applications of this methodology. Additionally, it is imperative to emphasize that while ML is poised to serve as a powerful tool for environmental investigation, implementing ML is not a standalone solution, but rather a complement to extensive model interpretation. This approach is essential to elucidate the underlying mechanisms of complex processes, thereby facilitating a more profound understanding of the phenomena under investigation.

## Figures and Tables

**Table 1:** Key parameter results of diverse models for root uptake and accumulation of PFASs in hydroponics.

Models	Dataset		*R* ^2^	RMSE
CatBoost	Validation	Original	0.8224	0.7401
		Augmented	0.8564	0.6906
	Test	Original	0.8012	0.8015
		Augmented	0.8300	0.7401
LightGBM	Validation	Original	0.8032	0.7793
		Augmented	0.8449	0.7178
	Test	Original	0.7851	0.8334
		Augmented	0.8249	0.7512
XGBoost	Validation	Original	0.7953	0.7902
		Augmented	0.8503	0.7050
	Test	Original	0.7827	0.8380
		Augmented	0.8147	0.7727
RandomForest	Validation	Original	0.7913	0.8029
		Augmented	0.8386	0.7321
	Test	Original	0.7713	0.8597
		Augmented	0.7790	0.8438

## Data Availability

The data supporting the findings of this study are available from the corresponding author upon reasonable request.

## Supplementary Materials

The following supporting information can be downloaded at: https://www.mdpi.com/article/10.3390/toxics13070579/s1. TEXT S1: Random forest regressor to impute data. TEXT S2: 1.5 IQR rule. TEXT S3: Assumptions for Generating New Features. TEXT S4: Features correlation. TEXT S5: Features selection. TEXT S6: Data augmentation. TEXT S7: Hyperparameter optimization. Figure S1: Distribution of PFAS Compounds and Plant Species in source dataset. Figure S2: Correlation analysis. Table S1. Statistical summary of the numeric basic variables comprising the PFAS uptake and translocation compiled 616 point dataset. Table S2. Encoded variables. Table S3. Key differences between the four models. Table S4. Statistical comparison of target variable before and after augmentation. Appendix S1. Main python code. References [55,56] are cited in the Supplementary Materials.
