# Supplementary material for: Interpretable Machine Learning Models and Symbolic Regressions Reveal Transfer of Per- and Polyfluoroalkyl Substances (PFASs) in Plants: A New Small-Data Machine Learning Method to Augment Data and Obtain Predictive Equations
Source: Toxics. 2025 Jul 10;13(7):579. doi: 10.3390/toxics13070579 (PMC12300769; doi:10.3390/toxics13070579)
Supplement: Supplementary file 1 [file toxics-13-00579-s001.zip › toxics-3712534-supplementary.pdf]

Supplementary Material

Interpretable Machine Learning Models and Symbolic Regressions Reveals Root Uptake and Accumulation of Per- and Polyfluoroalkyl Substances (PFAS) in plants: A New Small-Data Machine Learning Method to Augment Data and Obtain Predictive Equations

TEXT S1 : Random forest regressor to impute data

The core principle of iterative multivariate interpolation lies in using the intrinsic correlation between variables to construct a prediction model to fill in the missing values, which treats the missing value processing as a supervised learning problem, and trains a prediction model (RandomForestRegressor in this case) to estimate the missing values by treating each feature with a missing value in turn as a dependent variable and the other features as independent variables, and in Each iteration uses the result of the previous round of filling to further optimize the prediction, forming a feedback loop, which makes the filled values gradually converge to the optimal solution; the advantage of this method is that it can retain the complex interrelationships among variables, and retain the statistical properties and intrinsic patterns of the data better than the simple mean or median filling.

TEXT S2 : 1.5 IQR rule

1.5 IQR rule determines the threshold boundaries for outliers based on the quartiles of the data, in this paper this rule is applied by calculating the first quartile (q1) and the third quartile (q3) of the target variable y (RCF), then solving for the quartile IQR (i.e., q3-q1), and setting the lower bound at q1-1. 5IQR and the upper bound at q3+1.5IQR, and any observation falling outside of those boundaries values are labeled as outliers.

TEXT S3 : Assumptions for Generating New Features

In addition to incorporating the relevant physical and chemical properties of PubChem and RDKit, we also constructed some empirical formulas to explore the interactions between characteristics. The feature engineering function **process_feature_engineering_chunk** receives an input feature matrix and generates multiple derived features, focusing on several key aspects:

- Exposure time transformation features

$$\mathrm{exposure\_log} = \ln(1+\mathrm{time}) \tag{S1}$$

$$\mathrm{exposure\_sqrt} = \sqrt{\mathrm{time}} \tag{S2}$$

$$\mathrm{exposure\_rate} = 1 - e^{-0.05\times \mathrm{time}} \tag{S3}$$

These transformations captured the non-linear effects of exposure time, particularly the logarithmic transform reduces skewness, the square root moderately compresses high values, and the exponential saturation rate simulated the process of bioaccumulation reaching equilibrium.

- Molecular properties and transmembrane transport-related features

$$\mathrm{mol\_transport} = \frac{\log K_{OW}}{\sqrt{MW}} \tag{S4}$$

$$\mathrm{mol\_size\_effect} = \frac{\ln(1+MW)}{1+|\log K_{OW}|} \tag{S5}$$

$$\mathrm{membrane\_permeability\_logkow} = \frac{\log K_{ow}}{\log(MW)} \tag{S6}$$

The function also creates molecular weight classification features:

mw_small: MW≤300；mw_medium:300<MW≤500；mw_large: MW>500

In (S4), the K_{ow} was used to measure the tendency of a compound to accumulate in a lipid environment, and the square root of the molecular weight was used to correct for the restriction on the diffusion rate imposed by the larger molecular size, thereby reflecting the property that more hydrophobic and smaller molecules were more likely to pass through cell membranes(Montal and Mueller, 1972). The construction of (S5) was used to capture the effect of the combined action of molecular size and hydrophobicity when transporting PFAS in hydroponically grown plants. (S6) used the logarithmic conversion of molecular weight to model the nonlinear decrease in membrane permeability in the higher molecular weight range.

- Fluorination and carbon atom interaction features

$$\text{CF\_interaction} = \text{nF} \times \text{nC} \qquad \text{S7}$$

$$\text{fc\_ratio} = \frac{\text{nF}}{\text{nC} + 10^{-6}} \qquad \text{S8}$$

$$\text{flurination\_degree} = \frac{\text{nF}}{\text{NumAtom} + 10^{-6}} \qquad \text{S9}$$

(S7) multiplied the number of fluorine atoms (nF) in a compound by the number of carbon atoms (nC) and was intended to reflect the potential impact of C-F interactions. We assume that the C-F interaction is likely to be more significant when a molecule has both a high number of carbon and fluorine atoms.

- Polarity and hydrogen bonding characteristics

$$\text{polarity\_index} = \text{TPSA} \times (\text{HBondDonorCount} + \text{HBondAcceptorCount}) \qquad \text{S10}$$

$$\text{hbond\_balance} = \frac{\text{HBondDonorCount}}{\text{HBondAcceptorCount} + 10^{-6}} \qquad \text{S11}$$

$$\text{polar\_lipophilic\_ratio} = \frac{\text{TPSA}}{|\text{XLogP}| + 1} \qquad \text{S12}$$

(S10) multiplied the total polar surface area (TPSA) of the molecule by the total number of hydrogen bond donors and acceptors, reflecting the overall polarity level of the molecule. A larger TPSA and more hydrogen bond sites generally mean that the molecule has a greater ability to interact in the aqueous phase(Potts et al., 2021). (S11) described the balance between the number of hydrogen bond donors and acceptors in a molecule. (S12) provided a combined measure of the polarity and lipophilicity of a molecule. A high polar-lipophilic ratio indicates that the molecule has relatively prominent polar characteristics.

- Molecular connectivity and complexity metrics

$$\begin{aligned}\text{connectivity\_complexity} &= \text{Chi0n} + 0.7 \times \text{Chi1n} + 0.5\text{Chi2n} + 0.3 \times \text{Chi3n} \\ &\quad + 0.1 \times \text{Chi4n}\end{aligned} \qquad \text{S13}$$

$$\text{branching\_index} = \frac{\text{Chi3n}}{\text{Chi1n} + 10^{-6}} \qquad \text{S14}$$

(S13) constructed by summing the weighted Chi-indices of molecules of different orders: Chi0n reflects the number of atoms in the molecule and the most basic connectivity information, while Chi1n to Chi4n capture the subtle changes in molecular structure from simple chains to more complex branched structures. (S14) measured the ratio of the degree of molecular branching to that of the straight-chain structure through the ratio of the third-order connectivity index Chi3n to the

first-order connectivity index Chi1n.

- LogP consensus estimates and deviations

$$\text{log\_consensus} = \frac{\text{CrippenLogP} + \text{XLogP}}{2} \quad \text{S15}$$

$$\text{logp\_deviation} = |\text{CrippenLogP} - \text{XLogP}| \quad \text{S16}$$

$$\text{crippen\_polarity\_index} = \frac{\text{CrippenMR}}{|\text{CrippenLogP}| + 1} \quad \text{S17}$$

In (S15), logP values calculated using two different methods (CrippenLogP and XLogP) to providing a comprehensive hydrophobicity index. Meanwhile, (S16) reflects the inconsistency between different algorithms by calculating the absolute difference between the two logP values. This deviation information can help reveal complex or abnormal features that may exist in the molecular structure. In addition, we constructed (S17), which divides CrippenMR by (abs(CrippenLogP)+1) to comprehensively evaluate the balance between the molecule's polarizing ability and hydrophobicity.

- E-state and polarity ratios

$$\text{estate\_range} = \max(\text{Estate\_VSA}) - \min(\text{EState\_VSA}) \quad \text{S18}$$

$$\text{estate\_mean} = \text{mean}(\text{EState\_VSA}) \quad \text{S19}$$

$$\text{estate\_polarity\_ratio} = \frac{\sum_{\text{polar}} \text{E} - \text{States} + 10^{-6}}{\sum_{\text{non-polar}} \text{E} - \text{States} + 10^{-6}} \quad \text{S20}$$

Firstly, (S18) was obtained by calculating the difference between the maximum and minimum values in the estate_cols column, which reflects the degree of dispersion of these characteristic values. Then, (S19) was constructed from the average of these columns to represent the overall level. Consequently, estate_polarity_ratio was constructed by summing the polar_estates column, which represents the polar characteristics, and dividing it by the nonpolar_estates column (both adding a minimal value to avoid division by zero). Finally, by summing the polar_estates columns and dividing the result with the nonpolar_estates columns. (S20) was constructed, which quantitatively reveals the relative ratio between polar and nonpolar properties of the molecule.

- Stereochemical complexity features

$$\text{stereo\_complexity} = \frac{\text{AtomStereoCount} + \text{BondStereoCount}}{\text{HeavyAtomCount} + 1} \quad \text{S21}$$

$$\begin{aligned}\text{stereo\_hbond\_index} &\\ &= (\text{AtomStereoCount} + \text{BondStereoCount}) \\ &\times (\text{HBondDonorCount} + \text{HBondAcceptorCount})\end{aligned} \quad \text{S22}$$

(S21) quantified the complexity of molecular stereochemistry by dividing the number of all stereocenters in a molecule by the number of heavy atoms, while (S22), obtained by multiplying the total number of stereocenters by the total number of hydrogen bond donors and acceptors after ensuring that hydrogen bond donor and acceptor information was included in the data, further models the molecular synergistic effect between stereocomplexity and its potential for hydrogen bonding interactions.

- Absorption kinetics and equilibrium factors

$$\text{adsorption\_kinetics} = \left(1 - e^{-0.005\times\text{time}}\right) \times e^{-0.2\times\log K_{ow}} \quad \text{S23}$$

$$\text{equilibrium\_factor} = 1 - e^{-\frac{\text{time}}{100+20\times\log K_{ow}}} \quad \text{S24}$$

$$\text{water\_solubility\_index} = \frac{\text{TPSA}}{\text{MW} \times (1 + 0.5\log K_{ow})} \quad \text{S25}$$

Firstly, a metric (S23) was constructed to describe the molecular absorption kinetics, in which $1\text{-}e^{-0.005\times\text{Exposure time (hrs)}}$ describes the kinetic behavior of the absorption process showing a gradual saturation with the increase of the exposure time, whereas with $e^{-0.2 \times \log \text{Kow}}$ modulated this process, reflecting the attenuating effect of molecular hydrophobicity (higher $\log K_{ow}$ indicates more hydrophobicity) on absorption. Next, the constructed (S24), also in the form of an exponential decay, describes the rate at which the molecule reaches equilibrium under certain exposure conditions by the ratio of exposure time to a scale regulated by $\log K_{ow}$, the tendency of the system to progressively converge to equilibrium as the exposure time increases. Finally, we constructed the (S25), TPSA, which measured the polarity of the molecule, and usually higher polarity implies better water solubility, while molecular weight and $\log K_{ow}$ were used to modulate this index, with higher molecular weights and hydrophobicity tending to reduce water solubility.

TEXT S4 : Features correlation

The Pearson's correlation coefficient between two features x and y is defined as:

$$r_{xy} = \frac{\text{cov}(x, y)}{\sigma_x \sigma_y} = \frac{\sum_{i=1}^{n}(x_i - \bar{x})(y_i - \bar{y})}{\sqrt{\sum_{i=1}^{n}(x_i - \bar{x})^2}\sqrt{\sum_{i=1}^{n}(y_i - \bar{y})^2}} \quad \text{S26}$$

where $\text{cov}(x, y)$ denotes the covariance between x and y, σ_x and σ_y represent the standard deviations of x and y respectively, and $\bar{x}$ along with $\bar{y}$ correspond to the sample means of x and y.

The correlation coefficient, r_{xy}, ranges from -1 to 1, with the former indicating a perfect negative correlation (as x increases, y decreases steadily) and the latter denoting a perfect positive correlation (as x increases, y increases steadily). A value of -1 corresponds to a perfect negative correlation, 0 indicates no linear correlation, and 1 corresponds to a perfect positive correlation. The computation of r_{XY} entails the utilization of the covariance of x and y, along with their respective standard deviations (σ_x and σ_y), and the incorporation of the sample means. This calculation quantifies the strength and direction of the linear relationship between the two variables.

TEXT S5 : Features selection:

Algorithm S1 SelectFeaturesParallel

```
function SELECTFEATURESPARALLEL(X,y,threshold,n_chunks)
    n_chunks ← min(n_chunks, ⌊|X|/min_samples⌋)
    {(X_i, y_i)}_{i=1}^{n_chunks} ← Partition(X, y) into n_chunks chunks
    {(F_i, MI_i, D_i)}_{i=1}^{n_chunks} ← Parallel computation of scores for each chunk
    F_total ← (1/n_chunks) Σ_{i=1}^{n_chunks} F_i,  MI_total ← (1/n_chunks) Σ_{i=1}^{n_chunks} MI_i,  D_total ← (1/n_chunks) Σ_{i=1}^{n_chunks} D_i
    F_norm ← F_total/max(F_total),  MI_norm ← MI_total/max(MI_total),  D_norm ← D_total/max(D_total)
    S_0 ← 0.4F_norm + 0.3MI_norm + 0.3D_norm          ▷ Initial combine scores
    S_1 ← 0.6S_0 + 0.4Bootstrap stability scores
    S_2 ← S_1 ⊙ (1 − VIF penalty)          ▷ Multicollinearity adjustment
    S_3 ← 0.75S_2 + 0.25MIC scores
    S_4 ← 0.7S_3 + 0.3ReliefF scores
    S_5 ← S_4 ⊙ Low variance penalty factor
    θ ← max(S_{final,1−r}, threshold · max(S_final))          ▷ r = 0.4: target selection ratio
    F_selected ← {f_j | S_{final,j} > θ}
    min_features ← max(5, ⌊0.1 · |X_j|⌋)
    max_features ← ⌊0.5 · |X_j|⌋
    if |F_selected| < min_features then
        F_selected ← top − min_features features by S_final
    else if |F_selected| > max_features then
        F_selected ← top − max_features features by S_final
    end if
    return F_selected
end function
```

TEXT S6 : Data augmentation:

Algorithm S2 Stratified Regression Data Augmentation

```
function AugmentRegressionData(X_train, y_train, η_noise, r_target)
    Calculate target statistics : μ_y, σ_y, min_y, max_y, skew_y
    if |skew_y| > 1or too few unique values then
        Create bins using adaptive quantile binning
    else
        Create bins using equal-width binning
    end if
    Calculate bin counts and distribution
    Identify categorical and numerical features
```

Initialize augmented data:$X_{augmented} = X_{train}, y_{augmented} = y_{train}$
Standardize numerical features:$X_{scaled} = StandardScaler(X_{numeric})$
Build VAE model:input_dim → encoding_dim → latent_space → input_dim
VAE loss = reconstruction loss + KL divergence loss
Train VAE model (200 epochs) and extract encoder and decoder
for iteration=1 to 50 do
for each bin bin_i from 1 to n_{bins} do
Calculate samples to add samples_to_add_i for this bin
Extract X_{bin} and y_{bin} for the current bin
for j=1 to n_{smote} do
Randomly select one neighbor from k-nearest neighbors
Generate interpolation coefficient μ~U（0，1）
$X_{new} = X_i + \mu \times (X_{nn} - X_i) + small\ noise,\ y_{new} = y_i + \mu \times (y_{nn} - y_i) + small\ noise$
Add synthetic sample:$X_{augmented} \cup \{X_{new}\}, y_{augmented} \cup \{y_{new}\}$
end for
for k=1 to n_{vae} do
Randomly select base sample: (X_{base}, y_{base}) from bin
Get latent representation:$z_{base} = encoder(X_{base})$
Add perturbation to latent vector:$z_{new} = z_{base} + N(0,0.15)$
Decode from latent space:$X_{numeric,new} = decoder(z_{new})$
Generate target:$y_{new} = y_{base} + N(0, \sigma_{bin} \times 0.02)$
Add synthetic sample to augmented dataset
end for
end for
if $|X_{augmented}| \geq |X_{train}| \times target\_ratio$ then
break
end if
end for
Compare original vs. Augmented distribution statistics
if mean difference > 10% or std difference> 20% then
Apply distribution correction:$y_{augmented} = \frac{y_{augmented} - \mu_{aug}}{\sigma_{aug}} \times \sigma_{orig} + \mu_{orig}$
end if
return $X_{augmented}, y_{augmented}$
end function

<u>TEXT S7 : Hyperparameter optimization</u>:

Algorithm S3 Hyperparameter optimization

function GridSearchModels($X_{train}, y_{train}, param\_grids, cv$)
best_models ← ∅, best_params ← ∅
$for\ (model\_name, param\_grid) \in param\_grids\ do$
$if\ model\_name = 'CatBoost'\ then$
$iterations \leftarrow max(param\_grid.iterations), param\_grid \leftarrow$

```
param_grid\ {iterations}
        best_score ← −∞, best_model ← null, best_param ← null
        for params ∈ ParameterGrid(param_grid) do
            param ← params ∪ {iterations: iterations, random_seed: 42}
            model ← CatBoostRegressor(params)
            r2, rmse, mae ← EvaluateModel(model, X_train, y_train, cv)
            if r2 > best_score then
                best_score ← r2, best_param ← params
                model.fit(X_train, y_train), best_model ← model
            end if
        end for
    else                                    ▻ XGBoost, LightGBM, RandomForest
        search_spaces ← ConvertToSearchSpaces(param_grid, model_name)
        model ← CreateModelInstance(model_name)
        bayes_search ←
BayesSearchCV(model, search_spaces, cv, scoring = 'r2')
        bayes_search.fit(X_train, y_train)
        bayes_model ← bayes_search.best_estimator_
        bayes_param ← bayes_search.best_params_
    end if
        best_models[model_name] ← best_model, best_params[model_name] ←
        best_param
  end for
  return best_models, best_params
end function
function ConvertToSearchSpaces(param_grid, model_name)
    search_spaces ← ∅
    for (param_name, param_values) ∈ param_grid do
        if all values are integers then
            search_spaces[param_name] ←
                Integer(min(param_values), max(param_values))
        else if all values are floats then
            search_spaces[param_name] ←
                Real(min(param_values), max(param_values), prior)
        else
            search_spaces[param_name] ← Categorical(param_values)
        end if
    end for
    Apply model − specific constraints and parameter mappings
    return search_spaces
end function
```

Figure S1:Distribution of PFAS Compounds and Plant Species in source dataset

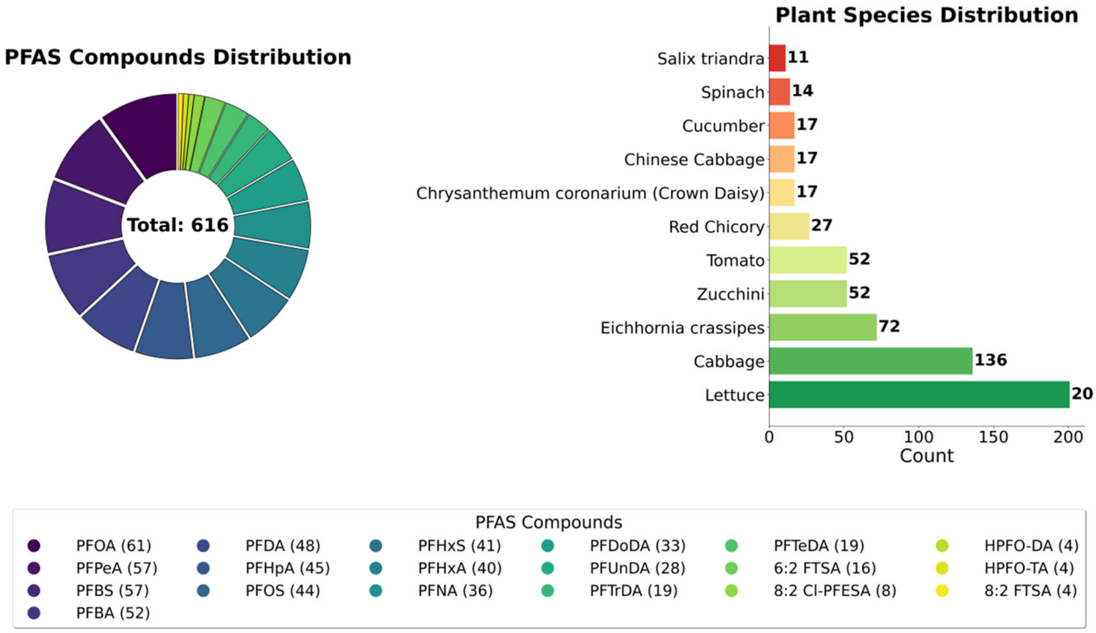

Figure S2:Correlation analysis

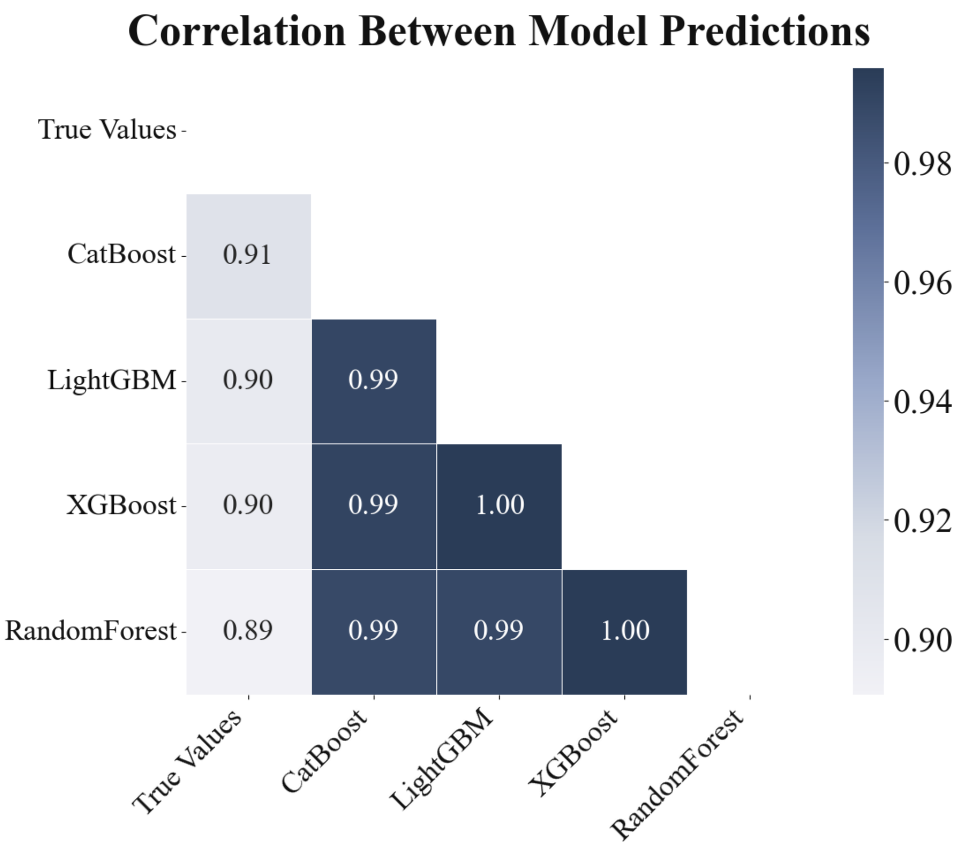

Table S1 Statistical summary of the numeric basic variables comprising the PFAS uptake and translocation compiled 616 point dataset.

Variables	pKa	Log K_{ow}	Exposure time (hrs)	RCF (L/g)
Count	616	616	616	613
Mean	0.104	3.610	1071.506	0.118
Std	1.371	2.065	655.814	0.307
Min	-1.640	1.350	24.000	0.000
25%	-0.800	2.163	720.000	0.005
50%	0.230	3.100	960.000	0.017
75%	0.540	4.150	1320.000	0.105
Max	3.150	8.760	2232.000	3.240

Table S2 Encoded variables

**Categories**	**Variables**	**Labels**
Plants	Cabbage	0
	Chinese Cabbage	1
	Chrysanthemum coronarium (Crown Daisy)	2
	Cucumber	3
	Eichhornia crassipes	4
	Lettuce	5
	Red Chicory	6
	Salix triandra	7
	Spinach	8
	Tomato	9
	Zucchini	10
Functional Groups	Carboxylate	0
	Sulfonate	1

Table S3 Key differences between the four models.

Models	Key Mechanism	Differences
CatBoost	Solving the Prediction Bias Problem by Ordered Boosting for Automatic Combination and Processing of Category Features Using Ranking-Based Statistical Coding of Objects	No additional preprocessing required for category feature processing; more stable training and better generalization; excellent performance with default parameters; slower numerical feature processing
XGBoost	Approximating the loss function using a second-order Taylor expansion, controlling the model complexity through regularization terms, and speeding up the training process using pre-ordered feature storage and chunked computation	First highly engineered and optimized GBDT implementation; Supports parallel computation and external memory processing; Uses Exact greedy algorithm to find optimal splitting point; High memory consumption
LightGBM	A histogram-based splitting algorithm combining GOSS sample sampling and EFB feature bundling to reduce the computational effort, using a Leaf-wise growth strategy to extend the tree structure	Training several times faster than XGBoost; lower memory consumption; better handling of high-dimensional sparse features; suitable for large-scale datasets; excellent distributed training performance
Random Forest	Construct multiple independent decision trees based on the bagging strategy, randomly select a subset of features to train each tree, and ultimately integrate the prediction results of all trees through voting or averaging	Parallel training, less hyperparameter tuning; insensitive to outliers; strong resistance to overfitting; no need for feature scaling; weak prediction bias of single tree; fast training speed

Table S4 Statistical comparison of target variable before and after augmentation.

Variables	Original training set	Post- augmentation training set	Relative change (%)
Count	462	4338	+839.0%
Mean	-3.872	-3.961	-2.3%
Std	1.780	1.835	+3.1%
Min	-6.645	-6.648	-0.04%
Medium	-4.136	-4.439	-7.3%
Max	-0.528	-0.526	+0.4%
Range	6.118	6.121	+0.05%

Formula S1 Multi-layer feature transfer equation construction (MFTEC) in original data.

logRCF=0.04254428×log(pKa) + 0.04977005×log(Molecular Weight (g/mol)) + 0.05100166×log(log Kow) + -0.05268060×FpDensityMorgan3 + 0.05375830×log(log_MW) + -0.06235329×pKa×exposure_sqrt + -0.06482233×Plant_Species_Encoded + 0.07377797×mol_size_effect + -0.07969414×log_MW^2 ×Plant_Species_Encoded + -0.09265232×log Kow^2 ×exposure_sqrt + 0.13646529 × Molecular Weight (g/mol) + 0.15512869 × Molecular Weight (g/mol) × Plant_Species_Encoded + 0.18378528 × log_MW ^{2} × exposure_sqrt + -0.18873064 × mol_transport + 0.23983266 × Molecular Weight (g/mol) × exposure_sqrt + 0.25636163 × water_solubility_index + 0.26192237 × sqrt(|Molecular Weight (g/mol)|) + -0.33833601 × Exposure time (hrs) + 0.36740736×equilibrium_factor + -0.39556891×pKa + -1.80322126

Formula S2 MFTEC in augmented data.

logRCF=0.41196423×exposure_log + 0.44056973×tanh(pKa) + 0.44170885×log_MW^2 ×exposure_sqrt + 0.48298396×exposure_sqrt + 0.51742501×water_solubility_index + -0.54493169×pKa + -0.56776039×absorption_kinetics + 0.66779385×exp(-|Molecular Weight (g/mol)|) + 0.66841154×log Kow + 0.70716139×tanh(log_MW) + -0.75968611× Exposure time (hrs) + 0.76969860×log_MW + 0.85843140×exp(-|log Kow|) + -0.88639760×mol_transport + -1.02150048×log_MW×exposure_sqrt + 1.04083479×tanh(Molecular Weight (g/mol)) + -1.06447252×tanh(log Kow) + -1.13227334×sqrt(|log_MW|) + 1.46590029×Molecular Weight (g/mol)×exposure_sqrt + -1.49103451×exp(-|log_MW|) + -2.72899179

Formula S3 High-dimensional sparse interaction equation (HSIE) in original data.

logRCF=0.14356514× Exposure time (hrs)*Plant_Species_Encoded*mol_size_effect + 0.14952537× Exposure time (hrs)*Plant_Species_Encoded*mw_medium + -0.16815897×Plant_Species_Encoded^2*absorption_kinetics + -0.16836622×pKa*Functional_Group_Encoded*exposure_log + -0.17111421×pKa* Exposure time (hrs)*Functional_Group_Encoded + 0.17278230× Exposure time (hrs)*Plant_Species_Encoded*mol_transport + 0.18005496×Plant_Species_Encoded*exposure_sqrt*equilibrium_factor + 0.18128138×Plant_Species_Encoded*Functional_Group_Encoded*exposure_log + 0.20204629× Exposure time (hrs)*Plant_Species_Encoded*equilibrium_factor + -0.20496423×sin(Plant_Species_Encoded) + -0.20728180× Exposure time (hrs)^2 + -0.21854269×Plant_Species_Encoded*equilibrium_factor + 0.22507474×Plant_Species_Encoded*mw_medium*fluorination_degree + 0.22786683×exposure_sqrt*mw_small*absorption_kinetics + 0.24167765×mw_medium^2*equilibrium_factor + -0.25567775×Molecular Weight (g/mol)* Exposure time (hrs)*Plant_Species_Encoded + 0.37926916× Exposure time (hrs)^2*Plant_Species_Encoded + 0.41806656×Plant_Species_Encoded^2*equilibrium_factor + 0.44177072×Molecular Weight (g/mol)/log Kow + 0.56697154× Exposure time (hrs)*Plant_Species_Encoded^2 + -3.85314425

Formula S4 High-dimensional sparse interaction equation (HSIE) in augmented data.

logRCF=-0.19794653× Exposure time (hrs)*exposure_sqrt*estate_polarity_ratio + -0.19860061×log Kow*Plant_Species_Encoded^2 + 0.21394305× Exposure time (hrs)*Plant_Species_Encoded*EState_VSA7 + -0.21688295×pKa* Exposure time (hrs) + -0.22169398×Functional_Group_Encoded*FCH_ratio*absorption_kinetics + 0.22215920×

Exposure time (hrs)*Plant_Species_Encoded*log_MW + 0.22327645× Exposure time (hrs)*Plant_Species_Encoded*equilibrium_factor + 0.22516322× mw_large^2*equilibrium_factor + 0.23926015×pKa*FpDensityMorgan3*mw_large + 0.25971404× Exposure time (hrs)*Plant_Species_Encoded*estate_polarity_ratio + -0.26724722×log Kow* Exposure time (hrs)*exposure_sqrt + 0.27916912× Plant_Species_Encoded^2*exposure_sqrt + -0.28560641×Molecular Weight (g/mol)* Exposure time (hrs)*Plant_Species_Encoded + -0.32729475×Molecular Weight (g/mol)*Plant_Species_Encoded^2 + 0.35329579×Molecular Weight (g/mol)/Functional_Group_Encoded + 0.37873610× Exposure time (hrs)*Plant_Species_Encoded*absorption_kinetics + -0.39896266× Plant_Species_Encoded*exposure_rate*equilibrium_factor + 1.07726026× Exposure time (hrs)*exposure_sqrt + -1.17696497× Exposure time (hrs)^2 + 1.37921918×Molecular Weight (g/mol) + -3.22808469

References.

Montal, M., Mueller, P., 1972. Formation of bimolecular membranes from lipid monolayers and a study of their electrical properties. Proceedings of the National Academy of Sciences of the United States of America. 69, 3561-6.

Potts, D. S., et al., 2021. Influence of solvent structure and hydrogen bonding on catalysis at solid-liquid interfaces. Chemical Society Reviews. 50, 12308-12337.

Appendix S1 Main python code.

```
plt.close('all')
shap.initjs()
try:
        ray.shutdown()
except:
        pass
try:
        ray.init(ignore_reinit_error=True)
except Exception as e:
        try:
                ray.init(local_mode=True, ignore_reinit_error=True)
        except Exception as e:
                raise e
warnings.filterwarnings('ignore')
num_cores = multiprocessing.cpu_count()
print(f"CPU: {num_cores}")
pfas_smiles = {
        'PFBA': 'C(=O)(C(C(C(F)(F)F)(F)F)(F)F)O',
        'PFPeA': 'C(=O)(C(C(C(C(F)(F)F)(F)F)(F)F)(F)F)O',
        'PFHxA': 'C(=O)(C(C(C(C(C(F)(F)F)(F)F)(F)F)(F)F)(F)F)O',
        'PFHpA': 'C(=O)(C(C(C(C(C(C(F)(F)F)(F)F)(F)F)(F)F)(F)F)(F)F)O',
        'PFOA': 'C(=O)(C(C(C(C(C(C(C(F)(F)F)(F)F)(F)F)(F)F)(F)F)(F)F)(F)F)O',
        'PFNA': ' C(=O)(C(C(C(C(C(C(C(C(F)(F)F)(F)F)(F)F)(F)F)(F)F)(F)F)(F)F)(F)F)O',
        'PFDA': 'C(=O)(C(C(C(C(C(C(C(C(C(F)(F)F)(F)F)(F)F)(F)F)(F)F)(F)F)(F)F)(F)F)(F)F)O',
        'PFBS': ' C(C(C(F)(F)S(=O)(=O)O)(F)F)(C(F)(F)F)(F)F',
        'PFHxS': 'C(C(C(C(F)(F)S(=O)(=O)O)(F)F)(F)F)(C(C(F)(F)F)(F)F)(F)F',
        'PFDoDA':
'C(=O)(C(C(C(C(C(C(C(C(C(C(C(F)(F)F)(F)F)(F)F)(F)F)(F)F)(F)F)(F)F)(F)F)(F)F)(F)F)(F)F)O',
        'PFOS': 'C(C(C(C(C(F)(F)S(=O)(=O)O)(F)F)(F)F)(F)F)(C(C(C(F)(F)F)(F)F)(F)F)(F)F',
        'PFTrDA':
'C(=O)(C(C(C(C(C(C(C(C(C(C(C(C(F)(F)F)(F)F)(F)F)(F)F)(F)F)(F)F)(F)F)(F)F)(F)F)(F)F)(F)F)(F)F)O',
        'PFTeDA':
'C(=O)(C(C(C(C(C(C(C(C(C(C(C(C(C(F)(F)F)(F)F)(F)F)(F)F)(F)F)(F)F)(F)F)(F)F)(F)F)(F)F)(F)F)(F)F)(F)F)
O',
        'PFUnDA': 'C(=O)(C(C(C(C(C(C(C(C(C(C(F)(F)F)(F)F)(F)F)(F)F)(F)F)(F)F)(F)F)(F)F)(F)F)(F)F)O',
        'HPFO-DA': 'C(=O)(C(C(F)(F)F)(OC(C(C(F)(F)F)(F)F)(F)F)F)O',
        'HPFO-TA': 'C(=O)(C(C(F)(F)F)(OC(C(C(F)(F)F)(OC(C(C(F)(F)F)(F)F)(F)F)F)(F)F)F)O',
        '6:2 FTSA': 'C(CS(=O)(=O)O)C(C(C(C(C(C(F)(F)F)(F)F)(F)F)(F)F)(F)F)(F)F',
        '8:2 FTSA': 'C(CS(=O)(=O)O)C(C(C(C(C(C(C(C(F)(F)F)(F)F)(F)F)(F)F)(F)F)(F)F)(F)F)(F)F',
        '8:2                                                                Cl-PFESA':
'C(C(C(C(C(F)(F)Cl)(F)F)(F)F)(F)F)(C(C(C(OC(C(F)(F)S(=O)(=O)O)(F)F)(F)F)(F)F)(F)F)(F)F'
}
```

```
@ray.remote
def get_pubchem_cid(compound_name):
    base_url = "https://pubchem.ncbi.nlm.nih.gov/rest/pug"
    search_url = f"{base_url}/compound/name/{compound_name}/cids/JSON"
    try:
        response = requests.get(search_url)
        if response.status_code == 200:
            data = response.json()
            if 'IdentifierList' in data and 'CID' in data['IdentifierList']:
                return str(data['IdentifierList']['CID'][0])
    except Exception as e:
        print(f"obtain CID error {compound_name}: {str(e)}")
    return None
@ray.remote
def get_pubchem_properties(cid):
    if cid is None:
        return {}
    base_url = "https://pubchem.ncbi.nlm.nih.gov/rest/pug"
    properties = [
        "MolecularWeight", "XLogP", "ExactMass", "MonoisotopicMass", "TPSA",
        "Complexity", "Charge", "HBondDonorCount", "HBondAcceptorCount",
        "RotatableBondCount", "HeavyAtomCount", "IsotopeAtomCount",
        "AtomStereoCount", "DefinedAtomStereoCount", "UndefinedAtomStereoCount",
        "BondStereoCount", "DefinedBondStereoCount", "UndefinedBondStereoCount",
        "CovalentUnitCount"
    ]
    prop_url = f"{base_url}/compound/cid/{cid}/property/{','.join(properties)}/JSON"
    try:
        response = requests.get(prop_url)
        if response.status_code == 200:
            data = response.json()
            if 'PropertyTable' in data and 'Properties' in data['PropertyTable']:
                return data['PropertyTable']['Properties'][0]
            return {}
    except Exception as e:
        print(f"obtain properties error {cid}: {str(e)}")
    return {}
@ray.remote
def calculate_rdkit_descriptors(mol):
    if mol is None:
        return None
    desc_dict = {}
    desc_dict.update({
        'MolWeight': Descriptors.ExactMolWt(mol),
```

```
        'LogP': Descriptors.MolLogP(mol),
        'HBA': Descriptors.NumHAcceptors(mol),
        'HBD': Descriptors.NumHDonors(mol),
        'TPSA': Descriptors.TPSA(mol),
        'RotableBonds': Descriptors.NumRotatableBonds(mol),
        'AromaticRings': Descriptors.NumAromaticRings(mol),
        'Rings': Descriptors.RingCount(mol),
        'PolarSurfaceArea': Descriptors.TPSA(mol),
})

desc_dict.update({
        'CrippenMR': Crippen.MolMR(mol),
        'CrippenLogP': Crippen.MolLogP(mol),
})

desc_dict.update({
        'BertzCT': BertzCT(mol),
        'Chi0n': rdMolDescriptors.CalcChi0n(mol),
        'Chi0v': rdMolDescriptors.CalcChi0v(mol),
        'Chi1n': rdMolDescriptors.CalcChi1n(mol),
        'Chi1v': rdMolDescriptors.CalcChi1v(mol),
        'Chi2n': rdMolDescriptors.CalcChi2n(mol),
        'Chi2v': rdMolDescriptors.CalcChi2v(mol),
        'Chi3n': rdMolDescriptors.CalcChi3n(mol),
        'Chi3v': rdMolDescriptors.CalcChi3v(mol),
        'Chi4n': rdMolDescriptors.CalcChi4n(mol),
        'Chi4v': rdMolDescriptors.CalcChi4v(mol),
        'HallKierAlpha': rdMolDescriptors.CalcHallKierAlpha(mol),
})

desc_dict.update({
        f'EState_VSA{i}': v for i, v in enumerate(EState_VSA.EState_VSA_(mol))
})
desc_dict.update({
        'nF': len([atom for atom in mol.GetAtoms() if atom.GetSymbol() == 'F']),
        'nC': len([atom for atom in mol.GetAtoms() if atom.GetSymbol() == 'C']),
        'nO': len([atom for atom in mol.GetAtoms() if atom.GetSymbol() == 'O']),
        'nS': len([atom for atom in mol.GetAtoms() if atom.GetSymbol() == 'S']),
        'nN': len([atom for atom in mol.GetAtoms() if atom.GetSymbol() == 'N']),
        'nP': len([atom for atom in mol.GetAtoms() if atom.GetSymbol() == 'P']),
        'NumAtoms': mol.GetNumAtoms(),
        'NumBonds': mol.GetNumBonds(),
        'NumRotatableBonds': rdMolDescriptors.CalcNumRotatableBonds(mol),
        'NumHAcceptors': rdMolDescriptors.CalcNumHBA(mol),
```

```
            'NumHDonors': rdMolDescriptors.CalcNumHBD(mol),
            'NumHeteroatoms': rdMolDescriptors.CalcNumHeteroatoms(mol),
            'NumAmideBonds': rdMolDescriptors.CalcNumAmideBonds(mol),
        })
        desc_dict.update({
            'QED': QED.default(mol),
            'FractionCSP3': rdMolDescriptors.CalcFractionCSP3(mol),
            'HeavyAtomCount': Descriptors.HeavyAtomCount(mol),
            'NHOHCount': Descriptors.NHOHCount(mol),
            'NOCount': Descriptors.NOCount(mol),
            'MolMR': Descriptors.MolMR(mol),
            'LabuteASA': rdMolDescriptors.CalcLabuteASA(mol),
            'Kappa1': rdMolDescriptors.CalcKappa1(mol),
            'Kappa2': rdMolDescriptors.CalcKappa2(mol),
            'Kappa3': rdMolDescriptors.CalcKappa3(mol),
            'F_fraction': len([atom for atom in mol.GetAtoms() if atom.GetSymbol() == 'F']) /
mol.GetNumAtoms(),
            'C_F_ratio': len([atom for atom in mol.GetAtoms() if atom.GetSymbol() == 'C']) /
                         (len([atom for atom in mol.GetAtoms() if atom.GetSymbol() == 'F']) or
1),
            'CF3_count': len(mol.GetSubstructMatches(Chem.MolFromSmarts('FC(F)(F)'))),
            'CF2_count': len(mol.GetSubstructMatches(Chem.MolFromSmarts('FC(F)'))),
            'SO3_count':
len(mol.GetSubstructMatches(Chem.MolFromSmarts('S(=O)(=O)[O-,OH]'))),
            'COOH_count':
len(mol.GetSubstructMatches(Chem.MolFromSmarts('C(=O)[O-,OH]'))),
            'charged_groups': len(mol.GetSubstructMatches(Chem.MolFromSmarts('[+,-]'))),
            'BalabanJ': Descriptors.BalabanJ(mol),
            'MolWt': Descriptors.MolWt(mol),
            'NumValenceElectrons': Descriptors.NumValenceElectrons(mol),
            'FCH_ratio': len([atom for atom in mol.GetAtoms() if atom.GetSymbol() == 'F']) /
                         (mol.GetNumAtoms() - len([atom for atom in mol.GetAtoms() if
atom.GetSymbol() == 'F']) or 1),
        })
        fp1 = rdMolDescriptors.GetMorganFingerprintAsBitVect(mol, 1, nBits=1024)
        fp2 = rdMolDescriptors.GetMorganFingerprintAsBitVect(mol, 2, nBits=1024)
        fp3 = rdMolDescriptors.GetMorganFingerprintAsBitVect(mol, 3, nBits=1024)
        desc_dict.update({
            'FpDensityMorgan1': sum(fp1.GetOnBits()) / 1024.0,
            'FpDensityMorgan2': sum(fp2.GetOnBits()) / 1024.0,
            'FpDensityMorgan3': sum(fp3.GetOnBits()) / 1024.0,
        })
        slogp_vsa = rdMolDescriptors.SlogP_VSA_(mol)
        desc_dict.update({
```

```
            'SlogP_VSA1': slogp_vsa[0],
            'SlogP_VSA2': slogp_vsa[1],
            'SlogP_VSA3': slogp_vsa[2],
        })
        smr_vsa = rdMolDescriptors.SMR_VSA_(mol)
        desc_dict.update({
            'SMR_VSA1': smr_vsa[0],
            'SMR_VSA2': smr_vsa[1],
            'SMR_VSA3': smr_vsa[2],
        })

        peoe_vsa = rdMolDescriptors.PEOE_VSA_(mol)
        desc_dict.update({
            'PEOE_VSA1': peoe_vsa[0],
            'PEOE_VSA2': peoe_vsa[1],
            'PEOE_VSA3': peoe_vsa[2],
        })
        carbon_chain = mol.GetSubstructMatches(Chem.MolFromSmarts('C-C'))
        desc_dict['CarbonChainLength'] = len(carbon_chain) + 1 if carbon_chain else 0
        cf2_chain = mol.GetSubstructMatches(Chem.MolFromSmarts('FC(F)C(F)(F)'))
        desc_dict['CF2ChainLength'] = len(cf2_chain) + 1 if cf2_chain else 0
        return desc_dict
@ray.remote
def process_feature_chunk(X_chunk, y_chunk):
        min_samples = 5
        if len(X_chunk) < min_samples:
            return  pd.Series(0,  index=X_chunk.columns),  pd.Series(0,  index=X_chunk.columns),
pd.Series(0, index=X_chunk.columns)
        f_selector = SelectKBest(score_func=f_regression, k='all')
        f_selector.fit(X_chunk, y_chunk)
        f_scores = pd.Series(f_selector.scores_, index=X_chunk.columns)
        n_neighbors = min(3, len(X_chunk) - 1)
        try:
            mi_selector = SelectKBest(
                score_func=lambda X, y: mutual_info_regression(
                    X, y, n_neighbors=n_neighbors
                ),
                k='all'
            )
            mi_selector.fit(X_chunk, y_chunk)
            mi_scores = pd.Series(mi_selector.scores_, index=X_chunk.columns)
        except ValueError:
            mi_scores = pd.Series(0, index=X_chunk.columns)
        dcor_scores = pd.Series(0, index=X_chunk.columns)
```

```
    try:
        from dcor import distance_correlation
        for col in X_chunk.columns:
            dcor_scores[col] = distance_correlation(X_chunk[col].values.reshape(-1, 1), y_chunk.values.reshape(-1, 1))
            if np.isnan(dcor_scores[col]):
                dcor_scores[col] = 0
    except:
        for col in X_chunk.columns:
            from scipy.stats import spearmanr
            corr, _ = spearmanr(X_chunk[col].values, y_chunk.values)
            dcor_scores[col] = abs(corr) if not np.isnan(corr) else 0
    return f_scores, mi_scores, dcor_scores
def select_features_parallel(X, y, threshold=0.15, n_chunks=4):
    min_samples_per_chunk = 5
    n_chunks = min(n_chunks, len(X) // min_samples_per_chunk)
    if n_chunks < 1:
        n_chunks = 1
    chunk_size = len(X) // n_chunks
    chunks = []
    for i in range(0, len(X), chunk_size):
        end_idx = min(i + chunk_size, len(X))
        if end_idx - i >= min_samples_per_chunk:
            chunks.append((X.iloc[i:end_idx], y.iloc[i:end_idx]))
    if not chunks:
        chunks = [(X, y)]
    futures = [process_feature_chunk.remote(X_chunk, y_chunk)
               for X_chunk, y_chunk in chunks]
    results = ray.get(futures)
    f_scores_total = sum(result[0] for result in results) / len(results)
    mi_scores_total = sum(result[1] for result in results) / len(results)
    dcor_scores_total = sum(result[2] for result in results) / len(results)
    def normalize_scores(scores):
        max_score = scores.max()
        if max_score > 0:
            return scores / max_score
        return scores
    f_scores_norm = normalize_scores(f_scores_total)
    mi_scores_norm = normalize_scores(mi_scores_total)
    dcor_scores_norm = normalize_scores(dcor_scores_total)
    combined_scores = (0.4 * f_scores_norm + 0.3 * mi_scores_norm + 0.3 * dcor_scores_norm)
    corr_matrix = X.select_dtypes(include=[np.number]).corr().abs()
    try:
```

```
        from sklearn.utils import resample
        stability_scores = pd.Series(0, index=X.columns)
        n_bootstraps = 10
        for _ in range(n_bootstraps):
            X_boot, y_boot = resample(X, y, random_state=np.random.randint(0, 1000))
            f_selector = SelectKBest(score_func=f_regression, k='all')
            f_selector.fit(X_boot, y_boot)
            boot_scores = pd.Series(f_selector.scores_, index=X.columns)
            boot_scores = normalize_scores(boot_scores)
            stability_scores += boot_scores
        stability_scores = stability_scores / n_bootstraps
        combined_scores = 0.6 * combined_scores + 0.4 * stability_scores
    except:
        pass
    try:
        from statsmodels.stats.outliers_influence import variance_inflation_factor
        potential_features = combined_scores.nlargest(min(50,
len(combined_scores))).index.tolist()
        X_subset = X[potential_features]
        vif_data = pd.DataFrame()
        vif_data["feature"] = X_subset.columns
        vif_data["VIF"] = [variance_inflation_factor(X_subset.values, i) for i in
range(X_subset.shape[1])]
        vif_penalty = pd.Series(0, index=X.columns)
        for feature, vif in zip(vif_data["feature"], vif_data["VIF"]):
            if vif > 10:
                vif_penalty[feature] = 0.3
            elif vif > 5:
                vif_penalty[feature] = 0.15
        combined_scores = combined_scores * (1 - vif_penalty)
    except:
        try:
            collinearity_penalty = pd.Series(0, index=X.columns)
            high_collinearity_threshold = 0.7

            numeric_cols = X.select_dtypes(include=[np.number]).columns
            for i, col1 in enumerate(numeric_cols):
                collinearity_count = 0
                for col2 in numeric_cols[i+1:]:
                    if corr_matrix.loc[col1, col2] > high_collinearity_threshold:
                        collinearity_count += 1
                        if combined_scores[col1] < combined_scores[col2]:
                            collinearity_penalty[col1] += 0.2 * corr_matrix.loc[col1, col2]
                        else:
```

```
                        collinearity_penalty[col2] += 0.2 * corr_matrix.loc[col1, col2]

                if collinearity_count > 3:
                    collinearity_penalty[col1] += 0.1 * collinearity_count

            combined_scores = combined_scores * (1 - collinearity_penalty)
        except:
            pass
    try:
        from minepy import MINE
        mic_scores = pd.Series(index=X.columns)
        mine = MINE(alpha=0.6, c=15)

        for col in X.columns:
            mine.compute_score(X[col].values, y.values)
            mic_scores[col] = mine.mic()
            if np.isnan(mic_scores[col]):
                mic_scores[col] = 0
        mic_scores = normalize_scores(mic_scores)
        combined_scores = 0.75 * combined_scores + 0.25 * mic_scores
    except:
        try:
            from scipy.stats import kendalltau
            kendall_scores = pd.Series(index=X.columns)
            for col in X.columns:
                tau, p_value = kendalltau(X[col], y)
                kendall_scores[col] = abs(tau) if not np.isnan(tau) else 0
                if p_value > 0.05:
                    kendall_scores[col] *= 0.5
            kendall_scores = normalize_scores(kendall_scores)
            combined_scores = 0.85 * combined_scores + 0.15 * kendall_scores
        except:
            pass
    try:
        from skrebate import ReliefF
        max_samples = min(5000, len(X))
        if len(X) > max_samples:
            X_sample, y_sample = resample(X, y, n_samples=max_samples, random_state=42)
        else:
            X_sample, y_sample = X, y
        relief = ReliefF(n_features_to_select=len(X.columns), n_neighbors=min(10, len(X_sample)-1))
        relief.fit(X_sample.values, y_sample.values)
```

```
            relief_scores = pd.Series(relief.feature_importances_, index=X.columns)
            relief_scores = normalize_scores(relief_scores)
            combined_scores = 0.7 * combined_scores + 0.3 * relief_scores
        except:
            pass
        try:
            from sklearn.preprocessing import RobustScaler
            scaler = RobustScaler()
            X_scaled = pd.DataFrame(scaler.fit_transform(X), columns=X.columns)
            from scipy.stats import median_abs_deviation
            variance_scores = pd.Series(index=X.columns)
            for col in X.columns:
                variance_scores[col] = median_abs_deviation(X_scaled[col],
nan_policy='omit')**2
            variance_scores = normalize_scores(variance_scores)
            low_var_threshold = 0.05
            for col in X.columns:
                if variance_scores[col] < low_var_threshold:
                    combined_scores[col] *= (0.5 + 0.5 * variance_scores[col] /
low_var_threshold)
        except:
            try:
                variance_scores = pd.Series(index=X.columns)
                for col in X.columns:
                    variance_scores[col] = X[col].var()
                variance_scores = normalize_scores(variance_scores)
                low_var_threshold = 0.05
                for col in X.columns:
                    if variance_scores[col] < low_var_threshold:
                        combined_scores[col] *= (0.5 + 0.5 * variance_scores[col] /
low_var_threshold)
            except:
                pass
        target_selection_ratio = 0.4
        percentile_threshold = max(combined_scores.quantile(1 - target_selection_ratio),
threshold * combined_scores.max())
        selected_features = combined_scores[combined_scores >
percentile_threshold].index.tolist()
        min_features = max(5, int(len(X.columns) * 0.1))
        max_features = int(len(X.columns) * 0.5)
        if len(selected_features) < min_features:
            k = min_features
            selected_features = combined_scores.nlargest(k).index.tolist()
        elif len(selected_features) > max_features:
```

```
            k = max_features
            selected_features = combined_scores.nlargest(k).index.tolist()
        return selected_features
@ray.remote
def process_feature_engineering_chunk(X_chunk, exclude_columns):
    X = X_chunk.copy()
    if ' Exposure time    (hrs)' in X.columns:
        time = X[' Exposure time    (hrs)']
        X['exposure_log'] = np.log1p(time)
        X['exposure_sqrt'] = np.sqrt(time)
        X['exposure_rate'] = 1 - np.exp(-0.05 * time)
    all_plant_features = []
    if all(col in X.columns for col in ['Molecular Weight (g/mol)', 'log Kow']):
        mw = X['Molecular Weight (g/mol)']
        kow = X['log Kow']
        X['mol_transport'] = kow / np.sqrt(mw)
        X['mol_size_effect'] = np.log1p(mw) / (1 + np.abs(kow))
        X['mw_small'] = np.where(mw <= 300, 1, 0)
        X['mw_medium'] = np.where((mw > 300) & (mw <= 500), 1, 0)
        X['mw_large'] = np.where(mw > 500, 1, 0)
        X['membrane_permeability_logkow'] = kow / np.log10(mw)
        X['log_MW'] = np.log1p(mw)
    if all(col in X.columns for col in ['nC', 'nF']):
        X['CF_interaction'] = X['nF'] * X['nC']
        X['fc_ratio'] = X['nF'] / (X['nC'] + 1e-6)
        if 'XLogP' in X.columns:
            X['fc_ratio_logp'] = X['fc_ratio'] * X['XLogP']
        if 'NumAtoms' in X.columns:
            X['fluorination_degree'] = X['nF'] / (X['NumAtoms'] + 1e-6)
    if all(col in X.columns for col in ['TPSA', 'HBondDonorCount', 'HBondAcceptorCount']):
        X['polarity_index'] = X['TPSA'] * (X['HBondDonorCount'] + X['HBondAcceptorCount'])
        X['hbond_balance'] = X['HBondDonorCount'] / (X['HBondAcceptorCount'] + 1e-6)
        if 'XLogP' in X.columns:
            X['polar_lipophilic_ratio'] = X['TPSA'] / (abs(X['XLogP']) + 1)
    connectivity_cols = ['Chi0n', 'Chi1n', 'Chi2n', 'Chi3n', 'Chi4n']
    if all(col in X.columns for col in connectivity_cols):
        X['connectivity_complexity'] = (X['Chi0n'] + 0.7*X['Chi1n'] + 0.5*X['Chi2n'] +
                                        0.3*X['Chi3n'] + 0.1*X['Chi4n'])
        X['branching_index'] = X['Chi3n'] / (X['Chi1n'] + 1e-6)
    if all(col in X.columns for col in ['CrippenLogP', 'XLogP']):
        X['logp_consensus'] = (X['CrippenLogP'] + X['XLogP']) / 2
        X['logp_deviation'] = abs(X['CrippenLogP'] - X['XLogP'])
        if 'CrippenMR' in X.columns:
            X['crippen_polarity_index'] = X['CrippenMR'] / (abs(X['CrippenLogP']) + 1)
```

```
    estate_cols = [col for col in X.columns if col.startswith('EState_VSA')]
    if estate_cols:
        X['estate_range'] = X[estate_cols].max(axis=1) - X[estate_cols].min(axis=1)
        X['estate_mean'] = X[estate_cols].mean(axis=1)
        polar_estates = ['EState_VSA8', 'EState_VSA9', 'EState_VSA10']
        nonpolar_estates = ['EState_VSA1', 'EState_VSA2', 'EState_VSA3']
        if all(col in X.columns for col in polar_estates + nonpolar_estates):
            X['estate_polarity_ratio'] = (X[polar_estates].sum(axis=1) + 1e-6) / (X[nonpolar_estates].sum(axis=1) + 1e-6)
    if all(col in X.columns for col in ['AtomStereoCount', 'BondStereoCount', 'HeavyAtomCount']):
        X['stereo_complexity'] = (X['AtomStereoCount'] + X['BondStereoCount']) / (X['HeavyAtomCount'] + 1)
        if all(col in X.columns for col in ['HBondDonorCount', 'HBondAcceptorCount']):
            X['stereo_hbond_index'] = (X['AtomStereoCount'] + X['BondStereoCount']) * (X['HBondDonorCount'] + X['HBondAcceptorCount'])
    if ' Exposure time    (hrs)' in X.columns and 'log Kow' in X.columns:
        X['absorption_kinetics'] = (1 - np.exp(-0.005 * X[' Exposure time    (hrs)'])) * np.exp(-0.2 * X['log Kow'])
        X['equilibrium_factor'] = 1 - np.exp(-X[' Exposure time    (hrs)'] / (100 + 20 * X['log Kow']))
        if 'TPSA' in X.columns and 'Molecular Weight (g/mol)' in X.columns:
            X['water_solubility_index'] = X['TPSA'] / (X['Molecular Weight (g/mol)'] * (1 + 0.5 * X['log Kow']))
    for feature in all_plant_features:
        if feature not in X.columns:
            X[feature] = 0
    return X
def engineer_features_parallel(X, exclude_columns=[], n_chunks=4):
    chunk_size = len(X) // n_chunks
    chunks = [X.iloc[i:i+chunk_size] for i in range(0, len(X), chunk_size)]
    futures = [process_feature_engineering_chunk.remote(chunk, exclude_columns)
               for chunk in chunks]
    results = ray.get(futures)
    return pd.concat(results, axis=0)
@ray.remote
def evaluate_model_fold(model_class, params, X_train, X_val, y_train, y_val):
    model = model_class(**params)
    model.fit(X_train, y_train)
    pred = model.predict(X_val)
    def safe_mape(y_true, y_pred):
        mask = y_true != 0
        return np.mean(np.abs((y_true[mask] - y_pred[mask]) / y_true[mask])) * 100 if mask.any() else 0
```

```
        metrics = {
            'r2': r2_score(y_val, pred),
            'rmse': np.sqrt(mean_squared_error(y_val, pred)),
            'mae': mean_absolute_error(y_val, pred),
            'explained_variance': explained_variance_score(y_val, pred),
            'max_error': max_error(y_val, pred),
            'mape': safe_mape(y_val.values, pred)
        }
        if hasattr(model, 'feature_importances_'):
            importances = model.feature_importances_
        else:
            importances = None
        return metrics, importances, pred
def evaluate_test_metrics(y_true, y_pred, transform_params=None):
        transformed_metrics = {
            'R² ': r2_score(y_true, y_pred),
            'RMSE': np.sqrt(mean_squared_error(y_true, y_pred)),
            'MAE': mean_absolute_error(y_true, y_pred),
            'Explained Variance': explained_variance_score(y_true, y_pred),
            'Max Error': max_error(y_true, y_pred)
        }
        metrics = transformed_metrics
        return metrics
def print_test_metrics(metrics_dict, model_name=""):
        print(f"\n{model_name} TEST EVALUATION")
        print("-" * 50)
        transformed_metrics = {k: v for k, v in metrics_dict.items() if not k.startswith('Original')}
        if transformed_metrics:
            for metric, value in transformed_metrics.items():
                print(f"{metric:<20} : {value:>10.4f}")
        original_metrics = {k: v for k, v in metrics_dict.items() if k.startswith('Original')}
        if original_metrics:
            print("\nORIGINAL INDICATORS")
            for metric, value in original_metrics.items():
                print(f"{metric[9:]:<20} : {value:>10.4f}")
        print("-" * 50)
def visualize_results(all_results):
        plt.figure(figsize=(20, 15))
        for idx, (target_name, results) in enumerate(all_results.items()):
            try:
                plt.subplot(3, 3, idx*3 + 1)
                if 'final_predictions' in results:
                    for model_name, pred in results['final_predictions'].items():
                        if model_name != 'Ensemble':
```

```
                    plt.scatter(results['y_test'], pred, alpha=0.5, label=model_name)
            plt.xlabel('TRUE')
            plt.ylabel('PREDICTION')
            plt.title(f'{target_name} COMPARE')
            plt.legend()
            plt.grid(True)
            min_val = min(min(results['y_test']))
            max_val = max(max(results['y_test']))
            plt.plot([min_val, max_val], [min_val, max_val], 'k--', label='Perfect Prediction')
            plt.subplot(3, 3, idx*3 + 2)
            for model_name, pred in results['final_predictions'].items():
                if model_name != 'Ensemble':
                    residuals = pred - results['y_test']
                    plt.scatter(pred, residuals, alpha=0.5, label=model_name)
            plt.axhline(y=0, color='r', linestyle='--')
            plt.xlabel('PREDICTIONS')
            plt.ylabel('RESIDUAL')
            plt.title(f'{target_name} RESIDUAL DISTRIBUTION')
            plt.grid(True)
            plt.legend()
            if 'feature_importance' in results and results['feature_importance']:
                plt.subplot(3, 3, idx*3 + 3)
                for model_name in ['XGBoost', 'RandomForest','LightGBM']:
                    if model_name in results['feature_importance']:
                        feature_imp = results['feature_importance'][model_name]
                        sorted_features = dict(sorted(feature_imp.items(), key=lambda x:
x[1], reverse=True)[:20])
                        plt.barh(range(len(sorted_features)),
list(sorted_features.values()), align='center')
                        plt.yticks(range(len(sorted_features)),
list(sorted_features.keys()))
                        plt.xlabel('IMPORTANCE')
                        plt.title(f'{target_name}  FEATURE  IMPORTANCE  ({model_name}
Top 20)')
                        break
        except Exception as e:
            print(f"{target_name} ERROR: {str(e)}")
            import traceback
            traceback.print_exc()
            continue
    plt.tight_layout()
    plt.show()
def visualize_features(X, y, target_name, save_path='feature_plots'):
    os.makedirs(save_path, exist_ok=True)
```

```
sns.set_style("whitegrid")
plt.rcParams.update({'figure.figsize': (30, 25),'font.size': 28,'font.family': 'serif','grid.linewidth': 0.5,'grid.alpha': 0.3,'axes.titlesize': 32,'axes.labelsize': 30,'xtick.labelsize': 28,'ytick.labelsize': 28})
model = RandomForestRegressor(n_estimators=100, random_state=42)
model.fit(X, y)
importance = pd.Series(model.feature_importances_, index=X.columns)
results = ray.get([process_feature_chunk.remote(X, y)])
f_scores_total = results[0][0]
mi_scores_total = results[0][1]
dcor_scores_total = results[0][2]
def normalize_scores(scores):
    max_score = scores.max()
    if max_score > 0:
        return scores / max_score
    return scores
f_scores_norm = normalize_scores(f_scores_total)
mi_scores_norm = normalize_scores(mi_scores_total)
dcor_scores_norm = normalize_scores(dcor_scores_total)
combined_scores = (0.4 * f_scores_norm + 0.3 * mi_scores_norm + 0.3 * dcor_scores_norm)
X_all = X.copy()
pubchem_cols = [col for col in X_all.columns if 'pubchem' in col]
for col in pubchem_cols:
    if X_all[col].isnull().any():
        X_all[col] = X_all[col].fillna(0)
    if X_all[col].std() == 0:
        X_all[col] = X_all[col] + np.random.normal(0, 1e-6, size=len(X_all[col]))
corr_matrix = X_all.corr()
corr_matrix.to_csv(f'{save_path}/{target_name}_correlation_matrix.csv')
if corr_matrix.isnull().any().any():
    corr_matrix = corr_matrix.fillna(0)
std_series = X_all.std()
valid_features = std_series[std_series > 0].index
corr_matrix_filtered = corr_matrix.loc[valid_features, valid_features]
corr_matrix_filtered = corr_matrix_filtered.fillna(0)
corr_matrix_filtered = corr_matrix_filtered.replace([np.inf, -np.inf], 0)
if corr_matrix_filtered.empty or not np.all(np.isfinite(corr_matrix_filtered.values)):
    print("Warning: Unable to create correlation matrix with valid values. Skipping hierarchical clustering.")
    plt.figure(figsize=(20, 16))
    sns.heatmap(corr_matrix.fillna(0).replace([np.inf, -np.inf], 0), cmap=sns.diverging_palette(220, 20, as_cmap=True, center="light"), center=0, vmin=-1, vmax=1, square=True)
```

```
            plt.title(f'Features Correlation Matrix - {target_name}', size=32, weight='bold')
            plt.tight_layout()
            plt.savefig(f'{save_path}/{target_name}_features_correlation_matrix.png',    dpi=300,
bbox_inches='tight')
            plt.close()
        else:
            try:
                from scipy.spatial.distance import squareform
                distance_matrix = 1 - np.abs(corr_matrix_filtered.values)
                distance_matrix = np.maximum(distance_matrix, 0)
                distance_matrix = (distance_matrix + distance_matrix.T) / 2
                np.fill_diagonal(distance_matrix, 0)
                if not np.all(np.isfinite(distance_matrix)):
                    distance_matrix  =  np.nan_to_num(distance_matrix,  nan=0.0,  posinf=0.0,
neginf=0.0)
                if np.any(distance_matrix < 0):
                    print("Warning: Negative distances detected, setting to zero")
                    distance_matrix = np.maximum(distance_matrix, 0)
                condensed_dist = squareform(distance_matrix)
                linkage = hierarchy.linkage(condensed_dist, method='ward')
                dendro = hierarchy.dendrogram(linkage, no_plot=True)
                reordered_idx = dendro['leaves']
                corr_matrix_clustered = corr_matrix_filtered.iloc[reordered_idx, reordered_idx]

corr_matrix_clustered.to_csv(f'{save_path}/{target_name}_clustered_correlation_matrix.csv')
                fig = plt.figure(figsize=(30, 25))
                gs = gridspec.GridSpec(10, 11)
                ax_heatmap = plt.subplot(gs[0:10, 0:9])
                palette = sns.diverging_palette(220, 20, as_cmap=True, center="light")
                heatmap                                                                    =
sns.heatmap(corr_matrix_clustered,ax=ax_heatmap,cmap=palette,center=0,vmin=-
1,vmax=1,square=True,annot=False,cbar_kws={'label':   'Correlation   Coefficient','orientation':
'vertical','pad':                                                              0.02,'fraction':
0.05},xticklabels=True,yticklabels=True,linewidths=0.5,linecolor='white')
                cbar = heatmap.collections[0].colorbar
                cbar.ax.tick_params(labelsize=28)
                cbar.ax.set_ylabel('Correlation     Coefficient',     fontsize=30,     rotation=270,
labelpad=20)

ax_heatmap.set_xticklabels(ax_heatmap.get_xticklabels(),rotation=45,ha='right',rotation_mod
e='anchor',fontsize=28)

ax_heatmap.set_yticklabels(ax_heatmap.get_yticklabels(),rotation=0,fontsize=28)
                ax_dendro_top = plt.subplot(gs[0:10, 10])
```

```
hierarchy.dendrogram(linkage,ax=ax_dendro_top,orientation='right',color_threshold=0.7,leaf_
font_size=28,no_labels=True)
            ax_dendro_top.set_xticks([])
            ax_dendro_top.set_yticks([])
            ax_dendro_top.spines['top'].set_visible(False)
            ax_dendro_top.spines['right'].set_visible(False)
            ax_dendro_top.spines['bottom'].set_visible(False)
            ax_dendro_top.spines['left'].set_visible(False)
            ax_dendro_right = plt.subplot(gs[0:10, 9:10])

hierarchy.dendrogram(linkage,ax=ax_dendro_right,orientation='right',color_threshold=0.7,leaf
_font_size=28,no_labels=True)
            ax_dendro_right.set_xticks([])
            ax_dendro_right.set_yticks([])
            ax_dendro_right.spines['top'].set_visible(False)
            ax_dendro_right.spines['right'].set_visible(False)
            ax_dendro_right.spines['bottom'].set_visible(False)
            ax_dendro_right.spines['left'].set_visible(False)
            plt.suptitle(f'Features Correlation Matrix - {target_name}\n(With Hierarchical
Clustering)',y=0.95,size=32,weight='bold')
            plt.tight_layout(rect=[0, 0.03, 1, 0.95])

plt.savefig(f'{save_path}/{target_name}_features_correlation_matrix_clustered.png',dpi=600,b
box_inches='tight',facecolor='white',edgecolor='none',format='png',metadata={'Creator':
'Matplotlib'})
            plt.close()
        except Exception as e:
            print(f"Error during hierarchical clustering: {e}")
            plt.figure(figsize=(20, 16))
            sns.heatmap(corr_matrix.fillna(0).replace([np.inf, -np.inf], 0),
cmap=sns.diverging_palette(220, 20, as_cmap=True, center="light"), center=0, vmin=-1,
vmax=1, square=True)
            plt.title(f'Features Correlation Matrix - {target_name}', size=32, weight='bold')
            plt.tight_layout()
            plt.savefig(f'{save_path}/{target_name}_features_correlation_matrix.png',
dpi=300, bbox_inches='tight')
            plt.close()
    sorted_scores = combined_scores.sort_values(ascending=False)
    sorted_scores_top20 = sorted_scores.head(20)
    plt.figure(figsize=(24, 24))
    ax = plt.subplot(111, polar=True)
    theta = np.linspace(0, 2*np.pi, len(sorted_scores_top20)+1)[:-1]
    width = 2*np.pi/len(sorted_scores_top20)
```

```
    values = sorted_scores_top20.values
    color_palette = plt.cm.tab20(np.linspace(0, 1, 20))
    bars = ax.bar(theta, values, width=width, bottom=0.0, alpha=0.8)
    for i, bar in enumerate(bars):
        bar.set_facecolor(color_palette[i])
        bar.set_edgecolor('white')
        bar.set_linewidth(0.5)
    max_value = max(values)
    yticks = [0.2, 0.4, 0.6, 0.8, 1.0]
    yticks = [y for y in yticks if y <= max_value]
    if max_value not in yticks:
        yticks.append(max_value)
    ax.set_yticks(yticks)
    ax.set_yticklabels([f"{y:.1f}" for y in yticks], fontsize=22)
    ax.spines['polar'].set_visible(False)
    ax.grid(color='lightgray', linestyle='--', alpha=0.7)
    plt.title(f'Top 20 Feature Importance Scores - {target_name}', fontsize=32, pad=50)
    legend_elements = []
    for i, feature in enumerate(sorted_scores_top20.index):
        legend_elements.append(plt.Line2D([0], [0], color=color_palette[i], lw=0, marker='o',
markersize=15, label=feature))
    plt.legend(handles=legend_elements, loc='center left', bbox_to_anchor=(1.1, 0.5),
fontsize=22, frameon=False)
    plt.tight_layout()
    plt.savefig(f'{save_path}/{target_name}_top20_feature_importance_scores.png',
dpi=300, bbox_inches='tight', facecolor='white')
    plt.close()
    feature_stats = pd.DataFrame({
        'mean': X.mean(),
        'std': X.std(),
        'min': X.min(),
        'max': X.max(),
        'null_count': X.isnull().sum(),
        'correlation_with_target': X.corrwith(y),
        'feature_importance': importance,
        'f_scores': f_scores_total,
        'mi_scores': mi_scores_total,
        'dcor_scores': dcor_scores_total,
        'f_scores_norm': f_scores_norm,
        'mi_scores_norm': mi_scores_norm,
        'dcor_scores_norm': dcor_scores_norm,
        'combined_score': combined_scores
    })
    feature_stats = feature_stats.sort_values('combined_score', ascending=False)
```

```
        feature_stats.to_csv(f'{save_path}/{target_name}_feature_stats.csv')
        plt.figure(figsize=(16, 10))
        subset_size = min(20, len(combined_scores))
        top_features = combined_scores.nlargest(subset_size).index
        top_corr = corr_matrix.loc[top_features, top_features]
        top_corr = top_corr.fillna(0).replace([np.inf, -np.inf], 0)
        top_corr.to_csv(f'{save_path}/{target_name}_top{subset_size}_correlation_matrix.csv')
        mask = np.triu(np.ones_like(top_corr, dtype=bool))
        cmap = sns.color_palette("PuBuGn", as_cmap=True)
        sns.heatmap(top_corr, mask=mask, cmap=cmap, vmax=1, vmin=-1, center=0,
square=True, linewidths=.5, cbar_kws={"shrink": .5}, annot=True, fmt=".2f", annot_kws={"size":
28})
        plt.title(f'Top {subset_size} Features Correlation Heatmap - {target_name}', fontsize=32)
        plt.tight_layout()
        plt.savefig(f'{save_path}/{target_name}_top{subset_size}_correlation_heatmap.png',
dpi=300, bbox_inches='tight')
        plt.close()
def create_shap_visualizations(base_path, target_name='logRCF'):
        try:
                shap_dir = os.path.join(base_path, 'shap_analysis', target_name)
                if not os.path.exists(shap_dir):
                        print(f"no shap: {shap_dir}")
                        return
                viz_dir = os.path.join(base_path, 'shap_visualizations')
                os.makedirs(viz_dir, exist_ok=True)
                model_dirs = [d for d in os.listdir(shap_dir) if os.path.isdir(os.path.join(shap_dir, d))]
                for model_name in model_dirs:
                        model_dir = os.path.join(shap_dir, model_name)
                        model_viz_dir = os.path.join(viz_dir, model_name)
                        os.makedirs(model_viz_dir, exist_ok=True)
                        shap_values_path = os.path.join(model_dir, 'shap_values.npy')
                        feature_names_path = os.path.join(model_dir, 'feature_names.txt')
                        sample_path = os.path.join(model_dir, 'X_sample.csv')
                        shap_values = np.load(shap_values_path, allow_pickle=True)
                        with open(feature_names_path, 'r') as f:
                                feature_names = [line.strip() for line in f.readlines()]
                        X_sample = pd.read_csv(sample_path, index_col=0)
                        if isinstance(shap_values, list) and len(shap_values) == 1:
                                shap_values = shap_values[0]
                        if shap_values.shape[0] != X_sample.shape[0]:
                                min_rows = min(shap_values.shape[0], X_sample.shape[0])
                                shap_values = shap_values[:min_rows]
                                X_sample = X_sample.iloc[:min_rows]
                        if shap_values.shape[1] != len(feature_names):
```

```
                if shap_values.shape[1] < len(feature_names):
                    feature_names = feature_names[:shap_values.shape[1]]
                else:
                    feature_names = feature_names + [f"feature_{i}" for i in
range(len(feature_names), shap_values.shape[1])]
            try:
                plt.figure(figsize=(12, 16))
                plt.rcParams.update({'font.size': 36})
                shap.summary_plot(shap_values, X_sample,
feature_names=feature_names, plot_type="dot", show=False, color_bar=True,
max_display=25)
                plt.title(f"{model_name} SHAP Values", fontsize=32)
                plt.tight_layout()
                summary_path = os.path.join(model_viz_dir, f"shap_summary.png")
                plt.savefig(summary_path, dpi=300, bbox_inches='tight')
                plt.close()
                plt.figure(figsize=(12, 8))
                plt.rcParams.update({'font.size': 28})
                shap.summary_plot(shap_values, X_sample,
feature_names=feature_names, plot_type="bar", show=False)
                plt.title(f"{model_name} Feature Importance", fontsize=32)
                plt.tight_layout()
                bar_path = os.path.join(model_viz_dir, f"shap_importance.png")
                plt.savefig(bar_path, dpi=300, bbox_inches='tight')
                plt.close()
                mean_abs_shap = np.abs(shap_values).mean(axis=0)
                feature_importance = pd.DataFrame({'feature': feature_names,
'importance': mean_abs_shap}).sort_values('importance', ascending=False)
                top_n = min(5, len(feature_names))
                top_features = feature_importance['feature'].head(top_n).tolist()
                for feature in top_features:
                    try:
                        feature_idx = feature_names.index(feature)
                        plt.figure(figsize=(10, 7))
                        plt.rcParams.update({'font.size': 20})
                        shap.dependence_plot(feature_idx, shap_values, X_sample,
feature_names=feature_names, show=False)
                        plt.title(f"{model_name} - {feature} Dependence", fontsize=24)
                        plt.tight_layout()
                        safe_feature_name = feature.replace('/', '_').replace('\\',
'_').replace(' ', '_')
                        dep_path = os.path.join(model_viz_dir,
f"{safe_feature_name}_dependence.png")
                        plt.savefig(dep_path, dpi=300, bbox_inches='tight')
```

```
                        plt.close()
                        print(f"save{feature} dependence to: {dep_path}")
                    except Exception as e:
                        print(f"create{feature} dependence error: {str(e)}")
                        continue
            except Exception as e:
                print(f"{model_name}shap error: {str(e)}")
                import traceback
                traceback.print_exc()
                continue
    except Exception as e:
        print(f"shap error: {str(e)}")
        import traceback
        traceback.print_exc()
def visualize_feature_selection(X_train, selected_features, y_train, target_name, save_path='feature_selection_analysis'):
    os.makedirs(save_path, exist_ok=True)
    f_selector = SelectKBest(score_func=f_regression, k='all')
    f_selector.fit(X_train[selected_features], y_train)
    f_scores = pd.Series(f_selector.scores_, index=selected_features)
    correlations = X_train[selected_features].corrwith(y_train).abs()
    mi_scores = mutual_info_regression(X_train[selected_features], y_train)
    mi_series = pd.Series(mi_scores, index=selected_features)
    feature_scores = pd.DataFrame({
        'F-regression': f_scores,
        'Correlation': correlations,
        'Mutual Information': mi_series
    })
    feature_scores_norm = (feature_scores - feature_scores.min()) / (feature_scores.max() - feature_scores.min())
    composite_score = feature_scores_norm.mean(axis=1).sort_values(ascending=False)
    plt.figure(figsize=(20, 15))
    gs = gridspec.GridSpec(2, 2)
    ax1 = plt.subplot(gs[0, 0])
    top_features = composite_score.head(15)
    colors = plt.cm.viridis(np.linspace(0, 1, len(top_features)))
    bars = ax1.barh(range(len(top_features)), top_features.values, color=colors)
    ax1.set_yticks(range(len(top_features)))
    ax1.set_yticklabels(top_features.index, fontsize=8)
    ax1.set_title('Top 15 Features (Composite Score)')
    for i, bar in enumerate(bars):
        width = bar.get_width()
        ax1.text(width, bar.get_y() + bar.get_height()/2,
                f'{width:.3f}',
```

```
                        ha='left', va='center', fontsize=8)
    ax2 = plt.subplot(gs[0, 1])
    top_features_scores = feature_scores_norm.loc[top_features.index]
    sns.heatmap(top_features_scores.T, cmap='YlOrRd',
                        annot=True, fmt='.2f', ax=ax2)
    ax2.set_title('Feature Selection Metrics Comparison')
    ax3 = plt.subplot(gs[1, :])
    corr_matrix = X_train[top_features.index].corr()
    G = nx.Graph()
    for i in range(len(corr_matrix)):
        for j in range(i+1, len(corr_matrix)):
            if abs(corr_matrix.iloc[i, j]) > 0.3:
                G.add_edge(corr_matrix.index[i],
                                corr_matrix.index[j],
                                weight=abs(corr_matrix.iloc[i, j]))
    pos = nx.spring_layout(G, k=1, iterations=50)
    edges = G.edges()
    weights = [G[u][v]['weight'] for u, v in edges]
    edge_collection = nx.draw_networkx_edges(G, pos,
                                                edge_color=weights,
                                                edge_cmap=plt.cm.YlOrRd,
                                                width=2,
                                                alpha=0.5)
    node_sizes = composite_score[list(G.nodes())] * 1000
    node_collection = nx.draw_networkx_nodes(G, pos,
                                                node_size=node_sizes,

node_color=list(composite_score[list(G.nodes())]),
                                                cmap=plt.cm.viridis,
                                                alpha=0.7)
    nx.draw_networkx_labels(G, pos, font_size=8)
    plt.colorbar(edge_collection, ax=ax3, label='Correlation Strength',
                        orientation='vertical', fraction=0.02, pad=0.04)
    plt.colorbar(node_collection, ax=ax3, label='Feature Importance',
                        orientation='vertical', fraction=0.02, pad=0.08)
    ax3.set_title('Feature Correlation Network\n(Node size: importance, Edge: correlation
strength)')
    plt.suptitle(f'Feature Selection Analysis - {target_name}',
                         y=1.02, size=16)
    plt.tight_layout()
    plt.savefig(os.path.join(save_path, f'{target_name}_feature_selection_analysis.png'),
                        dpi=300, bbox_inches='tight')
    plt.close()
    selection_summary = {
```

```
            'total_features': len(X_train.columns),
            'selected_features': len(selected_features),
            'selection_ratio': len(selected_features) / len(X_train.columns),
            'top_features': composite_score.head(10).to_dict(),
            'feature_scores': feature_scores.to_dict()
        }
        with open(os.path.join(save_path, f'{target_name}_feature_selection_summary.json'), 'w') as f:
            json.dump(selection_summary, f, indent=4)
        print(f"total features: {len(X_train.columns)}")
        print(f"selected features: {len(selected_features)}")
        print(f"select ratio: {len(selected_features)/len(X_train.columns):.2%}")
        return selection_summary
def calculate_shap_values(model, X, feature_names=None, sample_size=1000):
    try:
        import shap
        if feature_names is None:
            feature_names = X.columns.tolist()
        os.makedirs('shap_analysis', exist_ok=True)
        if len(X) > sample_size:
            X_sample = X.sample(sample_size, random_state=42)
        else:
            X_sample = X
        model_type = type(model).__name__
        print(f"Process model type: {model_type}")
        if 'CatBoostRegressor' in model_type or 'CatBoost' in model_type:
            print("Using CatBoost-specific SHAP calculation method...")
            try:
                max_shap_samples = min(500, len(X_sample))
                X_shap_sample = X_sample.iloc[:max_shap_samples]
                shap_values = model.get_feature_importance(data=X_shap_sample, type='ShapValues')
                if shap_values.shape[1] == len(feature_names) + 1:
                    base_value = shap_values[:, -1].mean()
                    shap_values = shap_values[:, :-1]
                else:
                    base_value = 0
                used_explainer = "CatBoost Native"
                X_sample = X_shap_sample
            except Exception as cat_error:
                print(f"CatBoost native SHAP calculation failed: {str(cat_error)}")
                print("Falling back to TreeExplainer...")
                try:
                    explainer = shap.TreeExplainer(model)
```

```
                max_shap_samples = min(300, len(X_sample))
                X_shap_sample = X_sample.iloc[:max_shap_samples]
                shap_values = explainer.shap_values(X_shap_sample)
                used_explainer = "TreeExplainer"
                X_sample = X_shap_sample
            except Exception as tree_error:
                print(f"TreeExplainer also failed: {str(tree_error)}")
                print("Using simplified approach...")
                importances = model.feature_importances_
                importance_df = pd.DataFrame({
                    'feature': feature_names,
                    'importance': importances
                }).sort_values('importance', ascending=False)
                return {
                    'feature_names': feature_names,
                    'importance': importance_df,
                    'X_sample': X_sample,
                    'explainer_type': "Feature Importance"
                }
    elif 'LGBMRegressor' in model_type or 'LGBMModel' in model_type:
        print("Using LightGBM-specific SHAP calculation method...")
        try:
            if hasattr(model, 'booster_'):
                max_shap_samples = min(500, len(X_sample))
                X_shap_sample = X_sample.iloc[:max_shap_samples]
                booster = model.booster_
                shap_values = booster.predict(X_shap_sample, pred_contrib=True)
                if shap_values.shape[1] == len(feature_names) + 1:
                    base_value = shap_values[:, -1].mean()
                    shap_values = shap_values[:, :-1]
                else:
                    base_value = 0
                used_explainer = "LightGBM Native"
                X_sample = X_shap_sample
            else:
                raise AttributeError("LightGBM model lacks booster_ attribute")
        except Exception as lgb_error:
            print(f"LightGBM native SHAP calculation failed: {str(lgb_error)}")
            print("Falling back to TreeExplainer...")
            try:
                explainer = shap.TreeExplainer(model)
                max_shap_samples = min(300, len(X_sample))
                X_shap_sample = X_sample.iloc[:max_shap_samples]
                shap_values = explainer.shap_values(X_shap_sample)
```

```
                used_explainer = "TreeExplainer"
                X_sample = X_shap_sample
            except Exception as tree_error:
                print(f"TreeExplainer also failed: {str(tree_error)}")
                print("Using simplified approach...")
                importances = model.feature_importances_
                importance_df = pd.DataFrame({
                    'feature': feature_names,
                    'importance': importances
                }).sort_values('importance', ascending=False)
                return {
                    'feature_names': feature_names,
                    'importance': importance_df,
                    'X_sample': X_sample,
                    'explainer_type': "Feature Importance"
                }
    elif 'XGBoost' in model_type or 'GradientBoosting' in model_type or 'RandomForest'
in model_type:
        try:
            print(f"Try TreeExplainer...")
            if hasattr(model, 'feature_importances_'):
                model_copy = deepcopy(model)
                if 'XGBoost' in model_type:
                    explainer = shap.TreeExplainer(model)
                else:
                    if not hasattr(model_copy, 'feature_names_in_'):
                        model_copy.feature_names_in_ = feature_names
                    explainer = shap.TreeExplainer(model_copy)
                max_shap_samples = min(500, len(X_sample))
                X_shap_sample = X_sample.iloc[:max_shap_samples]
                shap_values = explainer.shap_values(X_shap_sample)
                if isinstance(shap_values, list) and len(shap_values) == 1:
                    shap_values = shap_values[0]
                used_explainer = "TreeExplainer"
                X_sample = X_shap_sample
            else:
                raise AttributeError("Model lacks essential tree model property")
        except Exception as tree_error:
            print(f"TreeExplainer failed: {str(tree_error)}")
            print("Back to KernelExplainer...")
            try:
                max_background = min(20, len(X_sample))
                background = shap.kmeans(X_sample, max_background)
            except:
```

```
                max_background = min(20, len(X_sample))
                background = X_sample.iloc[:max_background]
            def model_predict_wrapper(X_input):
                try:
                    if isinstance(X_input, np.ndarray):
                        X_input = pd.DataFrame(X_input, columns=feature_names)
                    return model.predict(X_input)
                except Exception as e:
                    print(f"Prediction error: {str(e)}")
                    return np.zeros(X_input.shape[0])
            explainer = shap.KernelExplainer(model_predict_wrapper, background)
            max_explain_samples = min(300, len(X_sample))
            calc_sample = X_sample.iloc[:max_explain_samples]
            shap_values = explainer.shap_values(calc_sample)
            used_explainer = "KernelExplainer"
            X_sample = calc_sample
    else:
        print("For non-tree model, using optimized KernelExplainer...")
        try:
            max_background = min(20, len(X_sample))
            background = shap.kmeans(X_sample, max_background)
        except:
            max_background = min(20, len(X_sample))
            background = X_sample.iloc[:max_background]
        def model_predict_wrapper(X_input):
            try:
                if isinstance(X_input, np.ndarray):
                    X_input = pd.DataFrame(X_input, columns=feature_names)
                return model.predict(X_input)
            except Exception as e:
                print(f"Prediction error: {str(e)}")
                return np.zeros(X_input.shape[0])
        explainer = shap.KernelExplainer(model_predict_wrapper, background)
        max_explain_samples = min(200, len(X_sample))
        calc_sample = X_sample.iloc[:max_explain_samples]
        shap_values = explainer.shap_values(calc_sample)
        used_explainer = "KernelExplainer"
        X_sample = calc_sample
    print(f"Successfully calculated SHAP using {used_explainer}")
    if isinstance(shap_values, list):
        shap_df = pd.DataFrame(shap_values[0], columns=feature_names)
        shap_df.index = X_sample.index
    else:
        shap_df = pd.DataFrame(shap_values, columns=feature_names)
```

```
        shap_df.index = X_sample.index
    plt.figure(figsize=(12, 16))
    try:
        shap.summary_plot(
            shap_values,
            X_sample,
            plot_type="dot",
            feature_names=feature_names,
            show=False,
            color_bar=True,
            max_display=28
        )
        plt.title(f"SHAP Values Summary ({model_type})")
        plt.tight_layout()
        plt.savefig(f'shap_analysis/{model_type.lower()}_shap_summary.png',
                    dpi=300, bbox_inches='tight')
        plt.close()
        plt.figure(figsize=(12, 8))
        shap.summary_plot(
            shap_values,
            X_sample,
            plot_type="bar",
            feature_names=feature_names,
            show=False,
            max_display=28
        )
        plt.title(f"SHAP Feature Importance ({model_type})")
        plt.tight_layout()
        plt.savefig(f'shap_analysis/{model_type.lower()}_shap_importance.png',
                    dpi=300, bbox_inches='tight')
        plt.close()
    except Exception as plot_error:
        print(f"SHAP visualization error: {str(plot_error)}")
    shap_df.to_csv(f'shap_analysis/{model_type.lower()}_shap_values.csv')
    mean_abs_shap = np.abs(shap_df).mean().sort_values(ascending=False)
    importance_df = pd.DataFrame({
        'feature': mean_abs_shap.index,
        'importance': mean_abs_shap.values
    })
    return {
        'shap_values': shap_values,
        'feature_names': feature_names,
        'shap_df': shap_df,
        'importance': importance_df,
```

```
                'X_sample': X_sample,
                'explainer_type': used_explainer
            }
    except Exception as e:
        print(f"SHAP calculation error: {str(e)}")
        traceback.print_exc()
        return None
def preprocess_target(y):
    z_scores = np.abs(stats.zscore(y, nan_policy='omit'))
    outliers_z = y[z_scores > 3]
    q1 = y.quantile(0.25)
    q3 = y.quantile(0.75)
    iqr = q3 - q1
    lower_bound = q1 - 1.5 * iqr
    upper_bound = q3 + 1.5 * iqr
    outliers_iqr = y[(y < lower_bound) | (y > upper_bound)]
    print(f"Z-score detected {len(outliers_z)} outliers ({len(outliers_z)/len(y)*100:.2f}%)")
    print(f"IQR detected {len(outliers_iqr)} outliers ({len(outliers_iqr)/len(y)*100:.2f}%)")
    y_clean = stats.mstats.winsorize(y, limits=[0.05, 0.05])
    print("Using Winsorization to handle outliers")
    if np.isnan(y_clean).any():
        median = np.nanmedian(y_clean)
        y_clean = np.nan_to_num(y_clean, nan=median)
        print(f"Using median {median:.4f} to fill missing values")
    y_clean = pd.Series(y_clean)
    orig_skew = y_clean.skew()
    min_val = y_clean.min()
    if min_val <= 0:
        offset = abs(min_val) + 1
        y_transformed = np.log(y_clean + offset)
    else:
        y_transformed = np.log(y_clean)

    transformed_skew = abs(pd.Series(y_transformed).skew())
    print(f"Applied logarithmic transformation, reducing skewness from {orig_skew:.4f} to
{transformed_skew:.4f}")
    y_clean = y_transformed
    if y_clean.std() > 10 or abs(y_clean.mean()) > 10:
        print("Applying standardization")
        y_clean = (y_clean - y_clean.mean()) / y_clean.std()
    return pd.Series(y_clean)
def validate_feature_groups(X, feature_groups):
    validated_groups = {}
    available_features = set(X.columns)
```

```
    used_features = set()
    for group_name, features in feature_groups.items():
        valid_features = [f for f in features if f in available_features]
        if valid_features:
            validated_groups[group_name] = valid_features
            used_features.update(valid_features)
    remaining_features = available_features - used_features
    if remaining_features:
        if 'other' in validated_groups:
            validated_groups['other'].extend(list(remaining_features))
        else:
            validated_groups['other'] = list(remaining_features)
    print(f"\nFeature group validation results:")
    for group, features in validated_groups.items():
        print(f"    - {group}: {len(features)} features")
    all_assigned = []
    for group_features in validated_groups.values():
        all_assigned.extend(group_features)
    duplicate_features = [f for f in all_assigned if all_assigned.count(f) > 1]
    if duplicate_features:
        duplicates_set = set(duplicate_features)
        print(f"\nWarning: Found {len(duplicates_set)} features appearing in multiple
groups:")
        for feature in list(duplicates_set)[:5]:
            groups_containing = [g for g, fs in validated_groups.items() if feature in fs]
            print(f"    - '{feature}' appears in: {', '.join(groups_containing)}")
        if len(duplicates_set) > 5:
            print(f"    - ... and {len(duplicates_set) - 5} other duplicate features")
    return validated_groups
def plot_feature_group_importance(detailed_results, save_path, target_name):
    plt.figure(figsize=(15, 10))
    colors = plt.cm.Pastel1(np.linspace(0, 1, len(detailed_results)))
    all_groups = set()
    for results in detailed_results.values():
        all_groups.update(results['group_importance'].keys())
    group_importance_avg = {group: [] for group in all_groups}
    for model_results in detailed_results.values():
        for group in all_groups:
            if group in model_results['group_importance']:
                group_importance_avg[group].append(
                    model_results['group_importance'][group]
                )
    group_stats = {}
    for group in all_groups:
```

```
            values = group_importance_avg[group]
            if values:
                group_stats[group] = {
                    'mean': np.mean(values),
                    'std': np.std(values)
                }
        sorted_groups = sorted(
            group_stats.items(),
            key=lambda x: x[1]['mean'],
            reverse=True
        )
        y_pos = np.arange(len(sorted_groups))
        means = [stats['mean'] for _, stats in sorted_groups]
        stds = [stats['std'] for _, stats in sorted_groups]
        group_names = [group for group, _ in sorted_groups]
        plt.barh(y_pos, means, xerr=stds, align='center',
                 color=colors, alpha=0.6, ecolor='black',
                 capsize=5)
        plt.yticks(y_pos, group_names)
        plt.xlabel('Feature Group Importance (Performance Drop)')
        plt.title(f'{target_name} - Feature Group Importance Analysis')
        plt.grid(True, alpha=0.3)
        plt.tight_layout()
        plt.savefig(os.path.join(save_path, f'{target_name}_feature_group_importance.png'),
                    dpi=300, bbox_inches='tight')
        plt.close()
        results_dict = {
            group: {
                'mean_importance': float(stats['mean']),
                'std_importance': float(stats['std'])
            }
            for group, stats in group_stats.items()
        }
        with open(os.path.join(save_path, f'{target_name}_feature_group_importance.json'), 'w')
as f:
            json.dump(results_dict, f, indent=4)
def grid_search_models(X_train, y_train, param_grids, cv=5):
    from sklearn.model_selection import ParameterGrid, cross_val_score
    from sklearn.metrics import make_scorer, mean_squared_error, mean_absolute_error,
r2_score
    best_models = {}
    best_params = {}
    for model_name, param_grid in param_grids.items():
        print(f"\nfor {model_name} grid search:")
```

```
        if model_name == 'CatBoost':
            if 'iterations' in param_grid:
                fixed_iterations = max(param_grid['iterations'])
                param_grid = {k: v for k, v in param_grid.items() if k != 'iterations'}
                param_grid_for_search = param_grid.copy()
                print(f"Fixed iterations to {fixed_iterations} to speed up CatBoost parameter
search")
            else:
                fixed_iterations = 500
                param_grid_for_search = param_grid.copy()
            param_combinations = list(ParameterGrid(param_grid_for_search))
            best_score = -np.inf
            best_model = None
            best_param = None
            for params in param_combinations:
                params_with_iterations = params.copy()
                params_with_iterations['iterations'] = fixed_iterations
                params_with_iterations['random_seed'] = 42
                params_with_iterations['verbose'] = False
                cat_model = CatBoostRegressor(**params_with_iterations)
                r2_scores = cross_val_score(cat_model, X_train, y_train, cv=cv, scoring='r2',
n_jobs=-1)
                rmse_scores = -cross_val_score(cat_model, X_train, y_train, cv=cv,
scoring='neg_root_mean_squared_error', n_jobs=-1)
                mae_scores = -cross_val_score(cat_model, X_train, y_train, cv=cv,
scoring='neg_mean_absolute_error', n_jobs=-1)
                mean_r2 = r2_scores.mean()
                mean_rmse = rmse_scores.mean()
                mean_mae = mae_scores.mean()
                print(f"CatBoost CV metrics - R2: {mean_r2:.4f}, RMSE: {mean_rmse:.4f},
MAE: {mean_mae:.4f}")
                if mean_r2 > best_score:
                    best_score = mean_r2
                    best_param = params_with_iterations
                    cat_model = CatBoostRegressor(**params_with_iterations)
                    cat_model.fit(X_train, y_train)
                    best_model = cat_model
            best_models[model_name] = best_model
            best_params[model_name] = best_param
            print(f"best parameter: {best_param}")
            print(f"best score: {best_score:.4f}")
        elif model_name == 'XGBoost':
            model = xgb.XGBRegressor(random_state=42)
            search_spaces = {}
```

```
        for param_name, param_values in param_grid.items():
            if param_name == 'n_jobs':
                search_spaces[param_name] = Categorical(param_values)
                continue
            if all(isinstance(v, int) for v in param_values):
                min_val, max_val = min(param_values), max(param_values)
                search_spaces[param_name] = Integer(min_val, max_val)
            elif all(isinstance(v, float) for v in param_values):
                min_val, max_val = min(param_values), max(param_values)
                if min_val <= 0 and param_name in ['learning_rate', 'reg_alpha', 'reg_lambda']:
                    min_val_adj = 1e-6 if min_val == 0 else min_val
                    search_spaces[param_name] = Real(min_val_adj, max_val, prior='log-uniform')
                else:
                    search_spaces[param_name] = Real(min_val, max_val, prior='uniform')
            else:
                search_spaces[param_name] = Categorical(param_values)
        try:
            bayes_search = BayesSearchCV(
                model,
                search_spaces,
                n_iter=50,
                cv=cv,
                scoring='r2',
                n_jobs=-1,
                verbose=1,
                random_state=42
            )
            bayes_search.fit(X_train, y_train)
            best_models[model_name] = bayes_search.best_estimator_
            best_params[model_name] = bayes_search.best_params_
            r2_scores = cross_val_score(bayes_search.best_estimator_, X_train, y_train, cv=cv, scoring='r2', n_jobs=-1)
            rmse_scores = -cross_val_score(bayes_search.best_estimator_, X_train, y_train, cv=cv, scoring='neg_root_mean_squared_error', n_jobs=-1)
            mae_scores = -cross_val_score(bayes_search.best_estimator_, X_train, y_train, cv=cv, scoring='neg_mean_absolute_error', n_jobs=-1)
            print(f"XGBoost CV metrics - R2: {r2_scores.mean():.4f}, RMSE: {rmse_scores.mean():.4f}, MAE: {mae_scores.mean():.4f}")
            print(f"best parameter: {bayes_search.best_params_}")
            print(f"best score: {bayes_search.best_score_:.4f}")
        except Exception as e:
```

```
                print(f"Error during Bayesian search for {model_name}: {e}")
                random_search = RandomizedSearchCV(
                    model,
                    param_grid,
                    n_iter=50,
                    cv=cv,
                    scoring='r2',
                    n_jobs=-1,
                    verbose=1,
                    random_state=42
                )
                random_search.fit(X_train, y_train)
                best_models[model_name] = random_search.best_estimator_
                best_params[model_name] = random_search.best_params_
                r2_scores = cross_val_score(random_search.best_estimator_, X_train,
y_train, cv=cv, scoring='r2', n_jobs=-1)
                rmse_scores = -cross_val_score(random_search.best_estimator_, X_train,
y_train, cv=cv, scoring='neg_root_mean_squared_error', n_jobs=-1)
                mae_scores = -cross_val_score(random_search.best_estimator_, X_train,
y_train, cv=cv, scoring='neg_mean_absolute_error', n_jobs=-1)
                print(f"XGBoost CV metrics - R2: {r2_scores.mean():.4f}, RMSE:
{rmse_scores.mean():.4f}, MAE: {mae_scores.mean():.4f}")
                print(f"best parameter: {random_search.best_params_}")
                print(f"best score: {random_search.best_score_:.4f}")
        elif model_name == 'LightGBM':
            model = LGBMRegressor(random_state=42)
            search_spaces = _create_lightgbm_search_space(param_grid)
            try:
                bayes_search = BayesSearchCV(
                    model,
                    search_spaces,
                    n_iter=50,
                    cv=cv,
                    scoring='r2',
                    n_jobs=-1,
                    verbose=1,
                    random_state=42
                )
                bayes_search.fit(X_train, y_train)
                best_models[model_name] = bayes_search.best_estimator_
                best_params[model_name] = bayes_search.best_params_
                r2_scores = cross_val_score(bayes_search.best_estimator_, X_train,
y_train, cv=cv, scoring='r2', n_jobs=-1)
                rmse_scores = -cross_val_score(bayes_search.best_estimator_, X_train,
```

```
y_train, cv=cv, scoring='neg_root_mean_squared_error', n_jobs=-1)
                mae_scores = -cross_val_score(bayes_search.best_estimator_, X_train, y_train, cv=cv, scoring='neg_mean_absolute_error', n_jobs=-1)
                print(f"LightGBM CV metrics - R2: {r2_scores.mean():.4f}, RMSE: {rmse_scores.mean():.4f}, MAE: {mae_scores.mean():.4f}")
                print(f"best parameter: {bayes_search.best_params_}")
                print(f"best score: {bayes_search.best_score_:.4f}")
            except Exception as e:
                print(f"Error during Bayesian search for {model_name}: {e}")
                random_search = RandomizedSearchCV(
                    model,
                    param_grid,
                    n_iter=50,
                    cv=cv,
                    scoring='r2',
                    n_jobs=-1,
                    verbose=1,
                    random_state=42
                )
                random_search.fit(X_train, y_train)
                best_models[model_name] = random_search.best_estimator_
                best_params[model_name] = random_search.best_params_
                r2_scores = cross_val_score(random_search.best_estimator_, X_train, y_train, cv=cv, scoring='r2', n_jobs=-1)
                rmse_scores = -cross_val_score(random_search.best_estimator_, X_train, y_train, cv=cv, scoring='neg_root_mean_squared_error', n_jobs=-1)
                mae_scores = -cross_val_score(random_search.best_estimator_, X_train, y_train, cv=cv, scoring='neg_mean_absolute_error', n_jobs=-1)
                print(f"LightGBM CV metrics - R2: {r2_scores.mean():.4f}, RMSE: {rmse_scores.mean():.4f}, MAE: {mae_scores.mean():.4f}")
                print(f"best parameter: {random_search.best_params_}")
                print(f"best score: {random_search.best_score_:.4f}")
        elif model_name == 'RandomForest':
            model = RandomForestRegressor(random_state=42)
            if 'class_weight' in param_grid:
                print("Warning: 'class_weight' parameter is not supported for RandomForestRegressor, removing it.")
                param_grid = {k: v for k, v in param_grid.items() if k != 'class_weight'}
            if 'criterion' in param_grid:
                valid_criteria = ['squared_error', 'absolute_error', 'friedman_mse', 'poisson']
                param_grid['criterion'] = [c for c in param_grid['criterion'] if c in valid_criteria]
                if not param_grid['criterion']:
```

```
                        param_grid['criterion'] = ['squared_error']
                    print(f"Using criterion values for RandomForestRegressor:
{param_grid['criterion']}")
                search_spaces = _create_randomforest_search_space(param_grid)
                if isinstance(search_spaces, dict) and 'bootstrap_true' in search_spaces and
'bootstrap_false' in search_spaces:
                    print("Handling special case for RandomForest with
bootstrap/max_samples dependency")
                    best_score = float('-inf')
                    best_estimator = None
                    best_param_set = None
                    for space_name, space in search_spaces.items():
                        try:
                            print(f"Trying search space: {space_name}")
                            bayes_search = BayesSearchCV(
                                RandomForestRegressor(random_state=42),
                                space,
                                n_iter=25,
                                cv=cv,
                                scoring='r2',
                                n_jobs=-1,
                                verbose=1,
                                random_state=42
                            )
                            bayes_search.fit(X_train, y_train)
                            r2_scores = cross_val_score(bayes_search.best_estimator_,
X_train, y_train, cv=cv, scoring='r2', n_jobs=-1)
                            rmse_scores = -cross_val_score(bayes_search.best_estimator_,
X_train, y_train, cv=cv, scoring='neg_root_mean_squared_error', n_jobs=-1)
                            mae_scores = -cross_val_score(bayes_search.best_estimator_,
X_train, y_train, cv=cv, scoring='neg_mean_absolute_error', n_jobs=-1)
                            print(f"RandomForest ({space_name}) CV metrics - R2:
{r2_scores.mean():.4f}, RMSE: {rmse_scores.mean():.4f}, MAE: {mae_scores.mean():.4f}")
                            if bayes_search.best_score_ > best_score:
                                best_score = bayes_search.best_score_
                                best_estimator = bayes_search.best_estimator_
                                best_param_set = bayes_search.best_params_
                                print(f"New best score from {space_name}:
{best_score:.4f}")
                        except Exception as e:
                            print(f"Error with {space_name} search space: {e}")
                    if best_estimator is not None:
                        best_models[model_name] = best_estimator
                        best_params[model_name] = best_param_set
```

```
                print(f"Final best parameter: {best_param_set}")
                print(f"Final best score: {best_score:.4f}")
                continue
            else:
                print("Both search spaces failed, falling back to default parameters")
                default_model = RandomForestRegressor(random_state=42)
                default_model.fit(X_train, y_train)
                best_models[model_name] = default_model
                best_params[model_name] = {}
                r2_scores = cross_val_score(default_model, X_train, y_train, cv=cv, scoring='r2', n_jobs=-1)
                rmse_scores = -cross_val_score(default_model, X_train, y_train, cv=cv, scoring='neg_root_mean_squared_error', n_jobs=-1)
                mae_scores = -cross_val_score(default_model, X_train, y_train, cv=cv, scoring='neg_mean_absolute_error', n_jobs=-1)
                print(f"RandomForest (default) CV metrics - R2: {r2_scores.mean():.4f}, RMSE: {rmse_scores.mean():.4f}, MAE: {mae_scores.mean():.4f}")
                continue
        if model_name == 'RandomForest' and 'bootstrap' in search_spaces and 'max_samples' in search_spaces:
            bootstrap_values = search_spaces['bootstrap'].categories
            if False in bootstrap_values and len(bootstrap_values) > 1:
                print("Warning: When bootstrap=False, max_samples cannot be set.")
                print("Modifying search space to avoid this constraint...")
                search_spaces.pop('max_samples')
                print("Removed max_samples parameter from search space.")
        try:
            bayes_search = BayesSearchCV(
                model,
                search_spaces,
                n_iter=50,
                cv=cv,
                scoring='r2',
                n_jobs=-1,
                verbose=1,
                random_state=42
            )
            bayes_search.fit(X_train, y_train)
            best_models[model_name] = bayes_search.best_estimator_
            best_params[model_name] = bayes_search.best_params_
            r2_scores = cross_val_score(bayes_search.best_estimator_, X_train, y_train, cv=cv, scoring='r2', n_jobs=-1)
            rmse_scores = -cross_val_score(bayes_search.best_estimator_, X_train, y_train, cv=cv, scoring='neg_root_mean_squared_error', n_jobs=-1)
```

```
                mae_scores = -cross_val_score(bayes_search.best_estimator_, X_train,
y_train, cv=cv, scoring='neg_mean_absolute_error', n_jobs=-1)
                print(f"RandomForest CV metrics - R2: {r2_scores.mean():.4f}, RMSE:
{rmse_scores.mean():.4f}, MAE: {mae_scores.mean():.4f}")
                print(f"best parameter: {bayes_search.best_params_}")
                print(f"best score: {bayes_search.best_score_:.4f}")
            except Exception as e:
                print(f"Error during Bayesian search for {model_name}: {e}")
                if 'bootstrap' in param_grid and 'max_samples' in param_grid:
                    print("Trying simplified RandomForest parameters without
max_samples...")
                    simplified_param_grid = {k: v for k, v in param_grid.items() if k !=
'max_samples'}
                    simplified_search_spaces =
_create_randomforest_search_space(simplified_param_grid)
                    try:
                        simplified_bayes = BayesSearchCV(
                            RandomForestRegressor(random_state=42),
                            simplified_search_spaces,
                            n_iter=30,
                            cv=cv,
                            scoring='r2',
                            n_jobs=-1,
                            verbose=1,
                            random_state=42
                        )
                        simplified_bayes.fit(X_train, y_train)
                        best_models[model_name] = simplified_bayes.best_estimator_
                        best_params[model_name] = simplified_bayes.best_params_
                        r2_scores = cross_val_score(simplified_bayes.best_estimator_,
X_train, y_train, cv=cv, scoring='r2', n_jobs=-1)
                        rmse_scores = -
cross_val_score(simplified_bayes.best_estimator_, X_train, y_train, cv=cv,
scoring='neg_root_mean_squared_error', n_jobs=-1)
                        mae_scores = -
cross_val_score(simplified_bayes.best_estimator_, X_train, y_train, cv=cv,
scoring='neg_mean_absolute_error', n_jobs=-1)
                        print(f"RandomForest (simplified) CV metrics - R2:
{r2_scores.mean():.4f}, RMSE: {rmse_scores.mean():.4f}, MAE: {mae_scores.mean():.4f}")
                        print(f"best parameter: {simplified_bayes.best_params_}")
                        print(f"best score: {simplified_bayes.best_score_:.4f}")
                    except Exception as e2:
                        print(f"Second attempt also failed: {e2}")
                        random_search = RandomizedSearchCV(
```

```
                model,
                param_grid,
                n_iter=50,
                cv=cv,
                scoring='r2',
                n_jobs=-1,
                verbose=1,
                random_state=42
            )
            random_search.fit(X_train, y_train)
            best_models[model_name] = random_search.best_estimator_
            best_params[model_name] = random_search.best_params_
            r2_scores = cross_val_score(random_search.best_estimator_,
X_train, y_train, cv=cv, scoring='r2', n_jobs=-1)
            rmse_scores = -cross_val_score(random_search.best_estimator_,
X_train, y_train, cv=cv, scoring='neg_root_mean_squared_error', n_jobs=-1)
            mae_scores = -cross_val_score(random_search.best_estimator_,
X_train, y_train, cv=cv, scoring='neg_mean_absolute_error', n_jobs=-1)
            print(f"RandomForest (random search) CV metrics - R2:
{r2_scores.mean():.4f}, RMSE: {rmse_scores.mean():.4f}, MAE: {mae_scores.mean():.4f}")
            print(f"best parameter: {random_search.best_params_}")
            print(f"best score: {random_search.best_score_:.4f}")
    else:
        random_search = RandomizedSearchCV(
            model,
            param_grid,
            n_iter=50,
            cv=cv,
            scoring='r2',
            n_jobs=-1,
            verbose=1,
            random_state=42
        )
        random_search.fit(X_train, y_train)
        best_models[model_name] = random_search.best_estimator_
        best_params[model_name] = random_search.best_params_
        r2_scores = cross_val_score(random_search.best_estimator_, X_train,
y_train, cv=cv, scoring='r2', n_jobs=-1)
        rmse_scores = -cross_val_score(random_search.best_estimator_,
X_train, y_train, cv=cv, scoring='neg_root_mean_squared_error', n_jobs=-1)
        mae_scores = -cross_val_score(random_search.best_estimator_,
X_train, y_train, cv=cv, scoring='neg_mean_absolute_error', n_jobs=-1)
        print(f"RandomForest (random search) CV metrics - R2:
{r2_scores.mean():.4f}, RMSE: {rmse_scores.mean():.4f}, MAE: {mae_scores.mean():.4f}")
```

```
                                        print(f"best parameter: {random_search.best_params_}")
                                        print(f"best score: {random_search.best_score_:.4f}")
                elif model_name == 'GaussianProcess':
                        model = GaussianProcessRegressor(random_state=42)
                        search_spaces = {}
                        for param_name, param_values in param_grid.items():
                                if param_name == 'n_jobs':
                                        search_spaces[param_name] = Categorical(param_values)
                                        continue
                                if all(isinstance(v, int) for v in param_values):
                                        min_val, max_val = min(param_values), max(param_values)
                                        search_spaces[param_name] = Integer(min_val, max_val)
                                elif all(isinstance(v, float) for v in param_values):
                                        min_val, max_val = min(param_values), max(param_values)
                                        if min_val <= 0 and param_name in ['alpha']:
                                                min_val_adj = 1e-6 if min_val == 0 else min_val
                                                search_spaces[param_name] = Real(min_val_adj, max_val,
prior='log-uniform')
                                        else:
                                                search_spaces[param_name] = Real(min_val, max_val,
prior='uniform')
                                else:
                                        search_spaces[param_name] = Categorical(param_values)
                        try:
                                bayes_search = BayesSearchCV(
                                        model,
                                        search_spaces,
                                        n_iter=50,
                                        cv=cv,
                                        scoring='r2',
                                        n_jobs=-1,
                                        verbose=1,
                                        random_state=42
                                )
                                bayes_search.fit(X_train, y_train)
                                best_models[model_name] = bayes_search.best_estimator_
                                best_params[model_name] = bayes_search.best_params_
                                r2_scores = cross_val_score(bayes_search.best_estimator_, X_train,
y_train, cv=cv, scoring='r2', n_jobs=-1)
                                rmse_scores = -cross_val_score(bayes_search.best_estimator_, X_train,
y_train, cv=cv, scoring='neg_root_mean_squared_error', n_jobs=-1)
                                mae_scores = -cross_val_score(bayes_search.best_estimator_, X_train,
y_train, cv=cv, scoring='neg_mean_absolute_error', n_jobs=-1)
                                print(f"GaussianProcess CV metrics - R2: {r2_scores.mean():.4f}, RMSE:
```

```
{rmse_scores.mean():.4f}, MAE: {mae_scores.mean():.4f}")
                print(f"best parameter: {bayes_search.best_params_}")
                print(f"best score: {bayes_search.best_score_:.4f}")
            except Exception as e:
                print(f"Error during Bayesian search for {model_name}: {e}")
                random_search = RandomizedSearchCV(
                    model,
                    param_grid,
                    n_iter=50,
                    cv=cv,
                    scoring='r2',
                    n_jobs=-1,
                    verbose=1,
                    random_state=42
                )
                random_search.fit(X_train, y_train)
                best_models[model_name] = random_search.best_estimator_
                best_params[model_name] = random_search.best_params_
                r2_scores = cross_val_score(random_search.best_estimator_, X_train,
y_train, cv=cv, scoring='r2', n_jobs=-1)
                rmse_scores = -cross_val_score(random_search.best_estimator_, X_train,
y_train, cv=cv, scoring='neg_root_mean_squared_error', n_jobs=-1)
                mae_scores = -cross_val_score(random_search.best_estimator_, X_train,
y_train, cv=cv, scoring='neg_mean_absolute_error', n_jobs=-1)
                print(f"GaussianProcess (random search) CV metrics - R2:
{r2_scores.mean():.4f}, RMSE: {rmse_scores.mean():.4f}, MAE: {mae_scores.mean():.4f}")
                print(f"best parameter: {random_search.best_params_}")
                print(f"best score: {random_search.best_score_:.4f}")
    return best_models, best_params
def _create_randomforest_search_space(param_grid):
    search_spaces = {}
    bool_params = ['bootstrap', 'oob_score', 'warm_start']
    unsupported_params = ['class_weight']
    regressor_criteria = ['squared_error', 'absolute_error', 'friedman_mse', 'poisson']
    if 'bootstrap' in param_grid:
        bootstrap_values = []
        for value in param_grid['bootstrap']:
            if isinstance(value, (bool, np.bool_)):
                bootstrap_values.append(value)
            elif value == 0:
                bootstrap_values.append(False)
            elif value == 1:
                bootstrap_values.append(True)
        if not bootstrap_values:
```

```
            bootstrap_values = [True]
        if 'max_samples' in param_grid and False in bootstrap_values:
            bootstrap_true_params = {}
            bootstrap_false_params = {}
            bootstrap_true_params['bootstrap'] = Categorical([True])
            bootstrap_false_params['bootstrap'] = Categorical([False])
            if 'max_samples' in param_grid:
                max_samples_values = param_grid['max_samples']
                if all(isinstance(v, float) for v in max_samples_values):
                    min_val, max_val = min(max_samples_values), max(max_samples_values)
                    bootstrap_true_params['max_samples'] = Real(min_val, max_val, prior='uniform')
                else:
                    bootstrap_true_params['max_samples'] = Categorical(max_samples_values)
            other_params = {}
            for param_name, param_values in param_grid.items():
                if param_name in ['bootstrap', 'max_samples']:
                    continue
                if param_name in unsupported_params:
                    print(f"Warning: Parameter '{param_name}' is not supported by RandomForestRegressor, skipping.")
                    continue
                if param_name == 'criterion':
                    valid_criteria = [c for c in param_values if c in regressor_criteria]
                    if not valid_criteria:
                        print(f"Warning: None of the provided criteria {param_values} are valid for RandomForestRegressor. Using default 'squared_error'.")
                        valid_criteria = ['squared_error']
                    other_params[param_name] = Categorical(valid_criteria)
                    continue
                if param_name == 'oob_score':
                    oob_values = []
                    for value in param_values:
                        if isinstance(value, (bool, np.bool_)):
                            oob_values.append(value)
                        elif value == 0:
                            oob_values.append(False)
                        elif value == 1:
                            oob_values.append(True)
                    if not oob_values:
                        oob_values = [False]
                    bootstrap_true_params[param_name] = Categorical(oob_values)
```

```
        bootstrap_false_params[param_name] = Categorical([False])
        continue
    if param_name in bool_params:
        bool_values = []
        for value in param_values:
            if isinstance(value, (bool, np.bool_)):
                bool_values.append(value)
            elif value == 0:
                bool_values.append(False)
            elif value == 1:
                bool_values.append(True)
            else:
                print(f"Warning: Invalid value {value} for boolean parameter {param_name}, skipping")
                continue
        if not bool_values:
            bool_values = [False]
        other_params[param_name] = Categorical(bool_values)
        continue
    if param_name in ['n_jobs', 'random_state']:
        other_params[param_name] = Categorical(param_values)
        continue
    if param_name in ['max_depth', 'max_leaf_nodes'] and None in param_values:
        other_params[param_name] = Categorical(param_values)
        continue
    if param_name == 'max_features':
        other_params[param_name] = Categorical(param_values)
        continue
    if all(isinstance(v, int) for v in param_values):
        if len(param_values) >= 2:
            min_val, max_val = min(param_values), max(param_values)
            other_params[param_name] = Integer(min_val, max_val)
        else:
            other_params[param_name] = Categorical(param_values)
    elif all(isinstance(v, float) for v in param_values):
        if len(param_values) >= 2:
            min_val, max_val = min(param_values), max(param_values)
            if param_name == 'min_impurity_decrease' and min_val > 0:
                other_params[param_name] = Real(min_val, max_val, prior='log-uniform')
            elif min_val > 0 and max_val / min_val > 10:
                other_params[param_name] = Real(min_val, max_val, prior='log-uniform')
```

```
                    else:
                        other_params[param_name] = Real(min_val, max_val, prior='uniform')
                else:
                    other_params[param_name] = Categorical(param_values)
            else:
                other_params[param_name] = Categorical(param_values)
        bootstrap_true_params.update(other_params)
        bootstrap_false_params.update(other_params)
        print("Created two separate search spaces for RandomForest:")
        print(f"1. With bootstrap=True and max_samples specified")
        print(f"2. With bootstrap=False and no max_samples")
        return {
            'bootstrap_true': bootstrap_true_params,
            'bootstrap_false': bootstrap_false_params
        }
    for param_name, param_values in param_grid.items():
        if param_name in unsupported_params:
            print(f"Warning: Parameter '{param_name}' is not supported by RandomForestRegressor, skipping.")
            continue
        if param_name == 'criterion':
            valid_criteria = [c for c in param_values if c in regressor_criteria]
            if not valid_criteria:
                print(f"Warning: None of the provided criteria {param_values} are valid for RandomForestRegressor. Using default 'squared_error'.")
                valid_criteria = ['squared_error']
            search_spaces[param_name] = Categorical(valid_criteria)
            continue
        if param_name in bool_params:
            bool_values = []
            for value in param_values:
                if isinstance(value, (bool, np.bool_)):
                    bool_values.append(value)
                elif value == 0:
                    bool_values.append(False)
                elif value == 1:
                    bool_values.append(True)
                else:
                    print(f"Warning: Invalid value {value} for boolean parameter {param_name}, skipping")
                    continue
            if bool_values:
                search_spaces[param_name] = Categorical(bool_values)
```

```
                else:
                    default_value = True if param_name == 'bootstrap' else False
                    search_spaces[param_name] = Categorical([default_value])
                continue
            if param_name in ['n_jobs', 'random_state']:
                search_spaces[param_name] = Categorical(param_values)
                continue
            if param_name in ['max_depth', 'max_leaf_nodes'] and None in param_values:
                search_spaces[param_name] = Categorical(param_values)
                continue
            if param_name == 'max_features':
                search_spaces[param_name] = Categorical(param_values)
                continue
            if all(isinstance(v, int) for v in param_values):
                if len(param_values) >= 2:
                    min_val, max_val = min(param_values), max(param_values)
                    search_spaces[param_name] = Integer(min_val, max_val)
                else:
                    search_spaces[param_name] = Categorical(param_values)
            elif all(isinstance(v, float) for v in param_values):
                if len(param_values) >= 2:
                    min_val, max_val = min(param_values), max(param_values)
                    if param_name == 'min_impurity_decrease' and min_val > 0:
                        search_spaces[param_name] = Real(min_val, max_val, prior='log-uniform')
                    elif min_val > 0 and max_val / min_val > 10:
                        search_spaces[param_name] = Real(min_val, max_val, prior='log-uniform')
                    else:
                        search_spaces[param_name] = Real(min_val, max_val, prior='uniform')
                else:
                    search_spaces[param_name] = Categorical(param_values)
            else:
                search_spaces[param_name] = Categorical(param_values)
        return search_spaces
def _create_lightgbm_search_space(param_grid):
    search_spaces = {}
    if 'boosting_type' in param_grid:
        search_spaces['boosting_type'] = Categorical(param_grid['boosting_type'])
    if 'num_boost_round' in param_grid:
        search_spaces['n_estimators'] = Integer(min(param_grid['num_boost_round']), max(param_grid['num_boost_round']))
    if 'max_depth' in param_grid:
```

```
            depth_values = param_grid['max_depth']
            if -1 in depth_values:
                positive_depths = [d for d in depth_values if d > 0]
                if positive_depths:
                    search_spaces['max_depth'] = Categorical([-1] + positive_depths)
                else:
                    search_spaces['max_depth'] = Categorical([-1])
            else:
                search_spaces['max_depth'] = Integer(min(depth_values), max(depth_values))
        if 'num_leaves' in param_grid:
            search_spaces['num_leaves'] = Integer(min(param_grid['num_leaves']),
max(param_grid['num_leaves']))
        if 'learning_rate' in param_grid:
            min_lr = min(param_grid['learning_rate'])
            max_lr = max(param_grid['learning_rate'])
            search_spaces['learning_rate'] = Real(min_lr, max_lr, prior='log-uniform')
        if 'min_data_in_leaf' in param_grid:
            search_spaces['min_child_samples'] = Integer(min(param_grid['min_data_in_leaf']),
max(param_grid['min_data_in_leaf']))
        if 'bagging_fraction' in param_grid:
            search_spaces['subsample'] = Real(min(param_grid['bagging_fraction']),
max(param_grid['bagging_fraction']), prior='uniform')
        if 'feature_fraction' in param_grid:
            search_spaces['colsample_bytree'] = Real(min(param_grid['feature_fraction']),
max(param_grid['feature_fraction']), prior='uniform')
        if 'lambda_l1' in param_grid:
            lambda_l1_values = param_grid['lambda_l1']
            if 0 in lambda_l1_values:
                non_zero_values = [v for v in lambda_l1_values if v > 0]
                if non_zero_values:
                    search_spaces['reg_alpha'] = Categorical([0] + non_zero_values)
                else:
                    search_spaces['reg_alpha'] = Categorical([0])
            else:
                min_val = min(lambda_l1_values)
                max_val = max(lambda_l1_values)
                search_spaces['reg_alpha'] = Real(min_val, max_val, prior='log-uniform')
        if 'lambda_l2' in param_grid:
            lambda_l2_values = param_grid['lambda_l2']
            if 0 in lambda_l2_values:
                non_zero_values = [v for v in lambda_l2_values if v > 0]
                if non_zero_values:
                    search_spaces['reg_lambda'] = Categorical([0] + non_zero_values)
                else:
```

```
                search_spaces['reg_lambda'] = Categorical([0])
        else:
            min_val = min(lambda_l2_values)
            max_val = max(lambda_l2_values)
            search_spaces['reg_lambda'] = Real(min_val, max_val, prior='log-uniform')
    if 'min_gain_to_split' in param_grid:
        min_gain_values = param_grid['min_gain_to_split']
        if 0 in min_gain_values:
            non_zero_values = [v for v in min_gain_values if v > 0]
            if non_zero_values:
                search_spaces['min_split_gain'] = Categorical([0] + non_zero_values)
            else:
                search_spaces['min_split_gain'] = Categorical([0])
        else:
            min_val = min(min_gain_values)
            max_val = max(min_gain_values)
            search_spaces['min_split_gain'] = Real(min_val, max_val, prior='uniform')
    if 'bagging_freq' in param_grid and 'bagging_fraction' in param_grid:
        bagging_freq_values = param_grid['bagging_freq']
        bagging_fraction_values = param_grid['bagging_fraction']
        if min(bagging_fraction_values) < 1.0:
            non_zero_freq = [v for v in bagging_freq_values if v > 0]
            if non_zero_freq:
                search_spaces['bagging_freq'] = Categorical(non_zero_freq)
                print("Note: Removed bagging_freq=0 to avoid warnings with subsample <
1.0")
            else:
                search_spaces['bagging_freq'] = Categorical([1])
                print("Note: Changed bagging_freq to 1 to work with subsample < 1.0")
        else:
            if 0 in bagging_freq_values:
                non_zero_values = [v for v in bagging_freq_values if v > 0]
                if non_zero_values:
                    search_spaces['bagging_freq'] = Categorical([0] + non_zero_values)
                else:
                    search_spaces['bagging_freq'] = Categorical([0])
            else:
                min_val = min(bagging_freq_values)
                max_val = max(bagging_freq_values)
                search_spaces['bagging_freq'] = Integer(min_val, max_val)
    elif 'bagging_freq' in param_grid:
        bagging_freq_values = param_grid['bagging_freq']
        if 0 in bagging_freq_values:
            non_zero_values = [v for v in bagging_freq_values if v > 0]
```

```
                if non_zero_values:
                    search_spaces['bagging_freq'] = Categorical([0] + non_zero_values)
                else:
                    search_spaces['bagging_freq'] = Categorical([0])
            else:
                min_val = min(bagging_freq_values)
                max_val = max(bagging_freq_values)
                search_spaces['bagging_freq'] = Integer(min_val, max_val)
        if 'n_jobs' in param_grid:
            search_spaces['n_jobs'] = Categorical(param_grid['n_jobs'])
        return search_spaces
def analyze_predictions(test_predictions, test_metrics):
    predictions_df = pd.DataFrame(test_predictions)
    corr_matrix = predictions_df.corr()
    error_stats = {}
    for model_name, metrics in test_metrics.items():
        error_stats[model_name] = {
            'RMSE': metrics['RMSE'],
            'MAE': metrics['MAE'],
            'R2': metrics['R2']
        }
    print("\nModel Prediction Correlation Analysis:")
    print(corr_matrix)
    print("\nModel Error Statistics:")
    for model, stats in error_stats.items():
        print(f"\n{model}:")
        for metric, value in stats.items():
            print(f"{metric}: {value:.4f}")
    return corr_matrix, error_stats
def save_shap_results(shap_data, base_path, target_name):
    try:
        import sys
        if shap_data is None:
            print(f"No SHAP data to save ({target_name})")
            return None
        shap_dir = os.path.join(base_path, 'shap_analysis', target_name)
        os.makedirs(shap_dir, exist_ok=True)
        print(f"SHAP data keys: {list(shap_data.keys())}")
        summary = {}
        for model_name, model_shap in shap_data.items():
            model_dir = os.path.join(shap_dir, model_name)
            os.makedirs(model_dir, exist_ok=True)
            print(f"Saving SHAP data for {model_name}, available keys: {list(model_shap.keys())}")
```

```
for values_key in ['shap_values', 'values', 'shap_data']:
    if values_key in model_shap:
        values_path = os.path.join(model_dir, 'shap_values.npy')
        np.save(values_path, model_shap[values_key])
        summary[f"{model_name}_values_path"] = values_path
        print(f"Saved SHAP values to {values_path} (using key: {values_key})")
        break
else:
    print(f"Warning: No SHAP values data found for {model_name}")
if 'feature_names' in model_shap:
    features_path = os.path.join(model_dir, 'feature_names.txt')
    with open(features_path, 'w') as f:
        f.write('\n'.join(model_shap['feature_names']))
    summary[f"{model_name}_features_path"] = features_path
if 'importance' in model_shap:
    importance_path = os.path.join(model_dir, 'feature_importance.csv')
    model_shap['importance'].to_csv(importance_path, index=False)
    summary[f"{model_name}_importance_path"] = importance_path
    if 'feature' in model_shap['importance']:
        top_features = model_shap['importance']['feature'].tolist()[:5]
        for feature in top_features:
            try:
                plt.figure(figsize=(10, 7))
                shap.dependence_plot(
                    feature,
                    model_shap['shap_values'] if 'shap_values' in model_shap else model_shap['values'],
                    model_shap['X_sample'],
                    feature_names=model_shap['feature_names'] if 'feature_names' in model_shap else None,
                    show=False
                )
                plt.tight_layout()
                safe_feature = feature.replace(' ', '_').replace('(', '').replace(')', '').replace('/', '_').replace('\\', '_')
                dep_path = os.path.join(model_dir, f'{safe_feature}_dependence.png')
                plt.savefig(dep_path, dpi=300, bbox_inches='tight')
                plt.close()
                summary[f"{model_name}_{safe_feature}_dependence"] = dep_path
            except:
                pass
if 'X_sample' in model_shap:
```

```
                sample_path = os.path.join(model_dir, 'X_sample.csv')
                model_shap['X_sample'].to_csv(sample_path)
                summary[f"{model_name}_sample_path"] = sample_path
            if 'shap_df' in model_shap:
                df_path = os.path.join(model_dir, 'shap_df.csv')
                model_shap['shap_df'].to_csv(df_path)
                summary[f"{model_name}_shap_df_path"] = df_path
            if 'explainer_type' in model_shap:
                summary[f"{model_name}_explainer"] = model_shap['explainer_type']
        summary_path = os.path.join(shap_dir, 'summary.json')
        with open(summary_path, 'w') as f:
            json.dump(summary, f, indent=4)
        print(f"SHAP analysis results saved to {shap_dir}")
        return summary
    except Exception as e:
        return None
def save_results_to_path(all_results, base_path='results'):
    timestamp = datetime.now().strftime('%Y%m%d_%H%M%S')
    if os.path.isdir(base_path) and 'plots' in os.listdir(base_path):
        save_path = base_path
        print(f"Using existing save path: {save_path}")
    else:
        save_path = os.path.join(base_path, timestamp)
    folders = ['models', 'predictions', 'metrics', 'plots', 'features', 'shap_analysis',
'feature_analysis']
    for folder in folders:
        os.makedirs(os.path.join(save_path, folder), exist_ok=True)
    summary = {}
    for target_name, results in all_results.items():
        target_folders = [
            os.path.join(save_path, 'metrics'),
            os.path.join(save_path, 'predictions'),
            os.path.join(save_path, 'features'),
            os.path.join(save_path, 'models'),
            os.path.join(save_path, 'shap_analysis'),
            os.path.join(save_path, 'feature_analysis')
        ]
        for folder in target_folders:
            os.makedirs(folder, exist_ok=True)
        target_summary = {}
        metrics_path = os.path.join(save_path, 'metrics', f'{target_name}_metrics.json')
        metrics_dict = {
            'model_metrics': {},
            'base_model_weights': {}
```

```
        }
        for model_name, metrics in results['test_metrics'].items():
            if model_name != 'Ensemble':
                model_metrics = {}
                for metric, value in metrics.items():
                    if isinstance(value, tuple) and hasattr(value, '__iter__'):
                        model_metrics[metric] = float(value[0]) if hasattr(value[0], 'dtype') else value[0]
                    else:
                        model_metrics[metric] = float(value) if hasattr(value, 'dtype') else value
                metrics_dict['model_metrics'][model_name] = {
                    'best_metrics': model_metrics,
                    'best_parameters': results['best_params'].get(model_name, {})
                }
        if 'base_model_weights' in results:
            for model_name, weight in results['base_model_weights'].items():
                if isinstance(weight, tuple) and hasattr(weight, '__iter__'):
                    metrics_dict['base_model_weights'][model_name] = float(weight[0])
                else:
                    metrics_dict['base_model_weights'][model_name] = float(weight)
        with open(metrics_path, 'w') as f:
            json.dump(metrics_dict, f, indent=4)
        target_summary['metrics_file'] = metrics_path
        predictions_path = os.path.join(save_path, 'predictions', f'{target_name}_predictions.csv')
        pred_df = pd.DataFrame({
            'true_values': results['y_true']
        })
        for model_name, preds in results['final_predictions'].items():
            pred_df[f'{model_name}_predictions'] = preds
        pred_df.to_csv(predictions_path)
        target_summary['predictions_file'] = predictions_path
        os.makedirs(os.path.join(save_path, 'features'), exist_ok=True)
        if 'feature_importance' in results:
            feature_path = os.path.join(save_path, 'features', f'{target_name}_feature_importance.json')
            feature_dict = {}
            for model, importance in results['feature_importance'].items():
                feature_dict[model] = {}
                for feat, imp in importance.items():
                    if isinstance(imp, tuple) and hasattr(imp, '__iter__'):
                        feature_dict[model][str(feat)] = float(imp[0])
                    else:
```

```
                feature_dict[model][str(feat)] = float(imp)
        with open(feature_path, 'w') as f:
            json.dump(feature_dict, f, indent=4)
        target_summary['feature_importance_file'] = feature_path
    feature_analysis_path = os.path.join(save_path, 'feature_analysis')
    os.makedirs(feature_analysis_path, exist_ok=True)
    if 'detailed_ablation' in results and 'feature_groups' in results:
        if 'plot_feature_group_importance' in globals():
            plot_feature_group_importance(
                results['detailed_ablation'],
                feature_analysis_path,
                target_name
            )
        feature_groups_path = os.path.join(feature_analysis_path,
f'{target_name}_feature_groups.json')
        with open(feature_groups_path, 'w') as f:
            json.dump(results['feature_groups'], f, indent=4)
        target_summary['feature_analysis'] = {
            'group_importance_plot': os.path.join(
                'feature_analysis',
                f'{target_name}_feature_group_importance.png'
            ),
            'feature_groups_file': os.path.join(
                'feature_analysis',
                f'{target_name}_feature_groups.json'
            )
        }
    os.makedirs(os.path.join(save_path, 'models'), exist_ok=True)
    if 'best_params' in results:
        params_path = os.path.join(save_path, 'models',
f'{target_name}_best_params.json')
        with open(params_path, 'w') as f:
            json.dump(results['best_params'], f, indent=4)
        target_summary['best_params_file'] = params_path
    if 'models' in results:
        model_path = os.path.join(save_path, 'models', f'{target_name}_models.pkl')
        with open(model_path, 'wb') as f:
            pickle.dump(results['models'], f)
        target_summary['models_file'] = model_path
    shap_path = os.path.join(save_path, 'shap_analysis')
    os.makedirs(shap_path, exist_ok=True)
    if 'shap_values' in results:
        if 'save_shap_results' in globals():
            shap_summary = save_shap_results(results['shap_values'], save_path,
```

```
target_name)
                target_summary['shap_files'] = shap_summary
        os.makedirs(os.path.join(save_path, 'plots'), exist_ok=True)
        plot_path = os.path.join(save_path, 'plots', f'{target_name}_plots.pdf')
        fig = plt.figure(figsize=(20, 15))
        plt.subplot(2, 2, 1)
        for name, pred in results['final_predictions'].items():
            plt.scatter(results['y_true'], pred, alpha=0.5, label=name)
        plt.plot([results['y_true'].min(), results['y_true'].max()],
                [results['y_true'].min(), results['y_true'].max()],
                'k--', label='Perfect Prediction')
        plt.xlabel('Actual Values')
        plt.ylabel('Predicted Values')
        plt.title(f'{target_name} Predictions vs Actual')
        plt.legend()
        plt.subplot(2, 2, 2)
        for i, (name, pred) in enumerate(results['final_predictions'].items()):
            if i == 0:
                residuals = results['y_true'] - pred
                sns.histplot(residuals, kde=True, label=name)
        plt.axvline(x=0, color='r', linestyle='--')
        plt.xlabel('Residuals')
        plt.ylabel('Frequency')
        plt.title(f'{target_name} Residual Distribution')
        if 'feature_importance' in results:
            plt.subplot(2, 2, 3)
            if 'XGBoost' in results['feature_importance']:
                importances =
pd.Series(results['feature_importance']['XGBoost']).sort_values(ascending=True)
                importances.tail(10).plot(kind='barh')
                plt.title(f'{target_name} Top 10 Feature Importance')
        plt.tight_layout()
        plt.savefig(plot_path)
        plt.close()
        target_summary['plots_file'] = plot_path
        if 'ablation_results' in results and 'detailed_ablation' in results:
            ablation_path = os.path.join(save_path, 'feature_analysis')
            os.makedirs(ablation_path, exist_ok=True)
            ablation_results_path = os.path.join(ablation_path,
f'{target_name}_ablation_results.json')
            ablation_dict = {}
            for model_name, group_results in results['ablation_results'].items():
                ablation_dict[model_name] = {}
                for group_name, scores in group_results.items():
```

```
                    ablation_dict[model_name][group_name] = {}
                    for score_key, score_value in scores.items():
                        if score_key == 'features':
                            ablation_dict[model_name][group_name][score_key] =
list(score_value)
                        elif isinstance(score_value, tuple) and hasattr(score_value,
'__iter__'):
                            ablation_dict[model_name][group_name][score_key] =
float(score_value[0])
                        else:
                            ablation_dict[model_name][group_name][score_key] =
float(score_value) if hasattr(score_value, 'dtype') else score_value
            with open(ablation_results_path, 'w') as f:
                json.dump(ablation_dict, f, indent=4)
            feature_groups_path = os.path.join(ablation_path,
f'{target_name}_feature_groups.json')
            with open(feature_groups_path, 'w') as f:
                json.dump(results['feature_groups'], f, indent=4)
            target_summary['feature_analysis'] = {
                'ablation_results': ablation_results_path,
                'feature_groups': feature_groups_path
            }
        summary[target_name] = target_summary
    config = {
        'timestamp': timestamp,
        'summary': summary,
        'experiment_info': {
            'python_version': platform.python_version(),
            'system_info': platform.platform(),
            'libraries': {
                'sklearn': sklearn.__version__,
                'numpy': np.__version__,
                'pandas': pd.__version__,
                'xgboost': xgb.__version__ if 'xgb' in globals() else 'not_available'
            }
        }
    }
    with open(os.path.join(save_path, 'experiment_summary.json'), 'w') as f:
        json.dump(config, f, indent=4)
    print(f"\nResults saved to: {save_path}")
    return save_path
def split_dataset(X, y, test_size=0.2, random_state=42):
    print("Performing improved stratified sampling...")
    print(f"Target variable statistics: mean={y.mean():.4f}, std={y.std():.4f}, min={y.min():.4f},
```

```
max={y.max():.4f}")
    n_samples = len(y)
    if n_samples < 50:
        n_bins = max(3, n_samples // 10)
    elif n_samples < 200:
        n_bins = max(5, n_samples // 20)
    else:
        n_bins = max(10, n_samples // 30)

    print(f"Sample count: {n_samples}, using {n_bins} stratification bins")
    try:
        y_binned = pd.qcut(y, q=n_bins, labels=False, duplicates='drop')
        binning_method = "quantile binning"
    except:
        try:
            y_binned = pd.cut(y, bins=n_bins, labels=False, duplicates='drop')
            binning_method = "equal-width binning"
        except:
            ranks = y.rank(method='first')
            y_binned = pd.qcut(ranks, q=n_bins, labels=False, duplicates='drop')
            binning_method = "rank-based binning"

    print(f"Using {binning_method} for stratification")
    bin_counts = pd.Series(y_binned).value_counts().sort_index()
    print("Bin sample distribution:")
    for bin_id, count in bin_counts.items():
        print(f"    Bin {bin_id}: {count} samples ({count/n_samples*100:.1f}%)")
    try:
        X_train, X_test, y_train, y_test, y_binned_train, y_binned_test = train_test_split(
            X, y, y_binned,
            test_size=test_size,
            random_state=random_state,
            stratify=y_binned
        )
        print("Successfully completed stratified sampling")
    except Exception as e:
        print(f"Stratified sampling failed: {str(e)}, falling back to random split")
        X_train, X_test, y_train, y_test = train_test_split(
            X, y,
            test_size=test_size,
            random_state=random_state
        )
        y_binned_train = pd.Series([-1] * len(y_train))
        y_binned_test = pd.Series([-1] * len(y_test))
```

```
    train_stats = pd.Series(y_train).describe()
    test_stats = pd.Series(y_test).describe()
    print("\nTrain and test set distribution comparison:")
    comparison = pd.DataFrame({
        'Train': train_stats,
        'Test': test_stats,
        'Difference(%)': ((test_stats - train_stats) / train_stats * 100).round(2)
    })
    print(comparison)
    if -1 not in y_binned_train.values:
        train_bin_dist = y_binned_train.value_counts(normalize=True).sort_index()
        test_bin_dist = y_binned_test.value_counts(normalize=True).sort_index()
        print("\nTrain and test set bin distribution comparison:")
        all_bins = sorted(set(train_bin_dist.index) | set(test_bin_dist.index))
        for bin_id in all_bins:
            train_pct = train_bin_dist.get(bin_id, 0) * 100
            test_pct = test_bin_dist.get(bin_id, 0) * 100
            print(f"  Bin {bin_id}: Train {train_pct:.1f}%, Test {test_pct:.1f}%, Difference {test_pct-train_pct:.1f}%")
    return X_train, X_test, y_train, y_test
def augment_regression_data(X_train, y_train, noise_ratio=0.01, target_ratio=9):
    print(f"Executing improved stratified regression data augmentation (target ratio: {target_ratio}x)...")
    train_size = len(y_train)
    y_mean = y_train.mean()
    y_std = y_train.std()
    y_min = y_train.min()
    y_max = y_train.max()
    y_range = y_max - y_min
    y_skew = y_train.skew()
    print(f"Training set target statistics: mean={y_mean:.4f}, std={y_std:.4f}, range=[{y_min:.4f}, {y_max:.4f}], skew={y_skew:.4f}")
    n_bins = max(6, min(12, train_size // 12))
    try:
        if abs(y_skew) > 1:
            if len(y_train.unique()) < n_bins:
                n_bins = max(3, len(y_train.unique()) - 1)
                print(f"Too few unique values, reducing bins to {n_bins}")
            jittered = y_train + np.random.normal(0, y_std * 1e-6, size=len(y_train))
            train_bins, bin_edges = pd.qcut(jittered, q=n_bins, labels=False, retbins=True, duplicates='drop')
        else:
            train_bins, bin_edges = pd.cut(y_train, bins=n_bins, labels=False, retbins=True, duplicates='drop')
```

```
            binning_method = "adaptive binning"
        except Exception as e:
            print(f"Binning error: {str(e)}, using rank-based binning")
            ranks = y_train.rank(method='first')
            train_bins, bin_edges = pd.qcut(ranks, q=n_bins, labels=False, retbins=True, 
duplicates='drop')
            binning_method = "rank-based binning"
        print(f"Using {binning_method} method to create {n_bins} bins")
        bin_counts = pd.Series(train_bins).value_counts().sort_index()
        print("Training set bin distribution:")
        for bin_id, count in bin_counts.items():
            bin_y = y_train[train_bins == bin_id]
            print(f"  Bin {bin_id}: {count} samples ({count/train_size*100:.1f}%), 
mean={bin_y.mean():.4f}, range=[{bin_y.min():.4f}, {bin_y.max():.4f}]")
        ideal_bin_count = train_size / len(bin_counts)
        X_augmented_list = [X_train.copy()]
        y_augmented_list = [y_train.copy()]
        categorical_cols = [col for col in X_train.columns if col.endswith('_Encoded') or col in 
['Compound_Encoded', 'Plant_Species_Encoded', 'Functional_Group_Encoded'] or 
len(X_train[col].unique()) < 10]
        print(f"Identified {len(categorical_cols)} categorical features: {categorical_cols}")
        numeric_cols = [col for col in X_train.columns if col not in categorical_cols]
        from tensorflow.keras.layers import Input, Dense, Lambda
        from tensorflow.keras.models import Model
        from tensorflow.keras import backend as K
        from tensorflow.keras import regularizers
        import numpy as np
        from sklearn.preprocessing import StandardScaler
        print("Training variational autoencoder (VAE) for data augmentation...")
        X_numeric = X_train[numeric_cols].copy()
        scaler = StandardScaler()
        X_numeric_scaled = scaler.fit_transform(X_numeric)
        input_dim = X_numeric_scaled.shape[1]
        encoding_dim = max(min(input_dim // 2, 10), 2)
        input_layer = Input(shape=(input_dim,))
        hidden_encoder = Dense(encoding_dim * 2, activation='relu')(input_layer)
        z_mean = Dense(encoding_dim)(hidden_encoder)
        z_log_var = Dense(encoding_dim)(hidden_encoder)
        def sampling(args):
            z_mean, z_log_var = args
            batch = K.shape(z_mean)[0]
            dim = K.int_shape(z_mean)[1]
            epsilon = K.random_normal(shape=(batch, dim))
            return z_mean + K.exp(0.5 * z_log_var) * epsilon
```

```
z = Lambda(sampling, output_shape=(encoding_dim,))([z_mean, z_log_var])
decoder_hidden = Dense(encoding_dim * 2, activation='relu')
decoder_output = Dense(input_dim, activation='linear')
hidden_decoder = decoder_hidden(z)
outputs = decoder_output(hidden_decoder)
vae = Model(input_layer, outputs)
reconstruction_loss = K.mean(K.square(input_layer - outputs))
reconstruction_loss *= input_dim
kl_loss = -0.5 * K.sum(1 + z_log_var - K.square(z_mean) - K.exp(z_log_var), axis=-1)
vae_loss = K.mean(reconstruction_loss + kl_loss * 0.1)
vae.add_loss(vae_loss)
vae.compile(optimizer='adam')
encoder = Model(input_layer, [z_mean, z_log_var, z])
decoder_input = Input(shape=(encoding_dim,))
decoder_hidden_output = decoder_hidden(decoder_input)
decoded_output = decoder_output(decoder_hidden_output)
decoder = Model(decoder_input, decoded_output)
vae.fit(X_numeric_scaled, epochs=200, batch_size=64, shuffle=True, verbose=0)
print(f"VAE trained with latent dimension: {encoding_dim}")
n_iterations = 50
total_added = 0
for iteration in range(n_iterations):
    print(f"\nStarting augmentation iteration {iteration+1}/{n_iterations}")
    current_size = sum(len(df) for df in X_augmented_list)
    if current_size >= train_size * target_ratio:
        print(f"Target ratio {target_ratio}x reached (iteration {iteration+1})")
        break
    for bin_id in range(n_bins):
        if bin_id not in bin_counts.index:
            continue
        bin_count = bin_counts.get(bin_id, 0)
        bin_mask = (train_bins == bin_id)
        bin_X = X_train[bin_mask]
        bin_y = y_train[bin_mask]
        if bin_count < ideal_bin_count:
            augment_ratio = min(20.0, ideal_bin_count / bin_count * 1.2)
        else:
            augment_ratio = 1.5
        samples_to_add = int(bin_count * (augment_ratio - 1) + 0.5)
        max_to_add = min(20 * bin_count, 1000)
        samples_to_add = min(samples_to_add, max_to_add)
        if samples_to_add <= 0:
            continue
        print(f"Bin {bin_id}: original samples={bin_count}, target
```

```
augmentation={bin_count+samples_to_add} (ratio={augment_ratio:.2f}x)")
            bin_y_mean = bin_y.mean()
            bin_y_std = bin_y.std() if len(bin_y) > 1 else y_std * 0.1
            bin_y_min = bin_y.min()
            bin_y_max = bin_y.max()
            n_smote = int(samples_to_add * 0.5)
            if n_smote > 0 and len(bin_X) >= 2:
                n_pairs = min(n_smote, len(bin_X) * 2)
                created_pairs = 0
                for _ in range(100):
                    if created_pairs >= n_pairs:
                        break
                    idx1, idx2 = np.random.choice(len(bin_X), 2, replace=False)
                    alpha = 0.25 + np.random.random() * 0.6
                    x_new = pd.Series(index=X_train.columns)
                    for col in X_train.columns:
                        if col in categorical_cols:
                            x_new[col] = bin_X.iloc[np.random.choice([idx1, idx2])][col]
                        else:
                            x_new[col] = alpha * bin_X.iloc[idx1][col] + (1 - alpha) *
bin_X.iloc[idx2][col]
                    y_new = alpha * bin_y.iloc[idx1] + (1 - alpha) * bin_y.iloc[idx2]
                    for col in numeric_cols:
                        col_std = X_train[col].std()
                        if col_std > 0:
                            x_new[col] += np.random.normal(0, col_std * 0.001)
                    y_new += np.random.normal(0, bin_y_std * 0.005)
                    X_augmented_list.append(pd.DataFrame([x_new],
columns=X_train.columns))
                    y_augmented_list.append(pd.Series([y_new]))
                    total_added += 1
                    created_pairs += 1
            n_vae = int(samples_to_add * 0.5)
            if n_vae > 0 and len(bin_X) > 0:
                bin_X_numeric = bin_X[numeric_cols].copy()
                bin_X_scaled = scaler.transform(bin_X_numeric)
                z_mean_bin, z_log_var_bin, _ = encoder.predict(bin_X_scaled)
                for i in range(n_vae):
                    idx = np.random.randint(0, len(bin_X))
                    base_mean = z_mean_bin[idx % len(z_mean_bin)]
                    base_log_var = z_log_var_bin[idx % len(z_log_var_bin)]
                    epsilon = np.random.normal(0, 1, size=encoding_dim)
                    latent_sample = base_mean + np.exp(0.5 * base_log_var) * epsilon
                    latent_noise = np.random.normal(0, 0.15, size=encoding_dim)
```

```
                    perturbed_latent = latent_sample + latent_noise
                    decoded_sample = decoder.predict(np.array([perturbed_latent]))[0]
                    decoded_sample_orig_scale = scaler.inverse_transform(decoded_sample.reshape(1, -1))[0]
                    x_sample = bin_X.iloc[idx].copy()
                    for j, col in enumerate(numeric_cols):
                        x_sample[col] = decoded_sample_orig_scale[j]
                    for col in categorical_cols:
                        x_sample[col] = bin_X.iloc[idx][col]
                    y_pred = bin_y.iloc[idx] + np.random.normal(0, bin_y_std * 0.02)
                    y_pred = np.clip(y_pred, bin_y_min, bin_y_max)
                    X_augmented_list.append(pd.DataFrame([x_sample], columns=X_train.columns))
                    y_augmented_list.append(pd.Series([y_pred]))
                    total_added += 1
        print(f"After iteration {iteration+1}: Added {total_added} samples, current total: {sum(len(df) for df in X_augmented_list)}")
        current_size = sum(len(df) for df in X_augmented_list)
        if current_size >= train_size * target_ratio:
            print(f"Target ratio {target_ratio}x reached (iteration {iteration+1})")
            break
    X_augmented = pd.concat(X_augmented_list, axis=0)
    y_augmented = pd.concat(y_augmented_list, axis=0)
    X_augmented = X_augmented.reset_index(drop=True)
    y_augmented = y_augmented.reset_index(drop=True)
    if X_augmented.isna().any().any():
        print(f"Filling {X_augmented.isna().sum().sum()} NaN values in augmented data")
        X_augmented = X_augmented.fillna(X_train.mean())
    print("\nValidating quality of augmented data distribution...")
    try:
        if bin_edges is not None:
            augmented_bins = pd.cut(y_augmented, bins=bin_edges, labels=range(len(bin_edges)-1), include_lowest=True)
            augmented_bin_counts = pd.Series(augmented_bins).value_counts(normalize=True).sort_index() * 100
            original_bin_counts = bin_counts / bin_counts.sum() * 100
            print("\nDistribution comparison before and after augmentation (%):")
            all_bins = sorted(set(original_bin_counts.index) | set(augmented_bin_counts.index))
            print(f"{'Bin':>5} | {'Original(%)':>12} | {'Augmented(%)':>12} | {'Change(%)':>10}")
            print("-" * 50)
            for bin_id in all_bins:
                orig_pct = original_bin_counts.get(bin_id, 0)
```

```
                    aug_pct = augmented_bin_counts.get(bin_id, 0)
                    change = aug_pct - orig_pct
                    print(f"{bin_id:5d} | {orig_pct:12.2f} | {aug_pct:12.2f} | {change:+10.2f}")
        except Exception as e:
            print(f"Error calculating distribution comparison: {str(e)}")
        y_aug_stats = pd.Series(y_augmented).describe()
        y_orig_stats = pd.Series(y_train).describe()
        print("\nTarget variable statistics comparison before and after augmentation:")
        stat_comparison = pd.DataFrame({
            'Original Training': y_orig_stats,
            'After Augmentation': y_aug_stats,
            'Relative Change(%)': ((y_aug_stats - y_orig_stats) / y_orig_stats * 100).round(2)
        })
        print(stat_comparison)
        mean_diff_pct = abs((y_augmented.mean() - y_train.mean()) / y_train.mean() * 100)
        std_diff_pct = abs((y_augmented.std() - y_train.std()) / y_train.std() * 100)
        if mean_diff_pct > 10 or std_diff_pct > 20:
            print(f"\nWarning: Augmented data statistics differ significantly from original data:")
            print(f"    - Mean difference: {mean_diff_pct:.2f}% (should be <10%)")
            print(f"    - Std difference: {std_diff_pct:.2f}% (should be <20%)")
            print("    - Applying correction to match original distribution...")
            y_aug_std = (y_augmented - y_augmented.mean()) / y_augmented.std()
            y_augmented = y_aug_std * y_train.std() + y_train.mean()
            print("\nTarget variable statistics comparison after correction:")
            y_aug_stats = pd.Series(y_augmented).describe()
            stat_comparison = pd.DataFrame({
                'Original Training': y_orig_stats,
                'After Correction': y_aug_stats,
                'Relative Change(%)': ((y_aug_stats - y_orig_stats) / y_orig_stats * 100).round(2)
            })
            print(stat_comparison)
        print(f"\nData augmentation completed:")
        print(f" Original training samples: {train_size}")
        print(f" Augmented samples: {len(y_augmented)} (+{len(y_augmented)-train_size})")
        print(f" Augmentation ratio: {len(y_augmented)/train_size:.2f}x")
        return X_augmented, y_augmented
def create_error_chart(message, save_path):
    try:
        plt.figure(figsize=(10, 6))
        plt.text(0.5, 0.5, f"Cannot create hydroponic RCF analysis visualization:\n{message}",
                    ha='center', va='center', fontsize=14,
                    bbox=dict(facecolor='white', alpha=0.8, boxstyle='round,pad=1'))
        plt.axis('off')
        os.makedirs(save_path, exist_ok=True)
```

```
            error_file = os.path.join(save_path, 'rcf_analysis_error.png')
            plt.savefig(error_file, dpi=300, bbox_inches='tight')
            print(f"Saved error chart to: {error_file}")
            os.makedirs('results', exist_ok=True)
            root_error_file = os.path.join('results', 'rcf_analysis_error.png')
            plt.savefig(root_error_file, dpi=300, bbox_inches='tight')
            print(f"Also saved error chart to: {root_error_file}")
            plt.close()
            return False
    except:
            print("Failed to create error chart")
            return False
def calc_correlation_p_value(r, n):
    t = r * np.sqrt((n - 2) / (1 - r**2))
    p = 2 * stats.t.sf(np.abs(t), n-2)
    return p
def detect_duplicate_features(X, threshold=0.85):
    corr = X.corr().abs()
    upper = corr.where(np.triu(np.ones(corr.shape), k=1).astype(bool))
    duplicates = [(col1, col2) for col1 in upper.columns
                  for col2 in upper.index
                  if upper.loc[col2, col1] > threshold]

    return duplicates
def apply_genetic_programming(X_train_augmented, y_train_augmented, X_test):
    from gplearn.genetic import SymbolicRegressor
    print("Running Genetic Programming on augmented data with higher complexity...")
    est_gp_augmented = SymbolicRegressor(
        population_size=2000,
        generations=100,
        stopping_criteria=0.005,
        p_crossover=0.7,
        p_subtree_mutation=0.2,
        p_hoist_mutation=0.05,
        p_point_mutation=0.05,
        max_samples=0.9,
        verbose=1,
        parsimony_coefficient=0.0005,
        random_state=42,
        n_jobs=-1,
        function_set=('add', 'sub', 'mul', 'div', 'log', 'sqrt', 'sin', 'cos', 'abs')
    )
    est_gp_augmented.fit(X_train_augmented.values, y_train_augmented.values)
    y_pred_gp_augmented = est_gp_augmented.predict(X_test.values)
```

```
    symbolic_expr = str(est_gp_augmented._program)
    return est_gp_augmented, y_pred_gp_augmented, symbolic_expr
def apply_MFTEC(X_train_features, y_train_augmented, X_test_features):
    from sklearn.linear_model import LassoCV, RidgeCV, ElasticNetCV
    from sklearn.svm import SVR
    from sklearn.ensemble import StackingRegressor
    from xgboost import XGBRegressor
    from catboost import CatBoostRegressor
    import lightgbm as lgb
    print("Using improved MFTEC approach for augmented data...")
    interpretable_model = ElasticNetCV(
        cv=5,
        max_iter=5000,
        tol=1e-5,
        l1_ratio=[0.1, 0.5, 0.7, 0.9, 0.99, 1.0],
        n_jobs=-1,
        alphas=np.logspace(-6, 1, 50),
        random_state=42
    )
    base_models = [
        ('lasso', LassoCV(cv=5, max_iter=2000, tol=1e-4, selection='random', n_jobs=-1,
alphas=np.logspace(-6, 1, 20))),
        ('ridge', RidgeCV(cv=5, alphas=np.logspace(-6, 6, 13))),
        ('elastic', ElasticNetCV(cv=5, max_iter=2000, l1_ratio=[0.1, 0.5, 0.7, 0.9, 0.99], tol=1e-
4, n_jobs=-1)),
        ('lgbm', lgb.LGBMRegressor(n_estimators=100, num_leaves=31, max_depth=5,
learning_rate=0.05, subsample=0.8)),
        ('xgb', XGBRegressor(n_estimators=100, max_depth=5, learning_rate=0.05,
subsample=0.8, colsample_bytree=0.8)),
        ('catboost', CatBoostRegressor(iterations=100, depth=5, learning_rate=0.05,
subsample=0.8, verbose=0, thread_count=-1))
    ]
    stacking_model = StackingRegressor(
        estimators=base_models,
        final_estimator=SVR(C=1.0, epsilon=0.1, gamma='scale'),
        cv=5,
        n_jobs=-1
    )
    interpretable_model.fit(X_train_features, y_train_augmented)
    stacking_model.fit(X_train_features, y_train_augmented)
    y_pred_mftec_augmented = stacking_model.predict(X_test_features)
    return interpretable_model, stacking_model, y_pred_mftec_augmented
def apply_HSIE(X_selected_features_aug, y_train_augmented, X_test_selected_aug):
    from sklearn.ensemble import GradientBoostingRegressor, ExtraTreesRegressor
```

```
from sklearn.linear_model import ElasticNetCV, RidgeCV
import lightgbm as lgb
print("Running HSIE on augmented data with higher complexity...")
def create_stacked_model(X, y):
    gbm = GradientBoostingRegressor(
        n_estimators=100, max_depth=5,
        learning_rate=0.05, subsample=0.8,
        random_state=42
    )
    lgb_params = {
        'objective': 'regression',
        'metric': 'rmse',
        'verbosity': -1,
        'boosting_type': 'gbdt',
        'learning_rate': 0.05,
        'feature_fraction': 0.8,
        'bagging_fraction': 0.8,
        'num_leaves': 31
    }
    et = ExtraTreesRegressor(
        n_estimators=100, max_depth=10,
        random_state=42, n_jobs=-1
    )
    gbm.fit(X, y)
    lgb_train = lgb.Dataset(X, y)
    lgb_model = lgb.train(lgb_params, lgb_train, num_boost_round=100)
    et.fit(X, y)
    gbm_pred = gbm.predict(X)
    lgb_pred = lgb_model.predict(X)
    et_pred = et.predict(X)
    meta_features = np.column_stack([gbm_pred, lgb_pred, et_pred])
    meta_model = RidgeCV(alphas=np.logspace(-6, 6, 13), cv=5)
    meta_model.fit(meta_features, y)
    return {
        'gbm': gbm,
        'lgb_model': lgb_model,
        'et': et,
        'meta_model': meta_model
    }
def predict_with_stacked_model(model_dict, X):
    gbm_pred = model_dict['gbm'].predict(X)
    lgb_pred = model_dict['lgb_model'].predict(X)
    et_pred = model_dict['et'].predict(X)
    meta_features = np.column_stack([gbm_pred, lgb_pred, et_pred])
```

```
            return model_dict['meta_model'].predict(meta_features)
        stacked_model = create_stacked_model(X_selected_features_aug, y_train_augmented)
        y_pred_hsie_augmented = predict_with_stacked_model(stacked_model,
X_test_selected_aug)
        model_augmented = ElasticNetCV(
            cv=5,
            max_iter=5000,
            tol=1e-5,
            l1_ratio=[0.1, 0.5, 0.7, 0.9, 0.99, 1.0],
            n_jobs=-1,
            alphas=np.logspace(-6, 2, 20),
            random_state=42
        )
        model_augmented.fit(X_selected_features_aug, y_train_augmented)
        return model_augmented, stacked_model, y_pred_hsie_augmented
def apply_all_augmented_models(X_train_augmented, y_train_augmented, X_test,
X_train_features, X_test_features, X_selected_features_aug, X_test_selected_aug,
feature_names):
        gp_model, gp_predictions, gp_equation =
apply_genetic_programming(X_train_augmented, y_train_augmented, X_test)
        mapped_gp_eq = gp_equation
        for i, col in enumerate(feature_names):
            mapped_gp_eq = mapped_gp_eq.replace(f"X{i}", f"({col})")
        mftec_model, mftec_stacked_model, mftec_predictions = apply_MFTEC(X_train_features,
y_train_augmented, X_test_features)
        coefs = mftec_model.coef_
        intercept = mftec_model.intercept_
        significant_indices = np.where(np.abs(coefs) > 1e-6)[0]
        if len(significant_indices) < 3:
            significant_indices = np.argsort(np.abs(coefs))[-15:]
        equation_terms = []
        for idx in significant_indices:
            if idx < len(feature_names):
                coef = coefs[idx]
                if abs(coef) > 0:
                    name = feature_names[idx]
                    equation_terms.append(f"{coef:.8f}×{name}")
        if equation_terms:
            mftec_eq = " + ".join(equation_terms)
            if intercept != 0:
                mftec_eq += f" + {intercept:.8f}"
        else:
            mftec_eq = f"{intercept:.8f}"
        hsie_model, hsie_stacked_model, hsie_predictions =
```

```
apply_HSIE(X_selected_features_aug, y_train_augmented, X_test_selected_aug)
    return gp_model, gp_predictions, mapped_gp_eq, mftec_model, mftec_stacked_model, 
mftec_predictions, mftec_eq, hsie_model, hsie_stacked_model, hsie_predictions
def calculate_regression_metrics(y_true, y_pred):
    y_true = np.array(y_true)
    y_pred = np.array(y_pred)
    mse = mean_squared_error(y_true, y_pred)
    rmse = np.sqrt(mse)
    mae = mean_absolute_error(y_true, y_pred)
    r2 = r2_score(y_true, y_pred)
    pearson_corr = pearsonr(y_true, y_pred)
    mask = y_true != 0
    if mask.sum() > 0:
        mape = np.mean(np.abs((y_true[mask] - y_pred[mask]) / y_true[mask])) * 100
    else:
        mape = np.nan
    std_ratio = rmse / np.std(y_true) if np.std(y_true) > 0 else np.nan
    metrics = {
        'MSE': mse,
        'RMSE': rmse,
        'MAE': mae,
        'R2': r2,
        'Pearson_r': pearson_corr,
        'MAPE': mape,
        'RMSE/Std_Ratio': std_ratio
    }
    return metrics
def visualize_data_augmentation(X_train, y_train, X_train_augmented, y_train_augmented, 
save_path):
    statistics_summary = {}
    augmentation_dir = os.path.join(save_path, 'data_augmentation')
    os.makedirs(augmentation_dir, exist_ok=True)
    original_color = '#1f77b4'
    augmented_color = '#ff7f0e'
    plt.figure(figsize=(10, 6))
    sns.kdeplot(y_train, fill=True, color=original_color, alpha=0.5, label='Original Data')
    sns.kdeplot(y_train_augmented, fill=True, color=augmented_color, alpha=0.5, 
label='Augmented Data')
    plt.title('logRCF Distribution Comparison', fontsize=20)
    plt.xlabel('logRCF Value', fontsize=20)
    plt.ylabel('Density', fontsize=20)
    plt.xticks(fontsize=20)
    plt.yticks(fontsize=20)
    plt.legend(fontsize=20)
```

```
    plt.grid(True, linestyle='--', alpha=0.7)
    plt.tight_layout()
    plt.savefig(os.path.join(augmentation_dir, 'target_density_comparison.png'), dpi=300)
    plt.close()
    plt.figure(figsize=(8, 6))
    boxprops_orig = dict(linewidth=2, color=original_color)
    boxprops_aug = dict(linewidth=2, color=augmented_color)
    flierprops_orig = dict(marker='o', markerfacecolor=original_color, markersize=6,
markeredgecolor='none')
    flierprops_aug = dict(marker='o', markerfacecolor=augmented_color, markersize=6,
markeredgecolor='none')
    whiskerprops_orig = dict(linewidth=2, color=original_color)
    whiskerprops_aug = dict(linewidth=2, color=augmented_color)
    bplot1 = plt.boxplot([y_train], positions=[1], widths=0.6, patch_artist=True,
                         boxprops=boxprops_orig, flierprops=flierprops_orig,
whiskerprops=whiskerprops_orig)
    bplot2 = plt.boxplot([y_train_augmented], positions=[2], widths=0.6, patch_artist=True,
                         boxprops=boxprops_aug, flierprops=flierprops_aug,
whiskerprops=whiskerprops_aug)
    for patch in bplot1['boxes']:
        patch.set_facecolor(original_color)
        patch.set_alpha(0.6)
    for patch in bplot2['boxes']:
        patch.set_facecolor(augmented_color)
        patch.set_alpha(0.6)
    plt.xticks([1, 2], ['Original', 'Augmented'], fontsize=20)
    plt.yticks(fontsize=20)
    plt.title('logRCF Distribution Boxplot', fontsize=20)
    plt.ylabel('logRCF Value', fontsize=20)
    plt.grid(True, linestyle='--', alpha=0.7)
    plt.tight_layout()
    plt.savefig(os.path.join(augmentation_dir, 'target_boxplot_comparison.png'), dpi=300)
    plt.close()
    plt.figure(figsize=(10, 6))
    bins = np.linspace(min(min(y_train), min(y_train_augmented)),
                       max(max(y_train), max(y_train_augmented)), 30)
    plt.hist(y_train, bins=bins, alpha=0.6, color=original_color, label='Original Data')
    plt.hist(y_train_augmented, bins=bins, alpha=0.6, color=augmented_color,
label='Augmented Data')
    plt.title('logRCF Value Histogram Comparison', fontsize=20)
    plt.xlabel('logRCF Value', fontsize=20)
    plt.ylabel('Frequency', fontsize=20)
    plt.xticks(fontsize=20)
    plt.yticks(fontsize=20)
```

```
    plt.legend(fontsize=20)
    plt.grid(True, linestyle='--', alpha=0.7)
    plt.tight_layout()
    plt.savefig(os.path.join(augmentation_dir, 'target_histogram_comparison.png'), dpi=300)
    plt.close()
    try:
        max_samples = min(3000, len(X_train))
        idx_train = np.random.choice(len(X_train), min(max_samples, len(X_train)), replace=False)
        idx_augmented = np.random.choice(len(X_train_augmented), min(max_samples, len(X_train_augmented)), replace=False)
        X_combined = np.vstack([X_train.iloc[idx_train], X_train_augmented.iloc[idx_augmented]])
        y_combined = np.concatenate([y_train.iloc[idx_train], y_train_augmented.iloc[idx_augmented]])
        is_augmented = np.concatenate([np.zeros(len(idx_train)), np.ones(len(idx_augmented))])
        tsne = TSNE(n_components=2, random_state=42, perplexity=min(30, len(X_combined)-1))
        X_tsne = tsne.fit_transform(X_combined)
        plt.figure(figsize=(10, 8))
        mask_orig = is_augmented == 0
        mask_aug = is_augmented == 1
        X_orig_tsne = X_tsne[mask_orig]
        X_aug_tsne = X_tsne[mask_aug]
        scatter3 = plt.scatter(X_orig_tsne[:, 0], X_orig_tsne[:, 1],
                               c=y_combined[mask_orig], cmap='viridis', alpha=0.7, s=50,
                               edgecolors='w', linewidths=0.5, label='Original')
        scatter4 = plt.scatter(X_aug_tsne[:, 0], X_aug_tsne[:, 1],
                               c=y_combined[mask_aug], cmap='plasma', alpha=0.5, s=20,
                               marker='s', edgecolors='none', label='Augmented')
        kmeans_orig_tsne = KMeans(n_clusters=min(5, len(X_orig_tsne)), random_state=42).fit(X_orig_tsne)
        kmeans_aug_tsne = KMeans(n_clusters=min(5, len(X_aug_tsne)), random_state=42).fit(X_aug_tsne)
        def draw_ellipse(position, covariance, ax=None, **kwargs):
            ax = ax or plt.gca()
            if covariance.shape == (2, 2):
                U, s, Vt = np.linalg.svd(covariance)
                angle = np.degrees(np.arctan2(U[1, 0], U[0, 0]))
                width, height = 2 * np.sqrt(s)
            else:
```

```
                angle = 0
                width, height = 2 * np.sqrt(covariance)
            for nsig in [1, 2]:
                ellipse = Ellipse(position, nsig * width, nsig * height, angle=angle, **kwargs)
                ax.add_patch(ellipse)
        for i in range(kmeans_orig_tsne.n_clusters):
            points = X_orig_tsne[kmeans_orig_tsne.labels_ == i]
            if len(points) > 1:
                mean = points.mean(axis=0)
                cov = np.cov(points, rowvar=False)
                draw_ellipse(mean, cov, alpha=0.2, color='blue')
        for i in range(kmeans_aug_tsne.n_clusters):
            points = X_aug_tsne[kmeans_aug_tsne.labels_ == i]
            if len(points) > 1:
                mean = points.mean(axis=0)
                cov = np.cov(points, rowvar=False)
                draw_ellipse(mean, cov, alpha=0.2, color='orange')
        plt.title('t-SNE Projection of Feature Space', fontsize=20)
        plt.xlabel('t-SNE Dimension 1', fontsize=20)
        plt.ylabel('t-SNE Dimension 2', fontsize=20)
        plt.xticks(fontsize=20)
        plt.yticks(fontsize=20)
        cbar = plt.colorbar(scatter3)
        cbar.set_label('logRCF Value', fontsize=20)
        cbar.ax.tick_params(labelsize=20)
        from matplotlib.lines import Line2D
        legend_elements = [
            Line2D([0], [0], marker='o', color='w', markerfacecolor='darkblue',
markersize=10, label='Original Data'),
            Line2D([0], [0], marker='s', color='w', markerfacecolor='darkred', markersize=8,
label='Augmented Data')
        ]
        legend = plt.legend(handles=legend_elements, loc='upper left', fontsize=16,
framealpha=0.7)
        legend.get_frame().set_facecolor('white')
        plt.tight_layout()
        plt.savefig(os.path.join(augmentation_dir, 'tsne_feature_space.png'), dpi=300)
        plt.close()
    except Exception as e:
        print(f"t-SNE visualization error: {e}. Skipping t-SNE plot.")
    important_features = list(X_train.columns)[:min(6, len(X_train.columns))]
    feature_stats = {}
    for feature in important_features:
        orig_values = X_train[feature].values
```

```
        aug_values = X_train_augmented[feature].values
        feature_stats[feature] = {
            'orig_mean': np.mean(orig_values),
            'aug_mean': np.mean(aug_values),
            'orig_std': np.std(orig_values),
            'aug_std': np.std(aug_values),
            'orig_min': np.min(orig_values),
            'aug_min': np.min(aug_values),
            'orig_max': np.max(orig_values),
            'aug_max': np.max(aug_values)
        }
    ks_statistic, ks_pvalue = stats.ks_2samp(y_train, y_train_augmented)
    mw_statistic, mw_pvalue = stats.mannwhitneyu(y_train, y_train_augmented)
    original_stats = {
        'mean': np.mean(y_train),
        'median': np.median(y_train),
        'std': np.std(y_train),
        'min': np.min(y_train),
        'max': np.max(y_train),
        'range': np.max(y_train) - np.min(y_train),
        'count': len(y_train)
    }
    augmented_stats = {
        'mean': np.mean(y_train_augmented),
        'median': np.median(y_train_augmented),
        'std': np.std(y_train_augmented),
        'min': np.min(y_train_augmented),
        'max': np.max(y_train_augmented),
        'range': np.max(y_train_augmented) - np.min(y_train_augmented),
        'count': len(y_train_augmented)
    }
    statistics_summary['target_variable'] = {
        'original_stats': original_stats,
        'augmented_stats': augmented_stats,
        'ks_test': {'statistic': ks_statistic, 'p_value': ks_pvalue},
        'mann_whitney_test': {'statistic': mw_statistic, 'p_value': mw_pvalue}
    }
    feature_statistics = {}
    for i, feature in enumerate(important_features):
        orig_values = X_train[feature].values
        aug_values = X_train_augmented[feature].values
        safe_feature_name = re.sub(r'[^\w\-_.]', '_', feature)
        ks_stat, ks_p = stats.ks_2samp(orig_values, aug_values)
        mw_stat, mw_p = stats.mannwhitneyu(orig_values, aug_values)
```

```
        plt.figure(figsize=(8, 5))
        sns.kdeplot(orig_values, fill=True, color=original_color, alpha=0.5, label='Original
Data')
        sns.kdeplot(aug_values, fill=True, color=augmented_color, alpha=0.5,
label='Augmented Data')
        plt.title(f'Feature: {feature} - Distribution Comparison', fontsize=20)
        plt.xlabel(feature, fontsize=20)
        plt.ylabel('Density', fontsize=20)
        plt.xticks(fontsize=20)
        plt.yticks(fontsize=20)
        plt.legend(fontsize=20)
        plt.grid(True, linestyle='--', alpha=0.7)
        plt.tight_layout()
        plt.savefig(os.path.join(augmentation_dir,
f'feature_{safe_feature_name}_density.png'), dpi=300)
        plt.close()
        plt.figure(figsize=(8, 5))
        violin_data = [orig_values, aug_values]
        violin_parts = plt.violinplot(violin_data, showmeans=True, showmedians=True,
showextrema=True)
        for pc, color in zip(violin_parts['bodies'], [original_color, augmented_color]):
            pc.set_facecolor(color)
            pc.set_alpha(0.7)
        for partname in ['cmeans', 'cmedians', 'cbars', 'cmins', 'cmaxes']:
            if partname in violin_parts:
                vp = violin_parts[partname]
                vp.set_edgecolor('black')
                vp.set_linewidth(1)
        plt.xticks([1, 2], ['Original', 'Augmented'], fontsize=20)
        plt.yticks(fontsize=20)
        plt.title(f'Feature: {feature} - Violin Plot', fontsize=20)
        plt.grid(True, linestyle='--', alpha=0.7)
        plt.tight_layout()
        plt.savefig(os.path.join(augmentation_dir,
f'feature_{safe_feature_name}_violin.png'), dpi=300)
        plt.close()
        plt.figure(figsize=(8, 5))
        x = np.linspace(min(min(orig_values), min(aug_values)),
                        max(max(orig_values), max(aug_values)), 100)
        plt.plot(x, x, color='gray', linestyle='--', label='y=x')
        percentiles = np.linspace(0, 100, 21)
        q_orig = np.percentile(orig_values, percentiles)
        q_aug = np.percentile(aug_values, percentiles)
        plt.scatter(q_orig, q_aug, color='purple', alpha=0.7, s=40)
```

```
            plt.xlabel('Original Data Quantiles', fontsize=20)
            plt.ylabel('Augmented Data Quantiles', fontsize=20)
            plt.xticks(fontsize=20)
            plt.yticks(fontsize=20)
            plt.title(f'Feature: {feature} - Q-Q Plot', fontsize=20)
            plt.grid(True, linestyle='--', alpha=0.5)
            plt.tight_layout()
            plt.savefig(os.path.join(augmentation_dir, f'feature_{safe_feature_name}_qq.png'),
dpi=300)
            plt.close()
            feature_statistics[feature] = {
                'ks_test': {'statistic': ks_stat, 'p_value': ks_p},
                'mw_test': {'statistic': mw_stat, 'p_value': mw_p},
                'original_mean': np.mean(orig_values),
                'augmented_mean': np.mean(aug_values),
                'original_std': np.std(orig_values),
                'augmented_std': np.std(aug_values),
                'percent_change_mean': ((np.mean(aug_values) - np.mean(orig_values)) /
np.mean(orig_values)) * 100 if np.mean(orig_values) != 0 else float('inf'),
                'percent_change_std': ((np.std(aug_values) - np.std(orig_values)) /
np.std(orig_values)) * 100 if np.std(orig_values) != 0 else float('inf')
            }
        statistics_summary['features'] = feature_statistics
        with open(os.path.join(augmentation_dir, 'augmentation_statistics.json'), 'w') as f:
            import json
            json.dump(statistics_summary, f, indent=4, default=str)
        summary_text = "# Data Augmentation Statistical Analysis\n\n"
        summary_text += "## Target Variable (logRCF) Analysis\n\n"
        summary_text += f"- **Original data**: n={original_stats['count']},
mean={original_stats['mean']:.4f}, std={original_stats['std']:.4f}\n"
        summary_text += f"- **Augmented data**: n={augmented_stats['count']},
mean={augmented_stats['mean']:.4f}, std={augmented_stats['std']:.4f}\n\n"
        summary_text += "### Statistical Test Results\n\n"
        summary_text += f"- **Kolmogorov-Smirnov test**: statistic={ks_statistic:.4f}, p-
value={ks_pvalue:.6f} "
        summary_text += f"({'Significant difference' if ks_pvalue < 0.05 else 'No significant
difference'})\n"
        summary_text += f"- **Mann-Whitney U test**: statistic={mw_statistic:.4f}, p-
value={mw_pvalue:.6f} "
        summary_text += f"({'Significant difference' if mw_pvalue < 0.05 else 'No significant
difference'})\n\n"
        summary_text += "## Feature-by-Feature Analysis\n\n"
        for feature, stats_dict in feature_statistics.items():
            summary_text += f"### Feature: {feature}\n\n"
```

```
        summary_text += f"- **Original data**: mean={stats_dict['original_mean']:.4f}, std={stats_dict['original_std']:.4f}\n"
        summary_text += f"- **Augmented data**: mean={stats_dict['augmented_mean']:.4f}, std={stats_dict['augmented_std']:.4f}\n"
        summary_text += f"- **Percent change**: mean change={stats_dict['percent_change_mean']:.2f}%, std change={stats_dict['percent_change_std']:.2f}%\n\n"
        summary_text += "**Statistical Tests**:\n"
        summary_text += f"- K-S test: statistic={stats_dict['ks_test']['statistic']:.4f}, p-value={stats_dict['ks_test']['p_value']:.6f} "
        summary_text += f"({'Significant difference' if stats_dict['ks_test']['p_value'] < 0.05 else 'No significant difference'})\n"
        summary_text += f"- M-W test: statistic={stats_dict['mw_test']['statistic']:.4f}, p-value={stats_dict['mw_test']['p_value']:.6f} "
        summary_text += f"({'Significant difference' if stats_dict['mw_test']['p_value'] < 0.05 else 'No significant difference'})\n\n"
    with open(os.path.join(augmentation_dir, 'augmentation_summary.md'), 'w') as f:
        f.write(summary_text)
    print("\nData augmentation analysis completed. Results saved to: " + augmentation_dir)
    return statistics_summary
def main():
    tf.keras.backend.clear_session()
    import gc
    gc.collect()
    print("Starting data processing and model training...")
    timestamp = datetime.now().strftime('%Y%m%d_%H%M%S')
    base_save_path = os.path.join('results', timestamp)
    subdirs = ['plots', 'models', 'metrics', 'shap_analysis', 'feature_analysis', 'hydroponic_analysis', 'predictions', 'learning_curves']
    for subdir in subdirs:
        os.makedirs(os.path.join(base_save_path, subdir), exist_ok=True)
    print(f"Results will be saved to: {base_save_path}")
    import logging
    os.environ["RAY_DEDUP_LOGS_ALLOW_REGEX"] = r"(R2|RMSE|MSE|MAE|score|loss|epoch|batch|step)"
    os.environ["RAY_DEDUP_LOGS_AGG_WINDOW_S"] = "10"
    os.environ["RAY_ROTATION_MAX_BYTES"] = str(50 * 1024 * 1024)
    os.environ["RAY_ROTATION_BACKUP_COUNT"] = "5"
    try:
        ray.shutdown()
    except:
        pass
    ray.init(log_to_driver=False, ignore_reinit_error=True)
    logger = logging.getLogger("pfas_ml_pipeline")
```

```
    logger.setLevel(logging.INFO)
    df = pd.read_excel(r'Datacollection.xlsx', nrows=617)
    le_plant = LabelEncoder()
    le_compound = LabelEncoder()
    le_functional = LabelEncoder()
    df['Plant_Species_Encoded'] = le_plant.fit_transform(df['Plant Species'])
    df['Compound_Encoded'] = le_compound.fit_transform(df['Compound'])
    df['Functional_Group_Encoded'] = le_functional.fit_transform(df['Functional Group'])
    base_features = [
        'Molecular Weight (g/mol)', 'pKa', 'log Kow',
        ' Exposure time    (hrs)', 'Plant_Species_Encoded',
        'Compound_Encoded', 'Functional_Group_Encoded'
    ]
    base_df = df[base_features].copy()
    for col in base_features:
        if col in base_df.columns:
            base_df[col] = pd.to_numeric(base_df[col], errors='coerce')
    futures = []
    for compound in df['Compound'].unique():
        if compound in pfas_smiles:
            mol = Chem.MolFromSmiles(pfas_smiles[compound])
            rdkit_future = calculate_rdkit_descriptors.remote(mol)
            cid_future = get_pubchem_cid.remote(compound)
            prop_future = get_pubchem_properties.remote(cid_future)
            futures.extend([rdkit_future, prop_future])
    results = ray.get(futures)
    desc_data = []
    for i in range(0, len(results), 2):
        rdkit_desc = results[i]
        pubchem_desc = results[i+1]
        compound_desc = {}
        if rdkit_desc and isinstance(rdkit_desc, dict):
            compound_desc.update(rdkit_desc)
        if pubchem_desc and isinstance(pubchem_desc, dict):
            pubchem_props = {f'pubchem_{k}': v for k, v in pubchem_desc.items() if k != 
'CID'}
            compound_desc.update(pubchem_props)
        desc_data.append(compound_desc)
    desc_df = pd.DataFrame(desc_data)
    numeric_cols = []
    for col in desc_df.columns:
        try:
            desc_df[col] = pd.to_numeric(desc_df[col], errors='coerce')
            numeric_cols.append(col)
```

```
        except:
            print(f"Cannot convert column {col} to numeric type")
    desc_df = desc_df[numeric_cols]
    X = pd.concat([base_df, desc_df], axis=1)
    X = X.fillna(X.mean())
    X = X.reset_index(drop=True)
    feature_groups = {
        'molecular_basic': ['Molecular Weight (g/mol)', 'pKa', 'log Kow'],
        'plant': ['Plant_Species_Encoded'],
        'compound_category': ['Compound_Encoded', 'Functional_Group_Encoded'],
        'exposure': [' Exposure time    (hrs)'],
        'topological': [
            'Chi0n', 'Chi1n', 'Chi2n', 'Chi3n', 'Chi4n',
            'Chi0v', 'Chi1v', 'Chi2v', 'Chi3v', 'Chi4v',
            'BertzCT'
        ],
        'atom_bonds': [
            'nC', 'nF', 'nO', 'nS', 'nN', 'nP',
            'NumAtoms', 'NumBonds', 'NumRotatableBonds',
            'AromaticRings', 'Rings'
        ],
        'surface_properties': [
            'TPSA',
            'HBA', 'HBD',
            'HBondDonorCount', 'HBondAcceptorCount'
        ],
        'stereochemistry': [
            'AtomStereoCount', 'BondStereoCount',
            'DefinedAtomStereoCount', 'UndefinedAtomStereoCount',
            'DefinedBondStereoCount', 'UndefinedBondStereoCount'
        ],
        'physicochemical': [
            'LogP', 'CrippenLogP', 'CrippenMR',
            'Complexity', 'HeavyAtomCount'
        ],
        'estate': [col for col in X.columns if col.startswith('EState_VSA')],
        'derived_features': [
            col for col in X.columns if any(x in col.lower() for x in
            ['ratio', 'index', 'interaction', 'normalized', 'weighted', 'diff', 'rate',
             'log', 'sqrt', 'q1', 'q2', 'q3', 'q4', 'mol', 'mw', 'permeability',
             'squared', 'binding', 'score', 'factor', 'chain', 'polarity', 'degree', 'exp',
'complexity',
             'balance', 'lipophilicity', 'effect', 'connectivity', 'consensus', 'deviation',
             'range', 'std', 'sum', 'mean', 'distribution', 'potential', 'utilization', 'adsorption'])
```

```
            ]
        }
        all_classified_features = set()
        for group in feature_groups.values():
            all_classified_features.update(group)
        unclassified_features = set(X.columns) - all_classified_features
        if unclassified_features:
            feature_groups['other'] = list(unclassified_features)
        target_variables = {
            'logRCF': df['RCF (L/g)'].copy()
        }
        all_results = {}
        for target_name, y in target_variables.items():
            print(f"\nProcessing target variable: {target_name}")
            print("=" * 50)
            print(f"Original target variable samples: {len(y)}")
            print(f"Missing values in target variable: {y.isna().sum()}")
            impute_features = ['Molecular Weight (g/mol)', 'pKa', 'log Kow', ' Exposure time
(hrs)']
            impute_df = pd.concat([X[impute_features], y.rename('target')], axis=1)
            imputer = IterativeImputer(
                estimator=RandomForestRegressor(n_estimators=100, random_state=42),
                random_state=42,
                max_iter=10
            )
            imputed_values = imputer.fit_transform(impute_df)
            y = pd.Series(imputed_values[:, -1], index=y.index)
            y = preprocess_target(y)
            X_valid = X.copy()
            X_engineered                    =                    engineer_features_parallel(X_valid,
exclude_columns=[f'{target_name} (L/g)'])
            X_engineered = X_engineered.replace([np.inf, -np.inf], np.nan)
            means = X_engineered.mean()
            X_engineered = X_engineered.fillna(means)
            duplicates = detect_duplicate_features(X_engineered, threshold=0.99)
            if duplicates:
                print(f"Detected {len(duplicates)} potential duplicate features:")
                for col1, col2 in duplicates[:10]:
                    print(f" - {col1} and {col2} have correlation above 0.99")
                features_to_remove = []
                for col1, col2 in duplicates:
                    if len(col1) > len(col2) or ' ' in col1:
                        features_to_remove.append(col1)
                    else:
```

```
                    features_to_remove.append(col2)
            features_to_remove = list(set(features_to_remove))
            print(f"Will remove {len(features_to_remove)} duplicate features")
            X_engineered = X_engineered.drop(columns=features_to_remove)
            print(f"Number of features after removing duplicates: {X_engineered.shape[1]}")
        print("\nAnalyzing feature engineering effects...")
        selected_features = select_features_parallel(X_engineered, y, threshold=0.001)
        print(f"Number of selected features: {len(selected_features)}")
        X_selected = X_engineered[selected_features]
        print("\nVisualizing relationships between selected features...")
        visualize_features(X_selected, y, f"{target_name}_Selected",
                           save_path=os.path.join(base_save_path, 'feature_analysis'))
        scaler = StandardScaler()
        X_scaled = pd.DataFrame(
            scaler.fit_transform(X_selected),
            columns=X_selected.columns
        )
        X_train, X_test, y_train, y_test = split_dataset(X_scaled, y, test_size=0.25,
random_state=42)
        print(f"Training set size: {len(X_train)}, Test set size: {len(X_test)}")
        print(X_train.columns)
        transform_params = {}
        param_grids = {
            'CatBoost': {
                'iterations': [500, 1000],
                'depth': [5,10],
                'learning_rate': [0.05, 0.1],
                'l2_leaf_reg': [5, 10],
                'random_seed': [42]
            },
            'XGBoost': {
                'n_estimators': [500, 1000],
                'max_depth': [10, 25],
                'learning_rate': [0.01, 0.05, 0.1],
                'min_child_weight': [5, 10],
                'subsample': [0.6, 0.8, 1.0],
                'colsample_bytree': [0.6, 0.8, 1.0],
                'n_jobs': [-1]
            },
            'LightGBM': {
                'num_boost_round': [500, 1000],
                'max_depth': [10, 25],
                'num_leaves': [50, 70],
                'n_jobs': [-1],
```

```
                'verbose': [-1]
            },
            'RandomForest': {
                'n_estimators': [500, 1000],
                'max_depth': [15, 50],
                'min_samples_split': [2, 15],
                'n_jobs': [-1],
                'random_state': [42]
            }
        }
        print("\nPerforming stratified regression data augmentation...")
        X_train_augmented, y_train_augmented = augment_regression_data(X_train,
y_train, noise_ratio=0.01)
        print(f"Training set size before augmentation: {len(X_train)}, after:
{len(X_train_augmented)}")
        print("\nPerforming cross-validation for traditional machine learning models...")
        kf = KFold(n_splits=5, shuffle=True, random_state=42)
        cv_results = {}
        print("\nTraining models on augmented training set...")
        best_models, best_params_dict = grid_search_models(X_train_augmented,
y_train_augmented, param_grids)
        shap_results = {}
        for model_name, model in best_models.items():
            if model_name in ['CatBoost', 'XGBoost', 'LightGBM', 'RandomForest']:
                print(f"\nCalculating SHAP values for {model_name}...")
                shap_result = calculate_shap_values(
                    model,
                    X_train_augmented[selected_features],
                    feature_names=selected_features,
                    sample_size=min(1000, len(X_train_augmented))
                )
                if shap_result is not None:
                    shap_results[model_name] = shap_result
        if shap_results:
            print("\nSaving SHAP analysis results...")
            shap_dir = os.path.join(base_save_path, 'shap_analysis')
            for model_name, shap_result in shap_results.items():
                try:
                    model_type = model_name.lower()
                    if 'X_sample' in shap_result and 'shap_values' in shap_result:
                        X_for_plot = shap_result['X_sample']
                        shap_values_for_plot = shap_result['shap_values']
                        feature_names = shap_result['feature_names']
                        if isinstance(shap_values_for_plot, list):
```

```
                    if len(shap_values_for_plot) > 0:
                        expected_rows = shap_values_for_plot[0].shape[0]
                    else:
                        raise ValueError("SHAP values list is empty")
                else:
                    expected_rows = shap_values_for_plot.shape[0]
                actual_rows = X_for_plot.shape[0]
                if expected_rows != actual_rows:
                    raise ValueError(f"SHAP values row count ({expected_rows})
doesn't match feature matrix row count ({actual_rows})")
                print(f"Drawing SHAP summary plot for {model_name} using
{X_for_plot.shape[0]} samples")
                plt.figure(figsize=(12, 16))
                shap.summary_plot(
                    shap_values_for_plot,
                    X_for_plot,
                    feature_names=feature_names,
                    plot_type="dot",
                    show=False,
                    color_bar=True,
                    max_display=25,
                    plot_size=(12, 16)
                )
                plt.tight_layout()
                plot_path = os.path.join(shap_dir,
f'{model_type}_{target_name}_shap_summary.png')
                plt.savefig(plot_path, dpi=300, bbox_inches='tight')
                plt.close()
                plt.figure(figsize=(12, 8))
                shap.summary_plot(
                    shap_values_for_plot,
                    X_for_plot,
                    feature_names=feature_names,
                    plot_type="bar",
                    show=False
                )
                plt.tight_layout()
                bar_path = os.path.join(shap_dir,
f'{model_type}_{target_name}_shap_importance.png')
                plt.savefig(bar_path, dpi=300, bbox_inches='tight')
                plt.close()
                if 'importance' in shap_result:
                    top_features =
shap_result['importance']['feature'].tolist()[:5]
```

```
                        for feature in top_features:
                            try:
                                plt.figure(figsize=(10, 7))
                                shap.dependence_plot(
                                    feature,
                                    shap_values_for_plot,
                                    X_for_plot,
                                    feature_names=feature_names,
                                    show=False
                                )
                                plt.tight_layout()
                                dep_path = os.path.join(shap_dir,
f'{model_type}_{target_name}_{feature}_dependence.png')
                                plt.savefig(dep_path, dpi=300,
bbox_inches='tight')
                                plt.close()
                            except Exception as dep_error:
                                print(f"Error creating dependence plot for
{feature}: {str(dep_error)}")
                else:
                    print(f"SHAP results missing required data: 'X_sample' or
'shap_values' keys don't exist")
                    print(f"Available keys: {list(shap_result.keys())}")
            except Exception as e:
                print(f"Error saving SHAP results for {model_name}: {str(e)}")
                import traceback
                traceback.print_exc()
    test_metrics = {}
    valid_feature_groups = validate_feature_groups(X_selected, feature_groups)
    print("\nEvaluating all models on test set...")
    test_predictions = {}
    feature_importance = {}
    for model_name, model in best_models.items():
        try:
            test_pred = model.predict(X_test)
            test_predictions[model_name] = pd.Series(test_pred, index=y_test.index)
            test_metrics[model_name] = calculate_regression_metrics(y_test,
test_pred)
            print(f"\n{model_name} test set evaluation results:")
            for metric, value in test_metrics[model_name].items():
                if isinstance(value, tuple) and hasattr(value, '__iter__'):
                    print(f" {metric}: {value[0]:.4f}")
                else:
                    print(f" {metric}: {value:.4f}")
```

```
if hasattr(model, 'feature_importances_'):
    feature_importance[model_name] = dict(zip(selected_features,
model.feature_importances_))
    importance_df = pd.DataFrame({
        'feature': selected_features,
        'importance': model.feature_importances_
    }).sort_values('importance', ascending=False)
    top_features = importance_df.head(20)
    plt.figure(figsize=(14, 10))
    if model_name == 'CatBoost':
        colors = plt.cm.YlOrRd(np.linspace(0.4, 0.9, len(top_features)))
    elif model_name == 'XGBoost':
        colors = plt.cm.YlGnBu(np.linspace(0.4, 0.9, len(top_features)))
    elif model_name == 'LightGBM':
        colors = plt.cm.RdPu(np.linspace(0.4, 0.9, len(top_features)))
    elif model_name == 'RandomForest':
        colors = plt.cm.BuGn(np.linspace(0.4, 0.9, len(top_features)))
    else:
        colors = plt.cm.cividis(np.linspace(0.4, 0.9, len(top_features)))
    plt.barh(top_features['feature'], top_features['importance'],
color=colors)
    plt.title(f'{model_name} feature importance for {target_name}',
fontsize=18)
    plt.xlabel('Importance', fontsize=16)
    plt.ylabel('Feature', fontsize=16)
    plt.gca().invert_yaxis()
    plt.tight_layout()
    plt.savefig(os.path.join(base_save_path, 'feature_analysis',
f'{model_name}_{target_name}_feature_importance.png'))
    plt.close()
plt.figure(figsize=(8, 6))
plt.rcParams.update({'font.size': 16})
plt.scatter(y_test, test_pred, alpha=0.7)
plt.plot([y_test.min(), y_test.max()], [y_test.min(), y_test.max()], 'k--')
plt.xlabel('Actual Values', fontsize=16)
plt.ylabel('Predicted Values', fontsize=16)
r2_value = test_metrics[model_name]["R2"]
if isinstance(r2_value, tuple) and hasattr(r2_value, '__iter__'):
    r2_value = r2_value[0]
plt.title(f'{model_name} Model (R^2 = {r2_value:.4f})', fontsize=18)
plt.grid(True, alpha=0.3)
plt.tight_layout()
plt.savefig(os.path.join(base_save_path, 'plots',
f'{target_name}_{model_name}_scatter.png'), dpi=300)
```

```
            plt.close()
        except Exception as e:
            print(f"Error evaluating {model_name}: {str(e)}")
weights = {}
for model_name, metrics in test_metrics.items():
    r2_value = metrics['R2']
    if isinstance(r2_value, tuple) and hasattr(r2_value, '__iter__'):
        r2_value = r2_value[0]
    weights[model_name] = r2_value
all_results[target_name] = {
    'y_train': y_train,
    'y_test': y_test,
    'y_true': y_test,
    'final_predictions': test_predictions,
    'feature_importance': feature_importance,
    'test_metrics': test_metrics,
    'selected_features': selected_features,
    'scaler': scaler,
    'transform_params': transform_params,
    'feature_groups': valid_feature_groups,
    'shap_values': shap_results,
    'best_params': best_params_dict,
    'traditional_models_cv_results': cv_results
}
models_r2 = {}
for model, metrics in test_metrics.items():
    r2_value = metrics['R2']
    if isinstance(r2_value, tuple) and hasattr(r2_value, '__iter__'):
        r2_value = r2_value[0]
    models_r2[model] = r2_value
models_rmse = {}
for model, metrics in test_metrics.items():
    rmse_value = metrics['RMSE']
    if isinstance(rmse_value, tuple) and hasattr(rmse_value, '__iter__'):
        rmse_value = rmse_value[0]
    models_rmse[model] = rmse_value
models_mae = {}
for model, metrics in test_metrics.items():
    mae_value = metrics['MAE']
    if isinstance(mae_value, tuple) and hasattr(mae_value, '__iter__'):
        mae_value = mae_value[0]
    models_mae[model] = mae_value
plt.figure(figsize=(14, 15))
plt.rcParams.update({'font.size': 16})
```

```
        plt.subplot(3, 1, 1)
        plt.bar(models_r2.keys(), models_r2.values())
        plt.title(f'Coefficient of Determination (R²) for each model ({target_name})',
fontsize=18)
        plt.ylabel('R²', fontsize=16)
        plt.ylim([0, 1])
        for i, (model, r2) in enumerate(models_r2.items()):
            plt.text(i, r2 + 0.02, f"{r2:.3f}", ha='center', fontsize=16)
        plt.subplot(3, 1, 2)
        plt.bar(models_rmse.keys(), models_rmse.values())
        plt.title(f'Root Mean Square Error (RMSE) for each model ({target_name})',
fontsize=18)
        plt.ylabel('RMSE', fontsize=16)
        for i, (model, rmse) in enumerate(models_rmse.items()):
            plt.text(i, rmse + 0.02, f"{rmse:.3f}", ha='center', fontsize=16)
        plt.subplot(3, 1, 3)
        plt.bar(models_mae.keys(), models_mae.values())
        plt.title(f'Mean Absolute Error (MAE) for each model ({target_name})', fontsize=18)
        plt.ylabel('MAE', fontsize=16)
        for i, (model, mae) in enumerate(models_mae.items()):
            plt.text(i, mae + 0.02, f"{mae:.3f}", ha='center', fontsize=16)
        plt.tight_layout()
        plt.savefig(os.path.join(base_save_path, 'plots',
f'{target_name}_model_performance_comparison.png'))
        plt.close()
        print("\nSummary of all model cross-validation metrics:")
        print("-" * 80)
        print(f"{'Model':<15} {'CV R²':<15} {'CV RMSE':<15} {'CV MAE':<15}")
        print("-" * 80)
        for model_name, metrics in cv_results.items():
            print(f"{model_name:<15} {metrics['R2']:<15.4f} {metrics['RMSE']:<15.4f}
{metrics['MAE']:<15.4f}")
        print("-" * 80)
    def visualize_results(results_dict):
        for target, results in results_dict.items():
            metrics = results.get('test_metrics', {})
            models = list(metrics.keys())
            r2_values = []
            for model in models:
                r2_value = metrics[model]['R2']
                if isinstance(r2_value, tuple) and hasattr(r2_value, '__iter__'):
                    r2_values.append(r2_value[0])
                else:
                    r2_values.append(r2_value)
```

```
        plt.figure(figsize=(12, 8))
        plt.bar(models, r2_values)
        plt.title(f'R^2  Comparison for {target}', fontsize=20)
        plt.ylabel('R^2 ', fontsize=16)
        plt.ylim(0, 1)
        plt.xticks(rotation=45, fontsize=14)
        for i, v in enumerate(r2_values):
            plt.text(i, v+0.02, f"{v:.2f}", ha='center', fontsize=14)
        plt.tight_layout()
        plt.savefig(os.path.join(base_save_path, 'plots', f'{target}_r2_comparison.png'),
dpi=300)
        plt.close()
        rmse_values = []
        for model in models:
            rmse_value = metrics[model]['RMSE']
            if isinstance(rmse_value, tuple) and hasattr(rmse_value, '__iter__'):
                rmse_values.append(rmse_value[0])
            else:
                rmse_values.append(rmse_value)
        plt.figure(figsize=(12, 8))
        plt.bar(models, rmse_values)
        plt.title(f'RMSE Comparison for {target}', fontsize=20)
        plt.ylabel('RMSE', fontsize=16)
        plt.xticks(rotation=45, fontsize=14)
        for i, v in enumerate(rmse_values):
            plt.text(i, v+0.02, f"{v:.2f}", ha='center', fontsize=14)
        plt.tight_layout()
        plt.savefig(os.path.join(base_save_path, 'plots',
f'{target}_rmse_comparison.png'), dpi=300)
        plt.close()
        y_true = results.get('y_true')
        predictions = results.get('final_predictions', {})
        for model_name, y_pred in predictions.items():
            plt.figure(figsize=(10, 8))
            plt.scatter(y_true, y_pred, alpha=0.7)
            plt.plot([min(y_true), max(y_true)], [min(y_true), max(y_true)], 'r--')
            r2 = metrics.get(model_name, {}).get('R2', 0)
            if isinstance(r2, tuple) and hasattr(r2, '__iter__'):
                r2 = r2[0]
            plt.title(f'{model_name} Predictions vs True Values (R^2  = {r2:.4f})',
fontsize=18)
            plt.xlabel('True Values', fontsize=16)
            plt.ylabel('Predicted Values', fontsize=16)
            plt.tight_layout()
```

```
                plt.savefig(os.path.join(base_save_path,                              'plots',
f'{target}_{model_name}_scatter.png'), dpi=300)
                plt.close()
                plt.figure(figsize=(10, 8))
                residuals = y_true - y_pred
                plt.scatter(y_pred, residuals, alpha=0.7)
                plt.axhline(y=0, color='r', linestyle='--')
                plt.title(f'{model_name} Residuals Plot', fontsize=18)
                plt.xlabel('Predicted Values', fontsize=16)
                plt.ylabel('Residuals', fontsize=16)
                plt.tight_layout()
                plt.savefig(os.path.join(base_save_path,                              'plots',
f'{target}_{model_name}_residuals.png'), dpi=300)
                plt.close()
            best_model_index = np.argmax(r2_values)
            best_model_name = models[best_model_index]
            plt.figure(figsize=(10, 8))
            best_predictions = predictions[best_model_name]
            plt.scatter(y_true, best_predictions, alpha=0.7)
            plt.plot([min(y_true), max(y_true)], [min(y_true), max(y_true)], 'r--')
            plt.title(f'Best     Model:     {best_model_name}     (R     2          =
{r2_values[best_model_index]:.4f})', fontsize=18)
            plt.xlabel('True Values', fontsize=16)
            plt.ylabel('Predicted Values', fontsize=16)
            plt.tight_layout()
            plt.savefig(os.path.join(base_save_path,                                  'plots',
f'{target}_best_model_predictions.png'), dpi=300)
            plt.close()
    visualize_results({target_name: all_results[target_name]})
    print(f"Saving experiment results to: {base_save_path}")
    save_results_to_path(all_results, base_save_path)
    return all_results, base_save_path
if __name__ == "__main__":
    try:
        all_results , save_path = main()
    except Exception as e:
        print(f"ERROR: {str(e)}")
        print("SPECIFIC ERROR INFORMATION:")
        traceback.print_exc()
    finally:
        ray.shutdown()
```
